# Investigation of the Ionic Liquid Graphene Electric Double Layer in Supercapacitors Using Constant Potential Simulations

**DOI:** 10.3390/nano10112181

**Published:** 2020-11-01

**Authors:** Baris Demir, Debra J. Searles

**Affiliations:** 1 Centre for Theoretical and Computational Molecular Science, The Australian Institute for Bioengineering and Nanotechnology, The University of Queensland, Brisbane, QLD 4072, Australia; 2 School of Chemistry and Molecular Biosciences, The University of Queensland, Brisbane, QLD 4072, Australia

**Keywords:** supercapacitors, ionic liquids, graphene electrode, electric double layer, charge/discharge mechanism, molecular dynamics simulations

## Abstract

In this work, we investigate the effect of the cation structure on the structure and dynamics of the electrode–electrolyte interface using molecular dynamics simulations. A constant potential method is used to capture the behaviour of 1-ethyl-3-methylimidazolium bis (trifluoromethane)sulfonimide ([C${}_{2}$mim][NTf${}_{2}$]) and butyltrimethylammonium bis(trifluoromethane) sulfonimide ([N${}_{4,1,1,1}$][NTf${}_{2}$]) ionic liquids at varying potential differences applied across the supercapacitor. We find that the details of the structure in the electric double layer and the dynamics differ significantly, yet the charge profile and capacitance do not vary greatly. For the systems considered, charging results in the rearrangement and reorientation of ions within ∼1 nm of the electrode rather than the diffusion of ions to/from the bulk region. This occurs on timescales of O(10 ns) for the ionic liquids considered, and depends on the viscosity of the fluid.

## 1. Introduction

In today’s modern society, a wide range of electronic products, including mobile phones, laptops, cameras, pacemakers, hearing aids and electric vehicles, heavily rely on the energy storage technology behind them. Among promising candidates as future energy storage devices are batteries and electric double-layer supercapacitors (EDLSC). A striking difference between these two systems is that no chemical reactions occur on the electrodes of an EDLSC and energy is stored via ion adsorption on the electrodes, whereas energy is generated through chemical reactions involving the electrodes of a battery. Batteries are suitable for long-term energy storage whereas EDLSCs are ideal for repeated short-term power delivery, such as repetitive breaking in electric vehicles. In general, batteries possess high energy density whereas EDLSCs have high power density. However, energy storage devices often need to meet both power density and energy density requirements, so developing devices that achieve both is of importance. To advance the utility of EDLCS, it is vital to understand the molecular-level formation of charge layers in the vicinity of electrodes, or the electric double layers (EDLs) as this is a key factor for their performance. We will refer to EDLSCs as supercapacitors in the rest of this paper since we are not considering pseudocapacitors.

**Figure 1:**
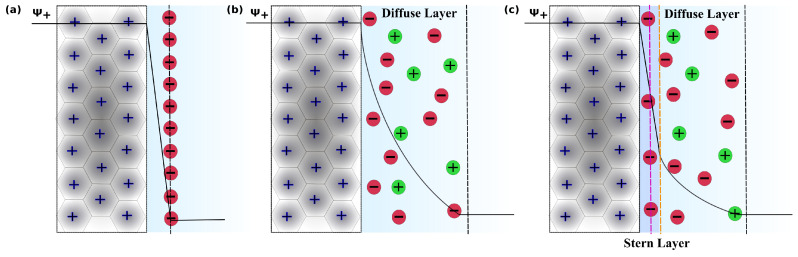
Different descriptions of the electric double layer (EDL) over time. (**a**) The Helmholtz model, (**b**) the Gouy–Chapman model, and (**c**) the Gouy–Chapman–Stern model. The Stern layer is composed of the Inner Helmholtz plane (pink dashed line) and Outer Helmholtz plane (orange dashed line). $\Psi {}^{+}$ represents the potential as a function of distance from the positive electrode.

As well as modifications of the surface chemistry and topological characteristics of electrode materials, the electrolyte can be altered to improve a supercapacitor’s performance. Supercapacitor technologies rely on the EDL that forms at the electrode–electrolyte interface (Figure 1). In 1853, Helmholtz described an EDL as a set of two adjacent layers of charges with opposite signs formed near an electrode surface (Figure 1a) [1]. Gouy and Chapman [2] updated the molecular-level picture of the EDL proposed by Helmholtz as it did not lead to the observed variation in the capacitance with potential difference applied across the cell or the ion concentration at the electrode–electrolyte interface (Figure 1b). The model resulted in an exponential decrease in electric potential away from the electrified electrode surface, rather than the linear decrease resulting from Helmholtz’s model. It accounted for the thermal motion of ions that prevented them from accumulating on the electrified surface and let them sample a larger region, which is called Gouy’s diffuse layer. A combined model was proposed by Otto Stern in 1924 [3], which was built on its predecessors (Figure 1c). The Stern model considered the coexistence of both a compact layer (Stern layer, composed of an inner Helmholtz plane and outer Helmholtz plane) and the diffuse layer. Since then, various models have been introduced to take into account interactions between ions in solution, solvents, molecular structure, etc.

Room temperature ionic liquids (ILs) are liquids consisting of only cations and anions. They are of particular interest for use in supercapacitors due to their low volatility, high ion density and wide electrochemical window. Interesting structures can form in the EDL of ILs due to the different size and structure of the anions, their interaction with the electrodes and with each other. This means that even without charge applied to the electrodes, distinct layers of charge can form near the electrode surface in many IL electrolytes. On application of a potential, over-screening and crowding of ions are observed. In general, the classical models described above do not represent the structures in ILs well. In addition, the high viscosity and charge density of ILs mean that the charge/discharge mechanism is likely to differ from that in lower viscosity electrolytes where the ion density is lower and mobility is higher.

The structure of the EDL and its change on charge/discharge is one key factor determining the ultimate performance (e.g., energy density and power density) of a supercapacitor. Because extensions to the standard model are necessary to gain a molecular-level understanding of the formation of the EDL, further work is required [4]. To this end, experimental techniques, such as atomic force microscopy (AFM) [5,6] and X-ray reflectivity [7] can be used. For example, Black et al. [6] used AFM to investigate the layer formation at a mica surface that was in contact with various ionic liquids (ILs). In a similar work, Begić et al. [8] studied various ILs on electrified, highly oriented pyrolytic graphene (HOPG) and captured the effect of applied potential on the layer formation at the HOPG–IL interface using AFM. Jurado and Espinosa-Marzel [9] combined AFM and Raman microspectroscopy to obtain an understanding of an IL on graphene and various other techniques have provided great insights [4]. However, despite the usefulness of these techniques in providing information on the EDL, they cannot readily provide characterisation with molecular-level resolution. Moreover, factors such as the type and size of the AFM tip used to sample the electrode–IL interface has an influence on the measured force profiles. Therefore, computational methods have been useful for a better understanding of the EDL of ILs [10,11].

The structure of the EDL influences the performance of a supercapacitor. However, the mobility of ions, which are related to the electrolyte viscosity or diffusivity, also plays a role, particularly on the charge/discharge rate. Computer simulations, such as molecular dynamics (MD) simulations, can be used to gain an insight into the atomic-level formation of layers in EDLs. In MD simulations, two techniques have been commonly used to model the charged electrodes: a fixed charge method (FCM) and a constant potential method (CPM) [12,13,14]. In the FCM, the atoms of the electrode are assigned partial charges which are kept constant during a simulation, so the presence of ions at the electrode–electrolyte interface does not affect the charges on the electrode. On the other hand, in CPM simulations, the charges on the electrode are allowed to vary in response to local charge fluctuations, and the potential difference across the supercapacitor is kept constant. Each electrode has the same potential, with an opposite sign. For example, a potential difference, ΔΨ, of 4 V assumes there are two electrodes with potentials of −2 V and +2 V. In both methods, during the charging process the ions redistribute near the electrode to form a steady EDL at the electrolyte-electrode interface. During the CPM simulations the partial atomic charges of electrode atoms are allowed to fluctuate in response to the change in concentration/composition of ions in the region from the electrode. Electrical energy is stored via the electrostatic adsorption of ions found in the EDLs on the electrode due to the charge on the electrode surface.

Recently, various groups have reported the use of the CPM to investigate the molecular-level formation of EDLs in the vicinity of electrodes. For example, Noh and Jung [15] studied the 1-ethyl-3-methylimidazolium thiocyanate ([C${}_{2}$mim][SCN]) IL confined between graphene electrodes. These authors applied various potential difference across the simulation cell, with values between 0 and 4 V, to investigate the charge/discharge dynamics of the supercapacitor at 350 K. In a similar study, Fang and Smolyanitsky [16] studied an IL mixture of 1-ethyl-3-methylimidazolium cations ([C${}_{2}$mim]${}^{+}$), with bis(trifluoromethylsulfonyl)imide ([NTf${}_{2}$]${}^{-}$) and tetrafluoroborate ([BF${}_{4}$]${}^{-}$) anions, and graphene electrodes using the FCM at 400 K. These authors varied the ratio of anions ([NTf${}_{2}$]${}^{-}$ and [BF${}_{4}$]${}^{-}$) to elucidate the effect of the anion on the capacitive performance, and they found that the integral capacitance of the cell increased with an increase in the mole fraction of [BF]${}_{4}$${}^{-}$ in the electrolyte.

In this work, we investigate the effect of the cation structure and dynamics on the layering in the electrode-to-electrolyte interface (or EDLs) using molecular dynamics (MD) simulations by studying two ionic liquid electrolytes with planar graphene electrodes. The ionic liquids are 1-ethyl-3-methylimidazolium bis(trifluoromethylsulfonyl)imide ([C${}_{2}$mim][NTf${}_{2}$]) and butyltrimethylammonium bis(trifluoromethylsulfonyl)imide ([N${}_{4,1,1,1}$][NTf${}_{2}$]). They were selected for consideration because they have the same anion, but different cations and quite different viscosities (32.06 and 112.90 mPa s, respectively, at 298 K [17]). We used the CPM implemented in LAMMPS [18] to capture the dynamics of [C${}_{2}$mim][NTf${}_{2}$] and [N${}_{4,1,1,1}$][NTf${}_{2}$] ILs with varying potential differences applied to the supercapacitor. We also investigated the patterns of ILs that form on the electrode surface with varied applied potential and determined the capacitance of the supercapacitors.

## 2. Models and Simulation Methods

### 2.1. Sample Preparation Procedure

**Figure 2:**
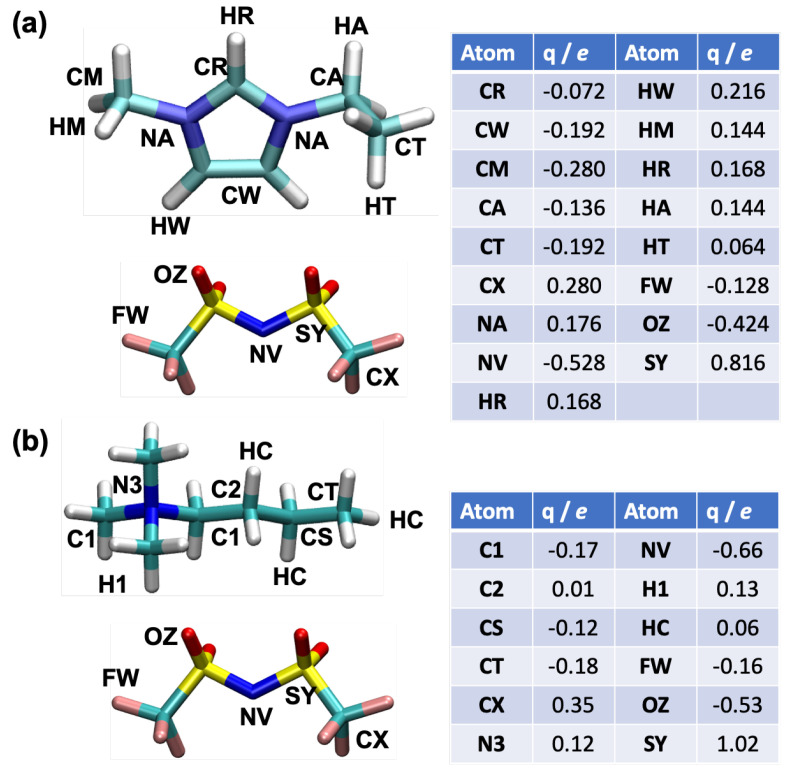
Chemical structure and partial atomic charges (*q*) for the atoms of ions in the ionic liquids (**a**) [C${}_{2}$mim][NTf${}_{2}$] [21], (**b**) [N${}_{4,1,1,1}$][NTf${}_{2}$] [20]. The atomic sites CR, NV and N3 are used as the reference points in the mass density distribution calculations.

**Figure 3:**
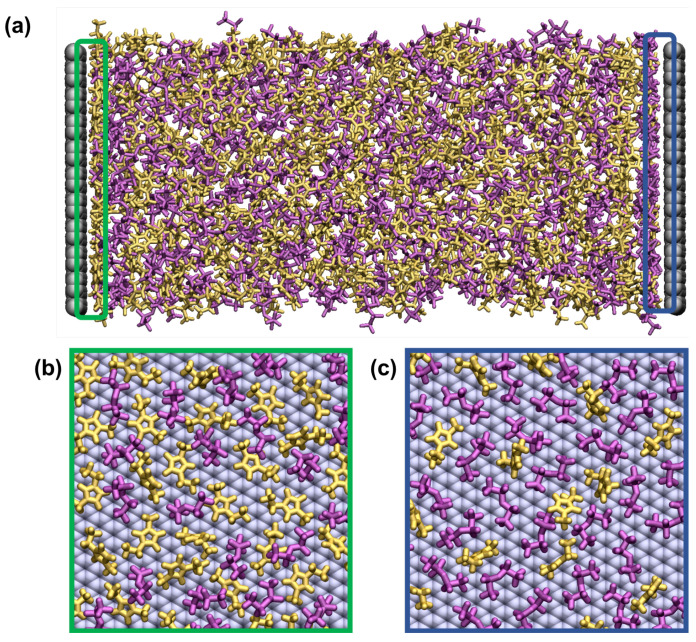
(**a**) A snapshot of [C${}_{2}$mim][NTf${}_{2}$] system taken after 10 ns of a constant potential simulation with ΔΨ=4. A view of the ions that have any atom within 4 Å of of the electrodes for the same system with (**b**) negative electrode and (**c**) positive electrode. Colour scheme: yellow, pink and grey for [C${}_{2}$mim]${}^{+}$ ions, [NTf${}_{2}$]${}^{-}$ ions and graphene electrode. In (**a**), the regions shown in (**b**,**c**) are marked with green and blue rectangles, respectively.

All-atom molecular dynamics (MD) simulations were carried out using the OPLS force field for the [C${}_{2}$mim][NTf${}_{2}$] and [N${}_{4,1,1,1}$][NTf${}_{2}$] ILs [19,20,21]. The Lennard-Jones parameters for the carbon atoms of the graphene electrode ($\sigma_{LJ}=$ 3.4 Å and $\varepsilon_{LJ}=$ 0.086 kcal mol ${}^{-1}$) were taken from the literature [22] and have been tested in many published studies, e.g., [15]. The electrode atoms were fixed in space. The structures of the ions and the partial charges on the atoms are shown in Figure 2, and the ions were flexible. We generated the initial structures of the IL ions using the AVOGADRO software package [23]. Following this, 400 IL ion-pairs (400 anions and 400 cations) were randomly placed in a cubic simulation cell between the graphene electrodes with dimensions of 41.76 Å × 42.54 Å located on both ends of the simulation cell (Figure 3a). We added a vacuum region of 20 Å on both sides of the simulation cell beyond the fixed electrodes, and the system was periodic in the other directions. The IL ions were placed in the simulation cell such that they did not overlap with the electrodes. The distance between two electrodes (the z-direction) was adjusted so that the density of the IL was equal to that obtained in bulk IL simulations, as presented below. We found a distance of ∼10 nm and ∼11 nm reproduces the equilibrium density of [C${}_{2}$mim][NTf${}_{2}$] and [N${}_{4,1,1,1}$][NTf${}_{2}$] ILs, respectively. A cut-off radius for the Lennard-Jones and Coulombic interactions of 12 Å was used. The long-range Coulombic interactions were determined using the particle-particle-particle-mesh (PPPM) solver [24] with a slab geometry for the supercapacitor calculations imposed using the Yeh–Berkowitz condition to truly capture the 2D periodic boundary conditions [25]. We report properties averaged over three independently generated samples.

We then used a simulated annealing (SA) procedure to ensure that the IL ions were mixed properly [26,27]. In this SA procedure, the samples were heated up and down between the target temperature, 294 K and 800 K via NVT-MD simulations. The system temperature was controlled via use of the Nosé–Hoover thermostat [28,29] with a time constant of 100 fs. The samples were first simulated at 294 K for 0.2 ns. Following this, they were heated to 800 K over a period of 0.2 ns and the samples were held at this temperature for further 1.0 ns. After this step, the samples were cooled to 294 K over 0.5 ns. Last, a 0.2 ns simulation in NVT ensemble was performed at 294 K, and the configuration at the end of each 1 ps was recorded for further analysis. These trajectories (in total, 200 trajectories per SA cycle) were then used to calculate the mass density and charge density distributions at 294 K to characterise the molecular-level structure in the system.

In the simulations of pure ILs, the temperature and pressure were controlled via a Nosé–Hoover thermostat [28,29] with a time constant of 100 fs and barostat [28,30] with a time constant of 1000 fs, respectively. The equations of motion were integrated using a 1 fs time-step throughout all simulations. All MD simulations were performed using the open-access LAMMPS [18] simulation software package (lammps.sandia.gov).

The size of our atomic-level models of supercapacitors are large compared to most similar systems in the literature in terms of both the number of atoms making up the graphene electrode and the number of ion-pairs for the electrolyte. To ensure that our results are reliable we also consider three independently generated samples for each system, averaging these to obtain representative results or comparing the results of the three samples to give an indication of the reliability of the results. We also monitored how the charge density developed on the electrode with time and how the density profiles evolved with time to ensure that appropriate simulation times were used to determine averages. This is particularly important for ILs that are even more viscous than those studied here, since they are likely to become kinetically trapped within the relatively short timescales of the simulations. The development of symmetric profiles at ΔΨ=0 was one indicator that the simulation times were sufficient.

### 2.2. Constant Potential Simulations

Once we achieved equilibration of the IL in the presence of graphene electrodes, we applied a potential difference across the simulation cell (i.e., the potential difference between two electrodes) using the CPM [12,13,14] implemented in LAMMPS [18]. The fluctuations in the charge of the carbon atoms mimic the surface polarisation of the electrode due to the changes in the ion composition/concentration in the vicinity of the electrode. Rather than point charges, the charges are assumed to be located with a Gaussian distribution centred on the atoms, and a Gaussian parameter of 19.79 nm${}^{-1}$ was used [14]. The partial atomic charges of the IL atoms are kept fixed throughout the constant potential simulations.

## 3. Results and Discussion

### 3.1. Bulk Properties of Ionic Liquids

Prior to testing our supercapacitor models under varying applied potentials, we tested the force field parameters on pure ILs to ensure that we reproduced the experimental density of the ILs [31,32]. We generated cubic simulation cells for pure ILs with 256 ions for each, carrying out the equilibration and simulated annealing (SA) procedures described above [27]. The evolution of the density of pure IL at 298 K and 1 atm was calculated and is reported in Figure S1. It was found to converge to values that are close to the experimental results [31,32]. The convergence time for [N${}_{4,1,1,1}$][NTf${}_{2}$] was much longer than that of [C${}_{2}$mim][NTf${}_{2}$] which is consistent with the different viscosities of these two ILs.

### 3.2. Equilibration of Supercapacitors

We equilibrated our samples using a SA procedure [33,34]. We terminated the SA procedure when the change in the normalised mass density distribution as a function of vertical distance from the electrode surfaces was negligible. Our results indicate that 10 SA cycles (each SA cycle last ∼1.9 ns) are sufficient to obtain equilibrated normalised mass density distribution for the [C${}_{2}$mim][NTf${}_{2}$] system, and 15 SA cycles were required for the [N${}_{4,1,1,1}$][NTf${}_{2}$] system. In Figures S2 and S3 of the Supplementary Information, we report the normalised mass density distribution as a function of the distance from the electrode, averaged over the last two SA cycles. The plots indicate quite symmetric mass density distributions across the system for the entire IL and for the constituent anion/cation, as required in a well-equilibrated system.

### 3.3. Charge/Discharge Dynamics of the Electrode

**Figure 4:**
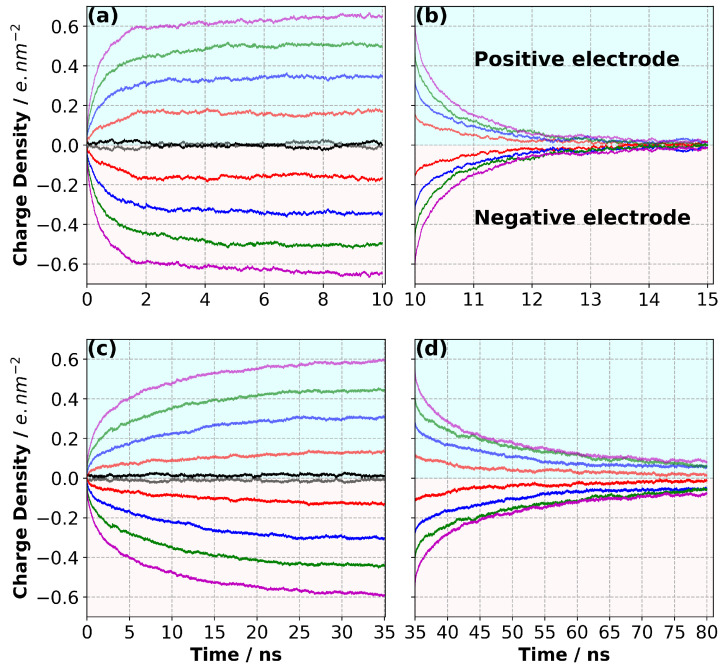
Evolution of the charge density on the electrodes during (**a**) charging with [C${}_{2}$mim][NTf${}_{2}$], (**b**) discharging with [C${}_{2}$mim][NTf${}_{2}$], (**c**) charging with [N${}_{4,1,1,1}$][NTf${}_{2}$] and (**d**) discharging with [N${}_{4,1,1,1}$][NTf${}_{2}$]. The results calculated with different potential differences are shown as a function of the simulation time. Colour scheme: black, red, blue, green and magenta for ΔΨ = 0 V, 1 V, 2 V, 3 V and 4 V, respectively.

The equilibrated systems were used as the initial states to which we applied a potential difference (ΔΨ), ranging between 0 and 4 V. Figure 4 shows the charge density evolution on the negative and positive electrodes for the [C${}_{2}$mim][NTf${}_{2}$] and [N${}_{4,1,1,1}$][NTf${}_{2}$] systems at 294 K. Our results indicate that there is a strong dependence of the charge/discharge rates of the electrode on the cation pair used in the supercapacitor model. The charge density on both the negative and positive electrodes came to equilibrium much faster for [C${}_{2}$mim][NTf${}_{2}$] (∼6–7 ns during constant potential simulations) than the [N${}_{4,1,1,1}$][NTf${}_{2}$] (∼30 ns), and a corresponding difference in timescale was observed for the discharge. Interestingly, the charge/discharge rate did not change greatly with the applied potential.

Noh and Jung [15] performed similar simulations for the IL [C${}_{2}$mim][SCN] at 350 K and reported that the charge density on the electrode converged in ∼2 ns at all applied potential differences (up to ΔΨ = 4 V). The higher temperature used in their work increased the mobility of the ions and hence the charge density on the electrode converged faster. However, the viscosities of the ILs reflect the ion mobility at a given temperature, and will also strongly influence the rate of charge/discharge. The viscosity of [C${}_{2}$mim][SCN] is 21 mPa.s [35] whereas [C${}_{2}$mim][NTf${}_{2}$] has a higher viscosity of 32.06 mPa.s and [C${}_{2}$mim][NTf${}_{2}$] has a much higher viscosity of 112.90 mPa.s at 298 K. [17] The order in viscosities is consistent with the order in the time required for the electrode to become fully charged.

We considered the mechanism of the charging dynamics of the EDLC for [C${}_{2}$mim][NTf${}_{2}$] and [N${}_{4,1,1,1}$][NTf${}_{2}$]. The data were analysed by fitting exponential functions to the charge density curves for each ΔΨ, following Noh and Jung [15]. Firstly, we considered a stretched exponential curve,

(1) $$\sigma \left(t\right)=\sigma_{t=\infty }\left[1-exp\left(-{\left(t/\tau_{1}\right)}^{\beta }\right)\right]$$

where the $\sigma_{t=\infty }$ value represents the equilibrium value of the charge density at a given ΔΨ, while $\tau_{1}$ determines the relaxation time. The value of β gives an indication of the departure of the relaxation process of the supercapacitor from a simple exponential, with a value close to one, consistent with exponential relaxation. We found that the data were overfitted using this function, especially at low values of the applied potential. This was evident from the sensitivity of the fits to the initial conditions; however, a simple exponential with two parameters did not fit the data well. To reduce the problem, we fitted the data for an applied potential of 2 V using Equation (1) finding that β∼0.5, and then used a value of β=0.5 for all other fits. The results are given in Figure S4a and Table S1. Our findings are consistent with the results obtained by Noh and Jung [15] for [C${}_{2}$mim][SCN] and the departure of β from unity is indicative of a change in the capacitance of the electrode during the charging process [15]. In agreement with the dynamics of the ILs, a larger relaxation time was required for the [N${}_{4,1,1,1}$][NTf${}_{2}$] system than the [C${}_{2}$mim][NTf${}_{2}$] system (Figure S4b), but the relaxation time did not vary greatly with ΔΨ. We note that the magnitude of charge density as a function time is the same for the anode and cathode, and the differences in the values of the coefficients are an indication of the reliability of the fit.

We also fit the data using a double exponential,

(2) $$\sigma \left(t\right)=\sigma_{t=\infty }\left[1-c\;exp\left(-t/\tau_{2}\right)-\left(1-c\right)\;exp\left(-t/\tau_{3}\right)\right]$$

where $\tau_{2}$ and $\tau_{3}$ are fitting parameters that represent relaxation processes with different timescales and *c* is a fitting parameter reflecting the weights of the two processes. Again, the equation over-fitted the data with the coefficients having a strong dependency on each other and sensitivity to the initial values of the fit parameters. In this case, by careful selection of the initial parameters, we were able to obtain consistent results. The results are presented in Figure S4 and Table S1. The values of $\tau_{2}$ and $\tau_{3}$ differ for [C${}_{2}$mim][NTf${}_{2}$], indicating that there are two timescales for the relaxation. The relaxation time increased slightly with the magnitude of ΔΨ, but the contribution from the shorter relaxation time is greater when the magnitude of ΔΨ is higher (*c* is larger). The results for [N${}_{4,1,1,1}$][NTf${}_{2}$] also suggest that there are two relaxation processes. In this case the relaxation time for the slower process decreases with ΔΨ, whereas there is no regular change for the slower process. We also note that the contribution from the fast process is the greatest when the magnitude of ΔΨ is the highest. These combined effects suggest that for neither system is there a large change in the overall relaxation time, which is also evident from an inspection of the data. The errors in the fitting parameters prevent further conclusions from being made.

We also considered the discharge rate as a function of ΔΨ (Figure 4b,d). We fit the following equations to the data:

(3) $$\sigma \left(t\right)=\sigma_{t=\infty }\left[exp\left(-{\left(t/\tau_{1}\right)}^{\beta }\right)\right]$$

(4) $$\sigma \left(t\right)=\sigma_{t=\infty }\left[c\;exp\left(-t/\tau_{2}\right)+\left(1-c\right)\;exp\left(-t/\tau_{3}\right)\right]$$

where $\tau_{1}$, $\tau_{2}$ and $\tau_{3}$ are relaxation times, and *c* is a fitting parameter reflecting the weights of the two processes. The results are shown in Figure S5 and Table S2. The same issues with the equations overfitting the data were found, and a single exponential gave a reasonable fit at low potentials. However, at higher ΔΨ, this was not the case. When the sum of exponentials was used, the two relaxation times were quite different. We emphasise that due to the overfitting, we should be cautious in drawing any conclusions from the values of the fit parameters used. However, it is very clear from the data and the fits that a single exponential function was inadequate, especially for the charging process at high ΔΨ. Furthermore, there is a striking difference in charging/discharging times of the IL systems ([C${}_{2}$mim][NTf${}_{2}$], [N${}_{4,1,1,1}$][NTf${}_{2}$] and [C${}_{2}$mim][SCN] [15]) whereas the charge on the electrodes reaches a similar value in all cases. The results might indicate that the ILs could be used for applications requiring different charge/discharge rates. However, we note that these are all extremely fast, and this might not be reproduced experimentally, as noted by Noh and Jung [15].

### 3.4. Charge Density Distribution on the Electrodes

**Figure 5:**
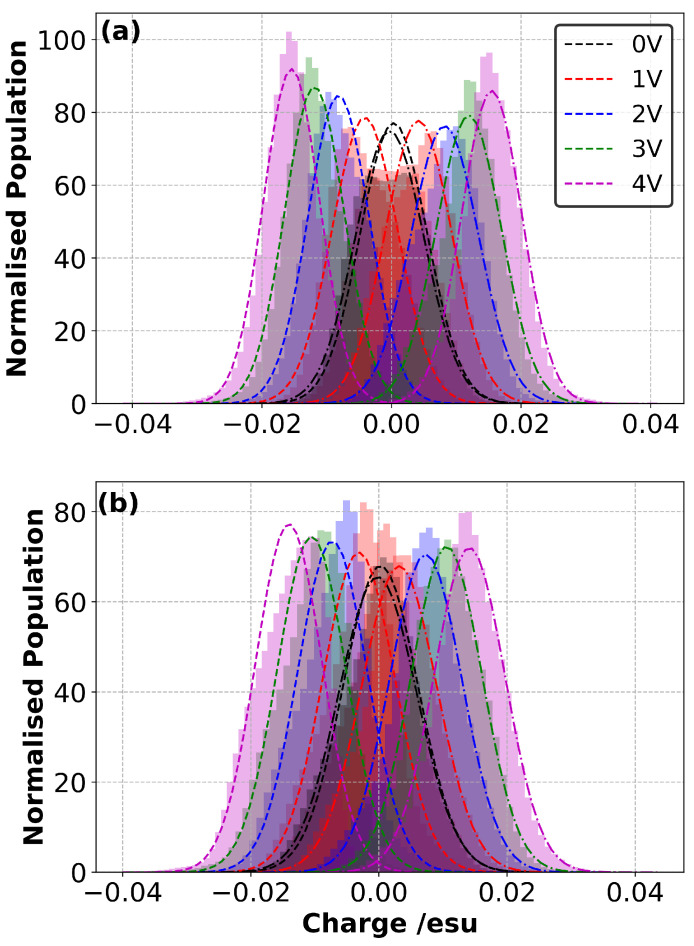
Histogram plot for the charge distribution of electrode ions in contact with the (**a**) [C${}_{2}$mim][NTf${}_{2}$] and (**b**) [N${}_{4,1,1,1}$][NTf${}_{2}$] for various ΔΨ values. Dashed lines and dashed–dotted lines represent the distribution of partial charges of the the negative electrode atoms and positive electrode atoms, respectively.

The distribution of charges on the carbon atoms of the electrodes can also be determined from the simulations. Figure 5, Figures S6 and S7 show the histogram of partial atomic charges on the carbon atoms of the negative and positive electrodes for both ILs (averaged over the last 0.5 ns of charging simulations and the three replicates of each sample, and therefore averaged over 1.5 ns in total for each system). The histograms are not Gaussian; however, as a visual guide, a Gaussian curve has been fitted to each histogram. As expected, there is a gradual shift in the mean of the histogram away from zero as ΔΨ increases.

Our results also indicate that, with an increase in ΔΨ, the amplitude of the histograms increases steadily for the [C${}_{2}$mim][NTf${}_{2}$] (Figure 5a and Figure S6) and they seem to become more Gaussian. This can be attributed to an increasing likelihood that there is a single type and orientation of ion on each electrode at higher voltages (i.e., cation on the negative electrode and anion on the positive electrode). At 0 V, both anions and cations are present in the first layer of molecules near the electrodes; however, as the voltage increases to 4 V, this layer is dominated by anions near the cathode and even more so by cations near the anode (this is discussed further below). Therefore, the extent of fluctuations in the partial atomic charges of the electrode atoms caused by the co-existence of anions and cations near each electrode became less pronounced with the increase in ΔΨ.

The change in the amplitude of the normalised population with applied voltage is less pronounced with the [N${}_{4,1,1,1}$][NTf${}_{2}$] electrolyte (Figure 5b and Figure S7) and the distribution on the anode remains non-Gaussian at high voltages. Due to the size and structure of the ions and their interaction with graphene, even at 0 V layering of the ions near the electrode is apparent, with the N of the [NTf${}_{2}$]${}^{-}$ able to approach the electrode more closely than the N of the [N${}_{4,1,1,1}$]${}^{+}$. The reordering of these layers (and therefore the presence of both types of ions, even at high voltages) could be the reason for the less pronounced change in amplitude. We also found that the distribution of partial charges on the negative electrode for the [N${}_{4,1,1,1}$][NTf${}_{2}$] IL was skewed irrespective of the value of ΔΨ. This is likely to be the result of a combination of factors associated with the distribution and orientation of the ions.

### 3.5. Charge Evolution in the Electrolyte

The charges on the electrodes reach steady values after ∼6–7 ns for [C${}_{2}$mim][NTf${}_{2}$] and ∼30 ns for [N${}_{4,1,1,1}$][NTf${}_{2}$], so it is of interest to see how this compares with the timescales for the redistribution of the mass and charge in the IL. To obtain an indication of this, we firstly calculated the current, $\Sigma_{i=1}^{N}v_{z,i}$, for [C${}_{2}$mim]${}^{+}$ and [NTf${}_{2}$]${}^{-}$ ions within a distance of 10 Å from each electrode, where N is the number of ions and $v_{z,i}$ is the velocity of the ion, *i*, in the direction perpendicular to the electrode. As shown in Figure S8, even with ΔΨ=4 V, the values fluctuate around zero, suggesting that there is no net movement of the ions from the bulk to/from this region. Fluctuations in the sum of velocities are much larger for the [C${}_{2}$mim]${}^{+}$ ions than for the [NTf${}_{2}$]${}^{-}$ ions, which reflect their differences in mass. There also seems to be a small difference in the amplitude of fluctuations near the two electrodes which could be attributed to the small difference in the number of ions in the regions (more positive ions near the negative electrode and more negative ions near the positive electrode); however, this does not change with time so any flux of ions must occur in the first few nanoseconds.

To explore this more carefully, we also considered the evolution of the change in bins at various distances from the electrodes and these are presented in Figures S9–S13. These were calculated from the partial charges of the atoms, including the carbon atoms of the electrode for ΔΨ=0, 1 and 4 V. For the region 0–5 Å there is a positive overall charge near both the positive and negative electrode and this quickly converges on the same timescale as the charge on the electrode, even for ΔΨ=4 V. The positive value is consistent with the strong interaction of the [C${}_{2}$mim]${}^{+}$ ion with the electrode surface. Interestingly, the change in the charge in this region seems to be due more to the movement of the [C${}_{2}$mim]${}^{+}$ rather than the [NTf${}_{2}$]${}^{-}$. Including regions beyond 5 Å from the electrode does not seem to contribute more to the time evolution, suggesting the main rearrangement occurs within the first 5 Å. Furthermore, the sum of all charges in the region 0–15 Å is zero at all times for all ΔΨ, indicating that the total charge of the IL in this region is equal to the charge on the electrode. This is what we expect for a wide pore and indicates good screening of the charges due to the EDL.

### 3.6. Normalised Mass Density

Using the results above, we presume that our samples are at equilibrium for the last 2 ns of the constant potential simulations, and therefore we considered the average, normalised mass density and charge density distributions in the electrolyte over the last 2 ns of the simulations. To define the mass density, we identify a reference point for each of the ions (N atom of [NTf${}_{2}$]${}^{-}$, N atom of [N${}_{4,1,1,1}$]${}^{+}$ and the C atom between the N atoms of [C${}_{2}$mim]${}^{+}$) (see Figure 2). The volume between the electrodes is divided into bins (0.2 Å) and the mass per bin is calculated (for individual reference atoms or all ions), and normalised based on the value of the mass density at the mid-point between the electrodes. All the results were obtained as averages over three independently generated samples.

**Figure 6:**
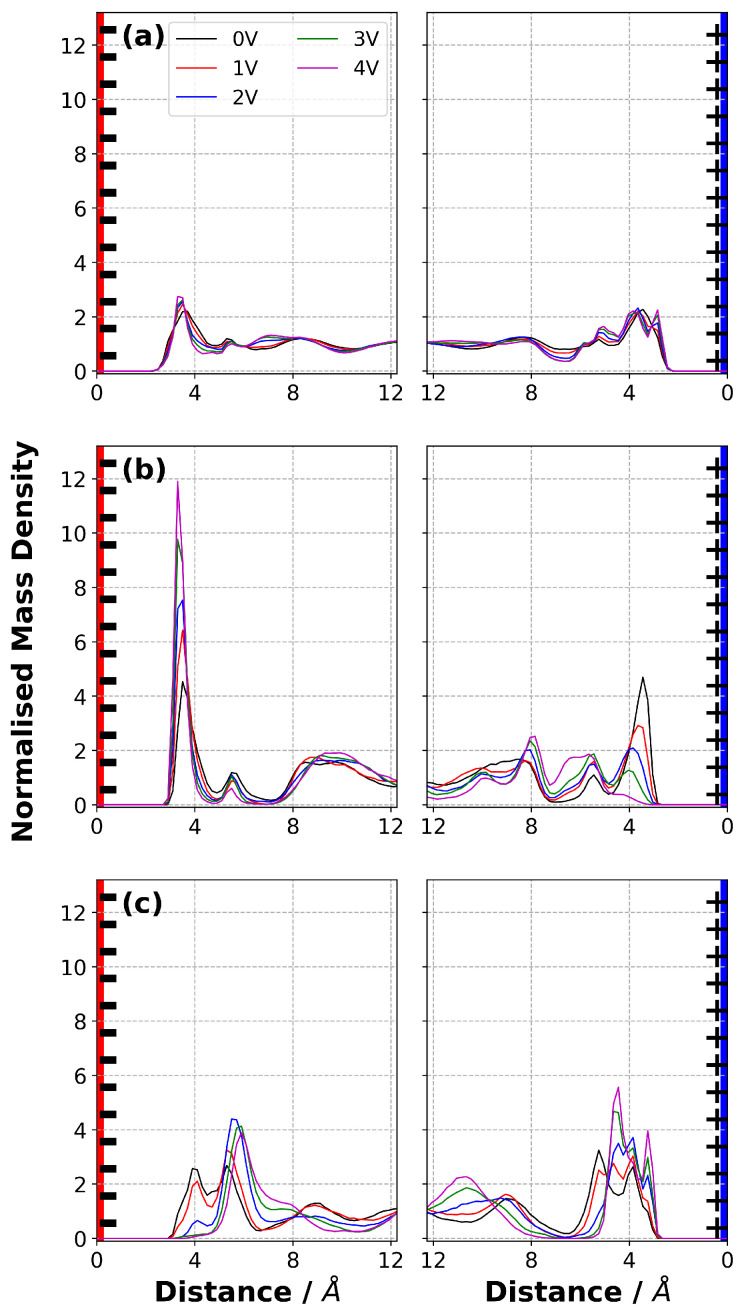
Average normalised mass density distribution of (**a**) [C${}_{2}$mim][NTf${}_{2}$], (**b**) [C${}_{2}$mim]${}^{+}$ and (**c**) [NTf${}_{2}$]${}^{-}$ at 294 K at various ΔΨ. The first and second columns represent the EDL in the vicinity of negative electrode and positive electrode, respectively.

We report the normalised mass density distribution of each ion in [C${}_{2}$mim][NTf${}_{2}$] and the entire IL in Figure 6. Our simulation results show that with a potential difference of 0 V applied using the CPM, the mass distribution in the ILs near both electrodes was quite symmetric. This validation is important to ensure that, when we start applying a potential difference, the system is equilibrated and all changes occurring in the EDL are driven by the applied potential. We then analysed the normalised mass density distribution with potential differences larger than 0 V.

Oscillatory mass density profiles were observed, even at ΔΨ = 0 V, indicating a layered structure of ions in the vicinity of electrode. The [C${}_{2}$mim]${}^{+}$ mass density in the first layer near the negative electrode increased with applied potential (Figure 6b) as the Coulombic interactions between the negatively charged electrode and [C${}_{2}$mim]${}^{+}$ ions became stronger. As well as an increase in the height of the peak, a slight shift towards the anode was observed. On the other hand, the [C${}_{2}$mim]${}^{+}$ mass density decreased with ΔΨ on the positive electrode (e.g., the first peak) and it almost disappeared when ΔΨ = 4 V, due to repulsive Coulombic interactions. A similar observation can be made for the appearance of a more distinct first [NTf${}_{2}$]${}^{-}$ peak in the EDL on the positive electrode. The amplitude of this peak tends to increase with ΔΨ, suggesting more [NTf${}_{2}$]${}^{-}$ ions were attracted towards the positive electrode. The peak due to [NTf${}_{2}$]${}^{-}$ ions on the anode completely disappeared as the voltage was increased. It is important to note that the total normalised mass density shows relatively little variation with potential. This suggests that main effect of applying a potential is that the ions swap places or reorient, and this will be discussed further below.

Notably, the change in amplitude with ΔΨ of the first and second peak for the [C${}_{2}$mim]${}^{+}$ ions on the negative electrode shows reverse trends. This is due to the combination of the effect of applied potential difference and the arrangement of the [NTf${}_{2}$]${}^{-}$ ions. The position of the first peak of [NTf${}_{2}$]${}^{-}$ on the negative electrode shifted away from the electrode and the intensity decreased with the increase in ΔΨ, being no longer evident for ΔΨ larger than 2 V. However, the change in the [NTf${}_{2}$]${}^{-}$ peak located at ∼ 5–6 Å from the negative electrode indicated that more [NTf${}_{2}$]${}^{-}$ ions are located in this region as ΔΨ increases. The second [C${}_{2}$mim]${}^{+}$ peak is also located at ∼ 5–6 Å and it decreased with ΔΨ. This shows that the composition in this region changes even though the total density does not change greatly. Similarly, near the positive electrode the [NTf${}_{2}$]${}^{-}$ ions move towards the electrode whereas the [C${}_{2}$mim]${}^{+}$ ions are gradually expelled from the first layer as ΔΨ increases, and the overall normalised mass density distribution does not change greatly.

**Figure 7:**
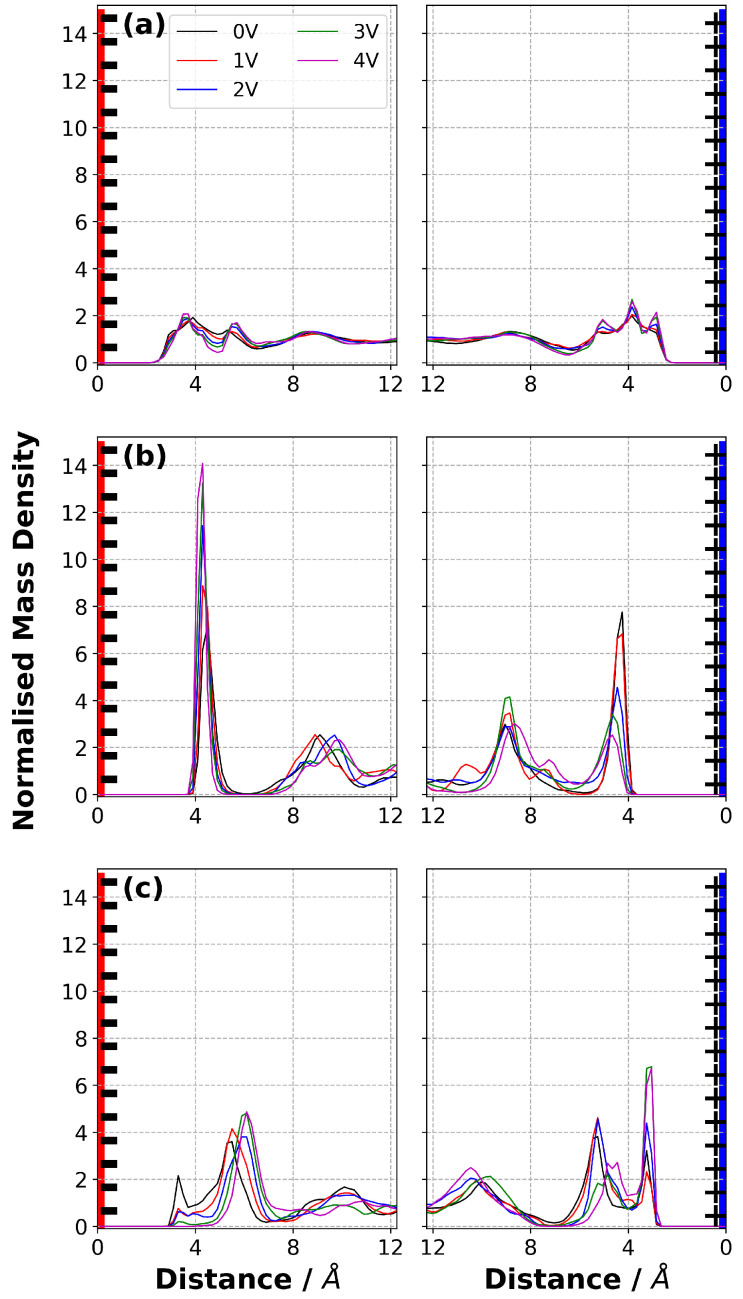
Average normalised mass density distribution of (**a**) [N${}_{4,1,1,1}$][NTf${}_{2}$], (**b**) [N${}_{4,1,1,1}$]${}^{+}$ and (**c**) [NTf${}_{2}$]${}^{-}$ at 294 K at various ΔΨ. The first and second columns represent the EDL in the vicinity of negative electrode and positive electrode, respectively.

Unlike in [C${}_{2}$mim][NTf${}_{2}$], the first anion peak is closer to the electrode than the first cation peak in [N${}_{4,1,1,1}$][NTf${}_{2}$] when ΔΨ=0. In addition, the second cation peak is much further away from the electrode. This is consistent with the relative sizes of the cations. Otherwise, this system shows similar trends to [C${}_{2}$mim][NTf${}_{2}$], as shown in Figure 7, where the increase in ΔΨ caused more [N${}_{4,1,1,1}$]${}^{+}$ ions to be adsorbed onto the negative electrode, whereas more [NTf${}_{2}$]${}^{-}$ ions were accumulated near the positive electrode. An increase in ΔΨ shifted the location of first peak of [N${}_{4,1,1,1}$]${}^{+}$ near the positive electrode towards the bulk. Unlike the [NTf${}_{2}$]${}^{-}$ in [C${}_{2}$mim][NTf${}_{2}$], a sharp first peak for [NTf${}_{2}$]${}^{-}$ was observed on the positive electrode (Figure 7c) at all ΔΨ. This indicates that the orientation of [C${}_{2}$mim]${}^{+}$ on the positive electrode restricted the access of the [NTf${}_{2}$]${}^{-}$ to the electrode surface, but [N${}_{4,1,1,1}$]${}^{+}$ did not. This also indicates that it is necessary to consider the relative orientation of ions in each IL to properly capture the molecular-level picture of EDLs (see Orientation of Ions in Electric Double Layer, below).

### 3.7. Charge Density Distribution in Electrolyte

**Figure 8:**
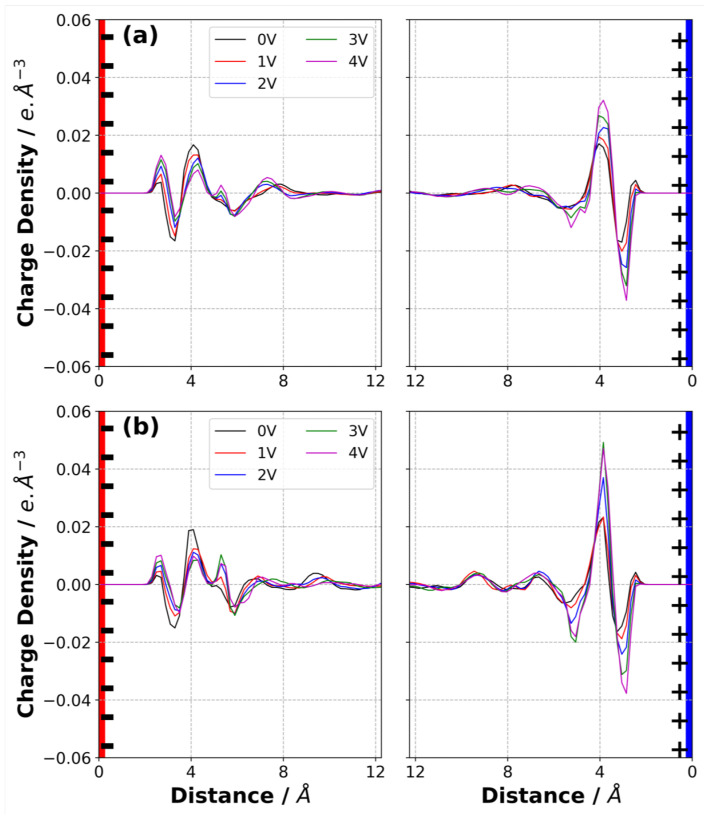
Charge density distribution of (**a**) [C${}_{2}$mim][NTf${}_{2}$] and (**b**) [N${}_{4,1,1,1}$][NTf${}_{2}$] at 294 K at various potential difference applied across the simulation cell. The first and second columns represent the EDL in the vicinity of negative electrode and positive electrode, respectively.

In addition to the normalised mass density profiles, the charge density distribution in the electrolyte as a function of normal distance from the electrode surfaces was calculated for both [C${}_{2}$mim][NTf${}_{2}$] and [N${}_{4,1,1,1}$][NTf${}_{2}$], and are reported in Figure 8. Perhaps surprisingly, given the different nature of the cations, the charge density profiles for the two ILs are in many ways quite similar.

For both ILs at 0 V, there is a charge density profile due to the packing of the anions and cations and their interactions with the graphene electrode and this results in a positive, then negative, peak in the charge density distribution as a function of the distance from the electrode. As the potential increases the co-ion (negative ion near negative electrode and positive ion near positive electrode) is displaced from this layer and replaced by counter-ions. This results in both these peaks becoming more positive near the anode, and more negative near the cathode. The third (positive) peak shows the opposite behaviour, with the co-ions accumulating here on application of a potential. Therefore, the third peak becomes more negative near the anode and more positive near the cathode as ΔΨ increases.

It is interesting to note that the peak closest to the electrodes for both ILs is positive for all ΔΨ≤3 V. This was also observed by Noh and Jung [15] in their study of [C${}_{2}$mim][SCN]. It was proposed to be due to the strong π−π stacking interaction between the the [C${}_{2}$mim]${}^{+}$ ion and graphene electrodes, but we also observe it with [N${}_{4,1,1,1}$]${}^{+}$. It is clear from the mass density plots that there is some [C${}_{2}$mim]${}^{+}$ close to the cathode, even at 3 V. However, we propose that the positive peak could also be partly due to the penetration of the weakly charged alkyl groups of the cations, with their positively charged hydrogen atoms, into the layer of [NTf${}_{2}$]${}^{-}$ ions on the cathode.

Although the overall charge density profiles for the two ILs are quite similar, the arrangement of the ions differs. Considering [C${}_{2}$mim][NTf${}_{2}$], our results show that, according to our mass density plots, the ratio of the [C${}_{2}$mim]${}^{+}$ to [NTf${}_{2}$]${}^{-}$ and ions near the negative electrode is larger than the ratio of the [NTf${}_{2}$]${}^{-}$ to [C${}_{2}$mim]${}^{+}$ ions near the positive electrode, as shown in Figure 6b,c and Figure 7b,c.

This difference can be explained by the shape of the ions and their interaction with the electrode. To quantify this, we calculated the [C${}_{2}$mim]${}^{+}$/[NTf${}_{2}$]${}^{-}$ and [NTf${}_{2}$]${}^{-}$/[C${}_{2}$mim]${}^{+}$ ratios (in number of ions) within a distance of 5 Å from each electrode as a function of ΔΨ (which corresponds to the end of the first peak in Figure 6b). Our results indicate that the [C${}_{2}$mim]${}^{+}$/[NTf${}_{2}$]${}^{-}$ ratio on the negative electrode was 12.29 ± 1.54 while the [NTf${}_{2}$]${}^{-}$/[C${}_{2}$mim]${}^{+}$ ratio on the positive electrode was 2.40 ± 1.28 at ΔΨ = 2 V, calculated over the last 1 ns of simulation (averaged over three independently generated samples). Increasing the potential difference resulted in a greater increase in the [C${}_{2}$mim]${}^{+}$/[NTf${}_{2}$]${}^{-}$ ratio on the negative electrode than the [NTf${}_{2}$]${}^{-}$/[C${}_{2}$mim]${}^{+}$ ratio on the positive electrode. The increase in both ratios (i.e., [C${}_{2}$mim]${}^{+}$/[NTf${}_{2}$]${}^{-}$ and [NTf${}_{2}$]${}^{-}$/[C${}_{2}$mim]${}^{+}$) highlights the interplay between overscreening (formation of single layers of counterions) and crowding (formation of multiple layers of co-ions) [36]. With the increase in ΔΨ, crowding becomes more pronounced. Moreover, we found that the average [C${}_{2}$mim]${}^{+}$/[NTf${}_{2}$]${}^{-}$ ratio (averaged over both electrodes and three independently generated samples) was 1.36 ± 0.66 at ΔΨ = 0 V. The favoured adsorption of [C${}_{2}$mim]${}^{+}$ ions on the electrode surface could be ascribed to the interactions between the aromatic ring of the [C${}_{2}$mim]${}^{+}$ ions and the graphitic surface of the electrode, even in the absence of applied potential. Experimental results (X-ray reflectivity signals) have shown such density enhancement at the uncharged graphene electrode [37].

We also point out that the mass density distributions were calculated based on reference points within the ions. In Figure 3b,c we show the ions with any atom within 4 Å of the electrode and see clearly that, despite using the reference points, the density of the [C${}_{2}$mim]${}^{+}$ is higher on the negative electrode; this is partly because the [C${}_{2}$mim]${}^{+}$ lies flat on the surface and therefore the reference point is closer to the electrode. In contrast, parts of many [NTf${}_{2}$]${}^{-}$ ions are within that range, even though there are few with the reference point within it. Similarly on the positive electrode, since the [NTf${}_{2}$]${}^{-}$ adopts many orientations, there are more with one or more atom within 4 Å than with the reference point within that range.

**Figure 9:**
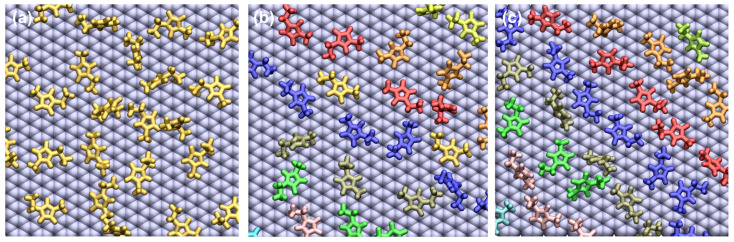
A snapshot of [C${}_{2}$mim]${}^{+}$ ions with any atom within 4 Å from the electrode surface at (**a**) $\Psi_{-}$ = 0 V, (**b**) $\Psi_{-}$ = −0.5 V and (**c**) $\Psi_{-}$ = −2 V. Each colour in (**b**,**c**) represents a different stripe.

Our simulation results also indicate that the [C${}_{2}$mim]${}^{+}$ ions form a pattern on the negative electrode. The formation of stripes of [C${}_{2}$mim]${}^{+}$ ions on the negative electrode became more pronounced with applied potential difference (Figure 9). In a computational study, Montes-Campos et al. investigated the pattern formation of butyl-3-methylimidazolium tetrafluoroborate [Bmim][BF${}_{4}$] on a graphene electrode and reported the striped patterns formed in the IL near the electrode [38]. These authors used a fixed charge method and assigned partial charges of ±1 e nm${}^{-2}$ to the carbon atoms of the graphene electrode (where the maximum charge density we obtained in our simulations was ∼0.6 e nm${}^{-2}$ when ΔΨ = 4 V). Similar pattern formation in ILs in the vicinity of the electrode was observed in experiments [39,40]. For example, Su et al. reported the formation of stripes of [Bmim][BF${}_{4}$] at varying ΔΨ [39]. Several parameters such as vacancies in the electrode and the presence of water can also trigger the formation of patterns near the electrode surface [38]. In our work, we considered a defect-free fully graphitic and water-free electrode surface.

**Figure 10:**
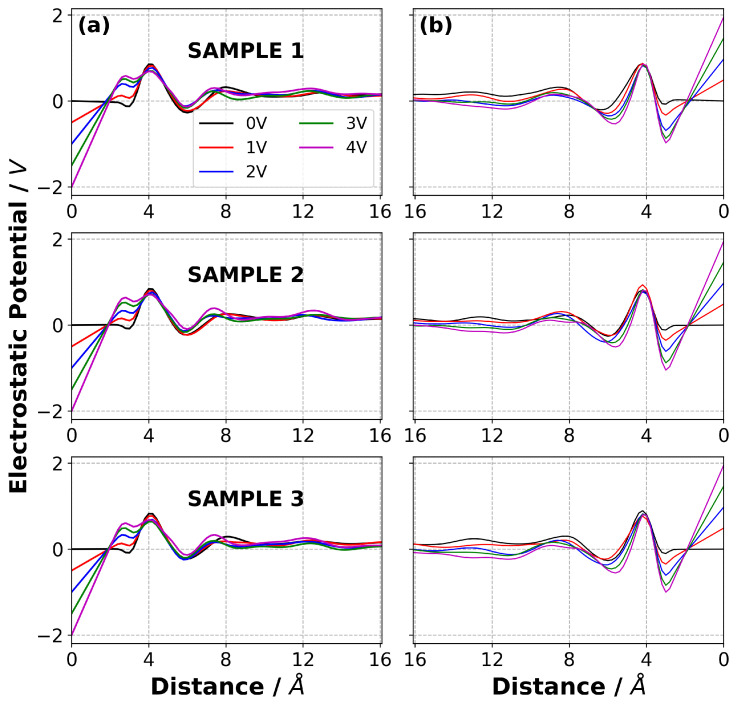
Electrostatic potential profile as a function of ΔΨ for [C${}_{2}$mim][NTf${}_{2}$] system on the (**a**) negative electrode and (**b**) positive electrode for each sample at 294 K. The distance of zero corresponds to the electrode surface. The three samples were simulated under identical conditions but with different initial configurations. They give an indication of the robustness of the results.

**Figure 11:**
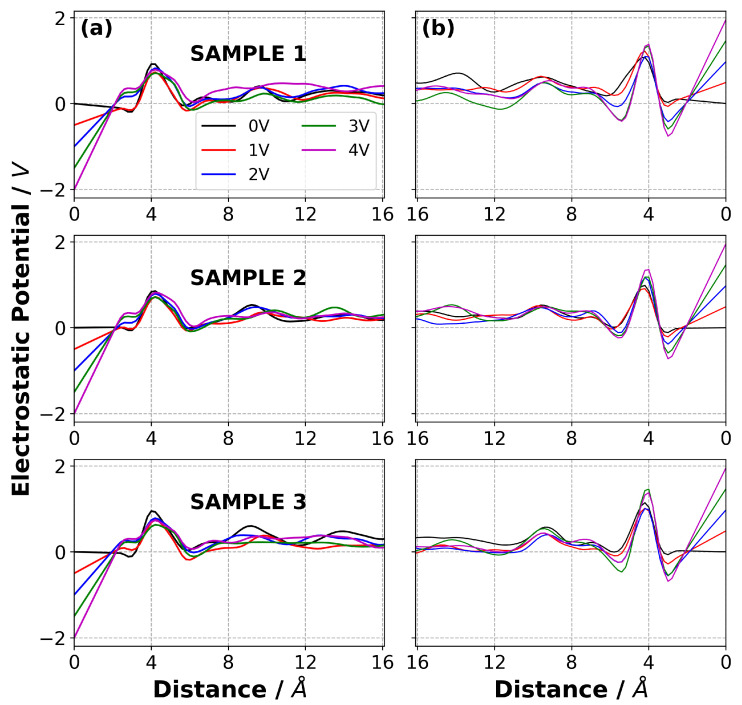
Electrostatic potential profile as a function of ΔΨ for [N${}_{4,1,1,1}$][NTf${}_{2}$] system on the (**a**) negative electrode and (**b**) positive electrode, for each sample at 294 K. The distance of zero corresponds to the electrode surface. The three samples were simulated under identical conditions but with different initial configurations. They give an indication of the robustness of the results.

We also calculated the electrostatic potential, Φ, across the EDL for each IL as a function of ΔΨ, and the results are shown in Figure 10 and Figure 11. Details of the calculation of the electrostatic potential from the solution of the Poisson equation are given in the Supplementary Information. The dependence of Φ on the distance from the electrode flattens once the distance from the electrode is greater than ∼12 Å because of the screening of the charge on the electrodes due to the EDL. Our results show that the electrostatic potential in the bulk is small and decreases with ΔΨ. The small non-zero electrostatic potential that is evident even with ΔΨ=0 is indicative of the layering of the cations and anions on the surface due to different shapes and interactions between the ions and the electrodes.

**Figure 12:**
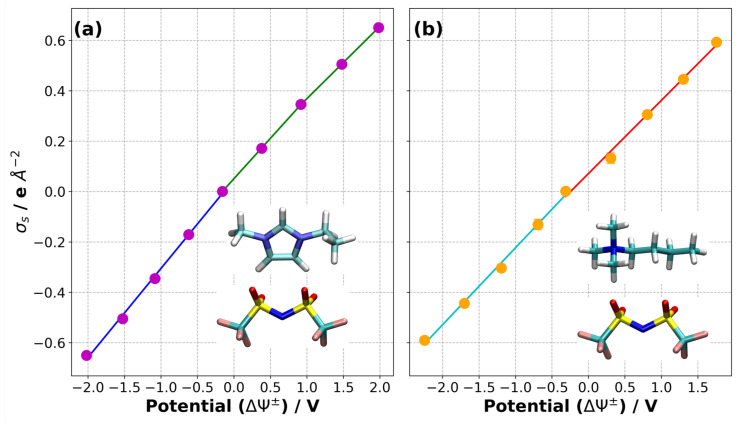
Surface charge density versus potential ($\Delta \Psi^{+/-}$) plots for the calculation of differential capacitance for (**a**) [C${}_{2}$mim][NTf${}_{2}$] and (**b**) [N${}_{4,1,1,1}$][NTf${}_{2}$] system. Lines with different colours correspond to a slope used to calculate differential capacitance.

Using the calculated electrostatic potential profiles and the average charges on the electrodes, we calculated the differential capacitance of the supercapacitor:

(5) $$C_{diff}=\frac{\partial \sigma_{s}}{\partial \Delta \Psi^{+/-}}$$

where $\sigma_{s}$ is the surface charge density on the electrode, and $\Delta \Psi^{+/-}=\left(\Psi^{+/-}-\Phi_{bulk}\right)$ where $\Phi_{bulk}$ is the electrostatic potential in the centre of the supercapacitor (calculated by averaging the value in the central region, more distant than 30 Å from either electrode). We calculated the capacitance from the slopes in Figure 12 and found that $C_{diff}^{-}=5.66$ and $C_{diff}^{+}=4.60$μ F/cm${}^{2}$ for the [C${}_{2}$mim][NTf${}_{2}$] system and $C_{diff}^{-}=4.90$ and $C_{diff}^{+}=4.66$μ F/cm${}^{2}$ for the [N${}_{4,1,1,1}$][NTf${}_{2}$] system. Our results indicate that the capacitance is slightly larger on the negative electrode than that on the positive electrode for both IL–electrode systems. A comparison of the two IL systems indicates that the capacitance on the negative electrode was higher for the [C${}_{2}$mim][NTf${}_{2}$] system than the [N${}_{4,1,1,1}$][NTf${}_{2}$] system. On the other hand, the capacitance on the positive electrode was almost the same for the two IL systems which is consistent with the use of the same anion in both ILs. These results are similar to those obtained using different cations and anions in previous studies [15,41]. However, we did not attempt to determine $C_{diff}$ as a function of $\Delta \Psi^{+/-}$ as it was clear from the comparison of replicates in Figure 10 and Figure 11 that the numerical errors could be significant. We note that the differences in the capacitance are quite small, given the very large difference in the dynamics of the ions.

### 3.8. Differences in Residency Time for Ions in the EDL

Different ion types will spend different times in the EDL due to various factors such as the strength of non-bonded interactions between the same/different ion types and interactions with the electrode surface. To determine the probability that ions remain in the EDL, we tagged the ions that were found in the EDL when the constant potential was applied to the system and traced them over time (10 ns for [C${}_{2}$mim][NTf${}_{2}$] and 35 ns for [N${}_{4,1,1,1}$][NTf${}_{2}$]), calculating the proportion of these ions that stayed in various regions of the EDL. We refer to these as the life probability of the ions in each region. The results are reported for each ion and both ILs in Figure S14. We divided the EDL into regions: 0–5 Å and 5–10 Å for the [C${}_{2}$mim][NTf${}_{2}$] system, and 0–6 Å and 6–10 Å for the [N${}_{4,1,1,1}$][NTf${}_{2}$] IL. We based this division on the mass density distribution of a cation of each ion type, as shown in Figure 6 and Figure 7. We also analysed the ion life probability in the entire double layer, from 0 to 10 Å for both ILs.

When we considered the region 0–10 Å from the electrode surface, we found that both [C${}_{2}$mim]${}^{+}$ and [NTf${}_{2}$]${}^{-}$ ions moved from this region into the bulk with different probabilities. The life probability of [C${}_{2}$mim]${}^{+}$ ions on the negative electrode was over 0.7 irrespective of ΔΨ and tended to slightly increase with ΔΨ. This indicates that the [C${}_{2}$mim]${}^{+}$ ions tend to stay in the EDL on the negative electrode with the increase in the charge of the electrode surface, due to Coulombic interactions. Conversely, a decreasing trend of the life probability of [C${}_{2}$mim]${}^{+}$ ions on the positive electrode was observed due to Coulombic interactions. Interestingly, the life probability of [NTf${}_{2}$]${}^{-}$ ions in the 10 Å-thick layer on both electrodes was always over 0.8 and was not significantly affected by ΔΨ. Overall the probability of exchange between the 0–10 Å region and the bulk is about 0.2 over the simulation periods and this does not change greatly with the applied potential, ion or IL.

When we break down the 10 Å-thick EDL layer into two separate layers from 0–5 Å and 5–10 Å, we find distinct differences. Our calculations indicate that there is a decrease in the number of [C${}_{2}$mim]${}^{+}$ ions that move out the first layer near the negative electrode as ΔΨ increases. The increased charge on negative electrode causes the [C${}_{2}$mim]${}^{+}$ ions to be strongly bound to the surface and limits their movement away from this region. On the other hand, the proportion of [C${}_{2}$mim]${}^{+}$ ions that remain in this region near the positive electrode almost linearly decreases with the increase in ΔΨ, suggesting a fast repulsion of [C${}_{2}$mim]${}^{+}$ ions from the positive electrode surface. When we perform the same analysis for the [NTf${}_{2}$]${}^{-}$ ions in the [C${}_{2}$mim][NTf${}_{2}$], we find that the life probability of [NTf${}_{2}$]${}^{-}$ on the negative electrode decreased with ΔΨ. At a potential difference of 3 V or higher, almost all [NTf${}_{2}$]${}^{-}$ ions diffused away from the first layer towards the second layer. On the other hand, similar to the [C${}_{2}$mim]${}^{+}$ ions on the negative electrode, the [NTf${}_{2}$]${}^{-}$ ions on the positive electrode possessed increased life probability in the first layer due to the favourable interactions between the negatively charged [NTf${}_{2}$]${}^{-}$ ions and positive electrode.

The life probabilities of both ion types showed substantially different behaviours in the second layer. Our analysis show that the exchange of ions in the second layer (that is, between 5–10 Å from the electrode surface for the [C${}_{2}$mim][NTf${}_{2}$]) was larger compared to that found in the first layer (that is, between 0–5 Å from the electrode surface). This is partly because they could move into the first layer or the bulk region. Overall, the ions that were found in the second layer had a higher mobility, even when no potential was applied. The life probability of [C${}_{2}$mim]${}^{+}$ ions was under 0.5 in the second layer on the negative electrode, while this was always above 0.7 in the first layer irrespective of ΔΨ. Moreover, in the second layer, more [C${}_{2}$mim]${}^{+}$ ions stayed close to the positive electrode than the negative electrode when ΔΨ≥ 2.

Since [N${}_{4,1,1,1}$]${}^{+}$ ion is bulkier than [C${}_{2}$mim]${}^{+}$, the reference atom (i.e., the nitrogen atom) can not easily approach the electrode surface. Our simulation results indicate that the first peak for the [N${}_{4,1,1,1}$]${}^{+}$ ion ended around 6 Å from the electrode (Figure 7b). Therefore, the first layer for the [N${}_{4,1,1,1}$][NTf${}_{2}$] IL was chosen to be 6 Å, while the second layer was the region between 6 to 10 Å from the electrode surfaces. Our analysis showed similar trends for the [N${}_{4,1,1,1}$][NTf${}_{2}$] IL as were observed for [C${}_{2}$mim][NTf${}_{2}$] (Figure S15). The life probability of ions in [N${}_{4,1,1,1}$][NTf${}_{2}$] in the EDL was larger compared with the ions in [C${}_{2}$mim][NTf${}_{2}$] IL. This suggests that the ions in [N${}_{4,1,1,1}$][NTf${}_{2}$] were less likely to move out of the layers they were found in compared to [C${}_{2}$mim][NTf${}_{2}$] over the time periods considered, which were longer for [N${}_{4,1,1,1}$][NTf${}_{2}$]. This can be linked to the difference in the viscosity and mobility of the ILs.

### 3.9. Orientation of Ions in the Electric Double Layer

The value of ΔΨ has an influence on the orientation of ions with respect to the electrode surfaces. To better capture and quantify this, we calculated the angular distribution of ions on both electrodes as a function of ΔΨ. The reference angle for each ion type, θ, is explained in Figure S16 of the Supplementary Information. We divided the EDL in several separate layers and calculated the angular distribution in each layer for a given ΔΨ.

**Figure 13:**
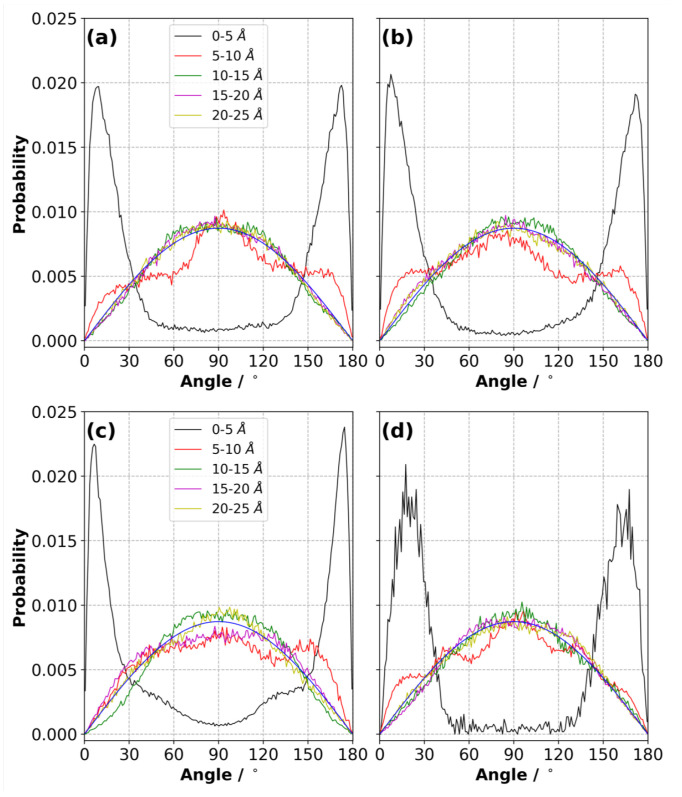
Angle distribution in the layers (up to 20 Å from each electrode) for the [C${}_{2}$mim]${}^{+}$ ions in the [C${}_{2}$mim][NTf${}_{2}$] IL for (**a**) $\Psi_{-}$ = 0 V, (**b**) $\Psi_{+}$ = 0 V, (**c**) $\Psi_{-}$ = 2 V and (**d**) $\Psi_{+}$ = 2 V at 294 K (averaged over three independently generated samples). The blue curve shows the distribution expected in a completely isotropic system.

Figure 13 shows the probability distribution of the orientation of the [C${}_{2}$mim]${}^{+}$ ions in the [C${}_{2}$mim][NTf${}_{2}$] IL (Figures S17–S28) near the positive and negative electrodes for ΔΨ= 0 and 4 V, with the blue line showing the expected distribution in an isotropic medium. At 0 V, the plots should be symmetric at around $\theta =90^{\circ }$ and Figure 13a,b should be the same. Departures from this give an indication of the statistical error. Our simulation results indicate that in the first layer when the ΔΨ = 0 V, the [C${}_{2}$mim]${}^{+}$ ions are much more likely to have an orientation with $0^{\circ }\leq \theta \leq 30^{\circ }$ or $150^{\circ }\leq \theta \leq 180^{\circ }$ than in an isotropic medium, indicating a configuration where the aromatic ring of [C${}_{2}$mim]${}^{+}$ lies nearly parallel to the electrode (a value of 0${}^{\circ }$ and 180${}^{\circ }$ corresponds to a perfect parallel alignment, while a value of 90${}^{\circ }$ for θ corresponds to a perpendicular configuration) (Figure S16). This preference slightly changes with ΔΨ. On the negative electrode (Figure 13a–c), the peak position shifted with the increase in potential, indicating a more parallel configuration. In addition, new shoulders at ∼45${}^{\circ }$ and ∼135${}^{\circ }$ emerged at ΔΨ = 4 V, suggesting steric hindrance is forcing some [C${}_{2}$mim]${}^{+}$ ions to adopt a configuration that maximises the number of [C${}_{2}$mim]${}^{+}$ ions in the first layer (up to 5 Å). In the layers further than 5 Å from the electrode surface, the configurational preference of [C${}_{2}$mim]${}^{+}$ was reduced, and a bulk-like distribution was adopted for distances greater than 10 Å.

The orientation of [C${}_{2}$mim]${}^{+}$ showed a similar trend on the positive electrode. Noisier distribution curves were obtained for the first layer on the $\Psi_{+}=2$ V electrode because fewer [C${}_{2}$mim]${}^{+}$ ions were found in this region due to repulsion between the [C${}_{2}$mim]${}^{+}$ cations and positive electrode. Unlike the distribution on the negative electrode, no new shoulders around ∼45${}^{\circ }$ and ∼135${}^{\circ }$ were observed and the peak slightly shifted away from the parallel configurations.

**Figure 14:**
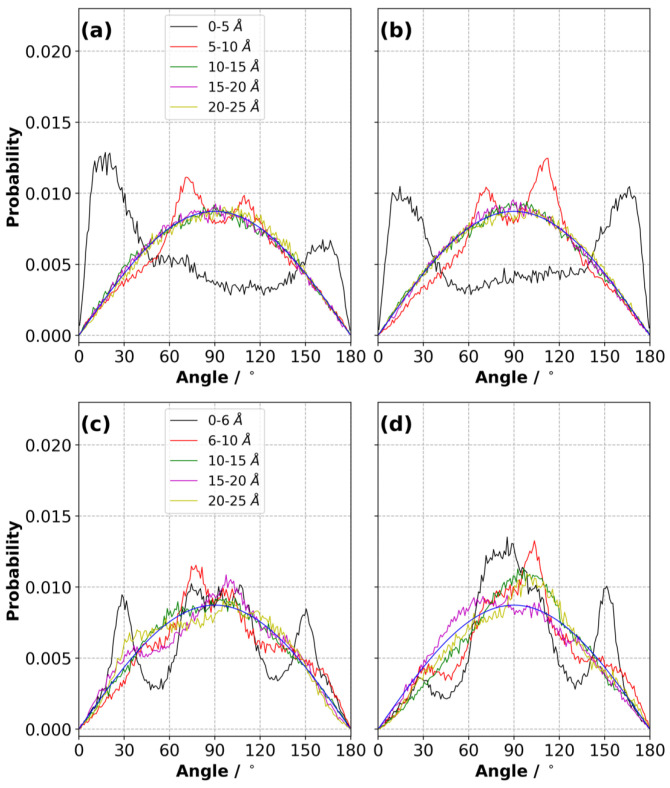
Angle distribution of [NTf${}_{2}$]${}^{-}$ ions in the layers (up to 20 Å from each electrode) for (**a**) $\Psi_{-}$ = 0 V, (**b**) $\Psi_{+}$ = 0 V in the [C${}_{2}$mim][NTf${}_{2}$] IL, for (**c**) $\Psi_{-}$ = 0 V, (**d**) $\Psi_{+}$ = 0 V in the [N${}_{4,1,1,1}$][NTf${}_{2}$] IL at 294 K (averaged over three independently generated samples).

Figure 14 shows the angle probability distribution of [NTf${}_{2}$]${}^{-}$ ions in the first layer at ΔΨ = 0 V for both IL systems. Due to statistical error, asymmetry and differences in the plots in Figure 14a,b or c,d are observed, yet there are some noticeable trends. The presence of the electrode created a preferential configuration for [NTf${}_{2}$]${}^{-}$ in the first layer (i.e., up to 5 and 6 Å for [C${}_{2}$mim][NTf${}_{2}$] and [N${}_{4,1,1,1}$][NTf${}_{2}$] systems, respectively), even when no potential was applied. These angles varied depending on the IL. For example, [NTf${}_{2}$]${}^{-}$ ions had a greater likelihood around 15${}^{\circ }$ and 165${}^{\circ }$ than in an isotropic medium in the [C${}_{2}$mim][NTf${}_{2}$] IL (Figure 14a,b). This preference became less pronounced on the positive electrode with the increase in ΔΨ (Figures S17–S21). When ΔΨ≥ 3 V, the orientation of [NTf${}_{2}$]${}^{-}$ ions on the positive electrode became similar to that in the bulk. This can be explained by steric crowding. The increasing number of [NTf${}_{2}$]${}^{-}$ on the positive electrode cancelled out the preferential distribution of [NTf${}_{2}$]${}^{-}$ without applied potential, causing a bulk-like angle distribution. In contrast, the [NTf${}_{2}$]${}^{-}$ ions are more isotropically distributed in [N${}_{4,1,1,1}$][NTf${}_{2}$] (Figure 14c,d). Moving away from the electrode surfaces caused peaks at angles of ∼30${}^{\circ }$, ∼80${}^{\circ }$, ∼100${}^{\circ }$ and ∼150${}^{\circ }$ to disappear as [NTf${}_{2}$]${}^{-}$ ions adopted bulk-like distributions.

**Figure 15:**
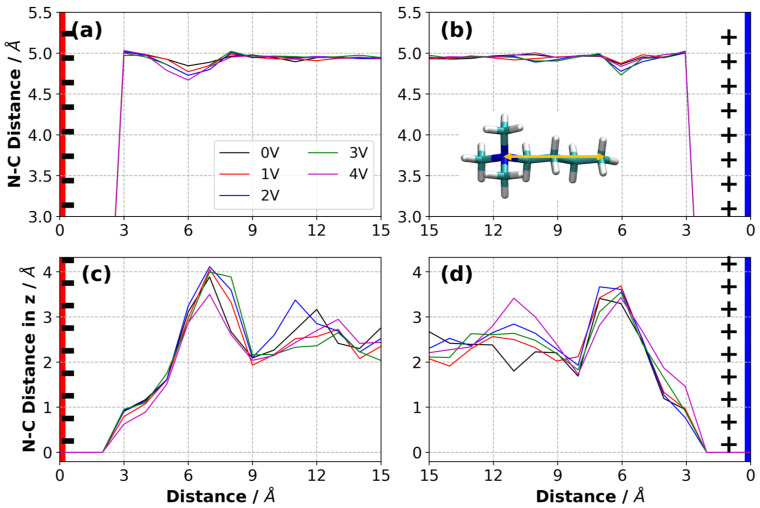
Distribution of the nitrogen-to-distal carbon distance in [N${}_{4,1,1,1}$]${}^{+}$ ions calculated in all three dimensions on the (**a**) negative electrode, (**b**) positive electrode, and in the normal direction to the electrode surface (the z-direction) on the (**c**) negative electrode and (**d**) positive electrode for each ΔΨ at 294 K (averaged over three independently generated samples). The yellow arrow represents the distance between nitrogen and carbon atom.

Using the vectors defined in Figure S16, the orientation of [N${}_{4,1,1,1}$]${}^{+}$ is hard to quantify. The orientation of this vector resembles that in the bulk at all ΔΨ (Figures S29–S40). To examine if the charged graphene surface affects the extension of [N${}_{4,1,1,1}$]${}^{+}$ ions in the vicinity of electrodes, we calculated the distribution of nitrogen-to-distal carbon distance (NC distance, or distance from N3 to CT distance, see Figure 2) for each ΔΨ. Figure 15a,b shows that the NC distance in [N${}_{4,1,1,1}$]${}^{+}$ ions is insensitive to the distance from the electrode and ΔΨ, but adopt a slightly shorter NC distance in the region where the concentration of [N${}_{4,1,1,1}$]${}^{+}$ is lower, such as ∼6 Å (Figure 7). In the bulk, the distribution of the NC distance fluctuated around 4.95 Å, where the ideal distance between the nitrogen atom and distal carbon atom (i.e., the distance in a geometrically optimised ion in a vacuum) in an [N${}_{4,1,1,1}$]${}^{+}$ ion was ∼5.2 Å. We also considered the NC distance only in the normal direction with respect to the electrode surface (i.e., the z-direction). This would have a value of zero when the vector from N3 to CT is parallel to the surface, and ∼5 Å when it is perpendicular. We found that the largest NC distance in the z-direction appeared to be ∼4 Å at a distance of 6–7 Å from the electrode surfaces, as shown in Figure 15c,d. The bulk value of the NC distance in the z-direction fluctuated around 2.1–2.9 Å for the applied ΔΨ. The extended NC distance at a distance of 6–7 Å from the surface, where the concentration of [N${}_{4,1,1,1}$]${}^{+}$ is at its minimum, indicates a perpendicular-like configuration of [N${}_{4,1,1,1}$]${}^{+}$ ions in that region. On the other hand, in the vicinity of the electrode surface, the [N${}_{4,1,1,1}$]${}^{+}$ ions adopted a parallel-like configuration (Figure S41).

### 3.10. Diffusion of Ions in EDL

Ions can have different mobilities in different layers of the EDL due to varying concentrations of each layer and their normal distance from the electrode surface. It can also be expected that the interactions between an electrified electrode surface and ions have influence on the mobility of ions. To address the issue of the mobility of ions, we generated the mean square displacement (MSD) curves of each ion type.

**Figure 16:**
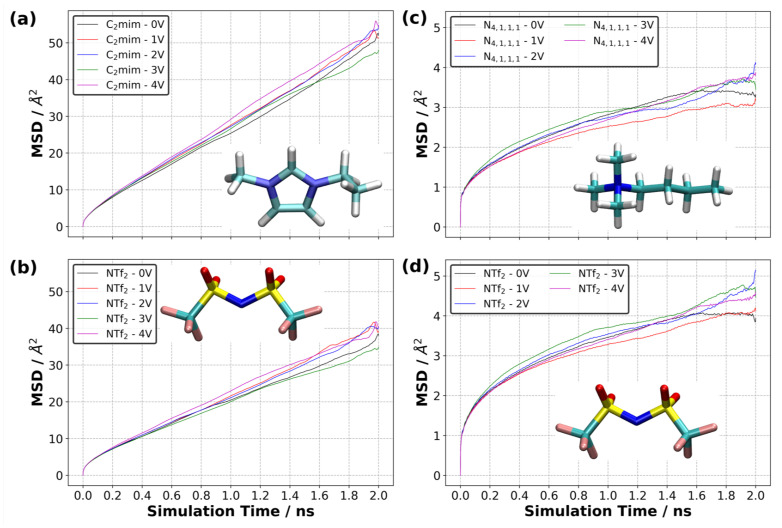
Calculated mean square displacement (MSD) at various potential difference at 294 K for (**a**) [C${}_{2}$mim]${}^{+}$ and (**b**) [NTf${}_{2}$]${}^{-}$ in [C${}_{2}$mim][NTf${}_{2}$] system, (**c**) [N${}_{4,1,1,1}$]${}^{+}$ and (**d**) [NTf${}_{2}$]${}^{-}$ in [N${}_{4,1,1,1}$][NTf${}_{2}$] system (averaged over three independently generated samples).

**Figure 17:**
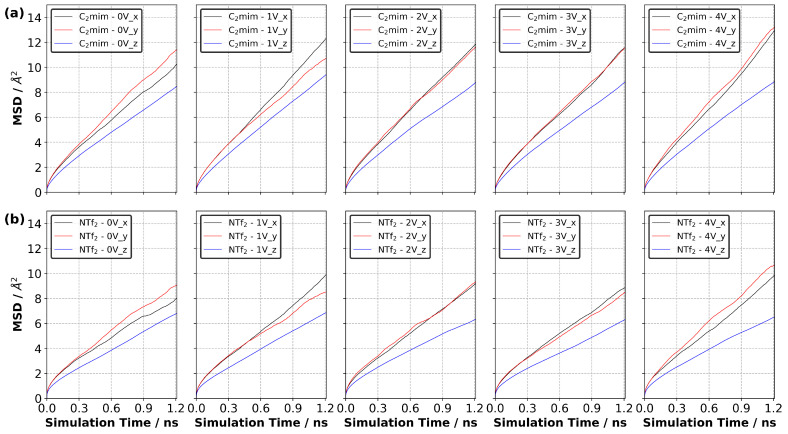
Calculated mean square displacement (MSD) at 294 K in each principal direction at each ΔΨ value for (**a**) [C${}_{2}$mim]${}^{+}$ and (**b**) [NTf${}_{2}$]${}^{-}$ (averaged over three independently generated samples).

Our simulation results indicate that ΔΨ did not strongly affect the MSD of each ion type in the supercapacitor cell, as shown in Figure 16. We calculated diffusion coefficients for each ion type in [C${}_{2}$mim][NTf${}_{2}$] only, as the MSD of [N${}_{4,1,1,1}$][NTf${}_{2}$] did not reach the diffusive regime within the 2 ns simulation period. Since ΔΨ only seemed to be affecting the overall MSDs slightly, we only calculated the diffusion coefficients at 0 V and 4 V. We found that the diffusion coefficient of [C${}_{2}$mim]${}^{+}$ was 4.15 and 4.88 × 10${}^{-11}$ m${}^{2}$ s${}^{-1}$ at 0 V and 4 V, respectively. [NTf${}_{2}$]${}^{-}$ had slightly lower diffusion coefficients, 2.85 and 3.47 × 10${}^{-11}$ m${}^{2}$ s${}^{-1}$ at 0 V and 4 V, respectively. The experimental value of the diffusion coefficient of bulk IL was reported as 4.0 × 10${}^{-10}$ m${}^{2}$ s${}^{-1}$[42]. When we compare our predicted diffusion coefficients to those measured in bulk IL, our results look almost one order of magnitude smaller. This could be for many reasons. First, we have solid surfaces (i.e., the graphene electrodes), which reduce the mobility of ions, particularly in the normal direction, to the electrode surfaces. Second, the finite-size of the simulation cell could affect the ultimate value of the diffusion coefficient. In addition, the diffusion coefficient is sensitive to the force field and density of the sample. For example, the absolute values of overall charge that each ion carries can influence the mobility of ions in the electrolyte. Tuning the partial charges of ions may enhance the mobility of ions in the electrolyte. This can also alter the final value of the charge density of the electrode, as reported in Figure 4. Therefore, a careful investigation is needed to fully capture the ion charge effect on the system dynamics. Considering the first issue, we calculated the diffusion coefficient of [C${}_{2}$mim][NTf${}_{2}$] in each principal direction at ΔΨ of 0 and 4 V. Our results indicated the lowest MSD is found in the normal direction (i.e., the z-direction) to the electrode surface (Figure 17, Figures S42 and S43), giving one-dimensional diffusion coefficients of 3.96, 4.59 and 3.45 × 10${}^{-11}$ m${}^{2}$ s${}^{-1}$, and 4.29, 5.16 and 3.09 × 10${}^{-11}$ m${}^{2}$ s${}^{-1}$ in the x-, y- and z-directions for [C${}_{2}$mim]${}^{+}$ at 0 and 4 V, respectively. Although these differ, it is not an order of magnitude difference and indicates that the diffusion in all directions is lower than the experimental bulk result.

Considering the second issue, Simonnin et al. [43] investigated the effect of simulation cell size on the predicted diffusion coefficients along a solid surface with a Lennard-Jones fluid, where no surface polarisation occurs. These authors reported a monotonic increase in the diffusion coefficient with the height-to-length (H/L) ratio of the simulation cell. Simonnin et al. [43] concluded that when the ratio of H/L is close to approximately 2.8, the finite size effects on diffusion coefficients were minimised. In our case, the value of the H/L ratio is ∼2.4, suggesting that minimised finite size effects on our predicted diffusion coefficients might occur. Recently, Park et al. [44] used a polarisable force field for both [Bmim][BF${}_{4}$] and [Bmim][PF${}_{6}$] with graphene electrodes, and found a dramatic effect on the predicted diffusion coefficients. A maximum of diffusion coefficient for [BF${}_{4}$]${}^{-}$ in [Bmim][BF${}_{4}$] was predicted for a simulation cell length (i.e., the electrode-to-electrode distance) of around 4 nm. However, the non-monotonic behaviour of the diffusion coefficients as a function of electrode-to-electrode distance could be due to a statistical error.

In this work, we also studied the response of ions to an applied potential in a supercapacitor model where we set the electrode-to-electrode distance to ∼10 nm and ∼11 nm for [C${}_{2}$mim][NTf${}_{2}$] and [N${}_{4,1,1,1}$][NTf${}_{2}$] systems, respectively. This ensured the presence of a bulk region, which separated two EDLs. He et al. [45] investigated how the length of the electrode-to-electrode distance influenced the diffusion of ions of [C${}_{2}$mim][NTf${}_{2}$], confined between two graphene electrodes. They varied the distance between 0.72 and 1.1 nm. In the smaller systems, the normalised diffusion coefficient (D${}_{ion}$/D${}_{bulk}$) of [C${}_{2}$mim]${}^{+}$ increased with charge density on the electrode surface, while a decreasing trend for [NTf${}_{2}$]${}^{-}$ was observed with the increase in charge density. Since the ions were confined in a very narrow region, the ions may not be able to rotate or translate freely. This difference in the mobilities of ions can also be attributed to the shape, size and total charge carried by the ion, and the charge density on the electrode. In our simulations, the electrode-to-electrode distance was sufficiently large so that the density was fairly uniform, except in a relatively small portion of the system. Therefore, little variation was observed with ΔΨ.

Our MSD curves reported in Figure 16 show that the mobility of each ion type in the [N${}_{4,1,1,1}$][NTf${}_{2}$] system is much lower than that in the [C${}_{2}$mim][NTf${}_{2}$] system and that [NTf${}_{2}$]${}^{-}$ is much lower in [N${}_{4,1,1,1}$][NTf${}_{2}$] than in [C${}_{2}$mim][NTf${}_{2}$]. In addition, the MSD of [NTf${}_{2}$]${}^{-}$ increases more quickly than that of [N${}_{4,1,1,1}$]${}^{+}$ in [N${}_{4,1,1,1}$][NTf${}_{2}$], whereas the MSD of [NTf${}_{2}$]${}^{-}$ increases more slowly than that of [C${}_{2}$mim]${}^{+}$ in the [C${}_{2}$mim][NTf${}_{2}$]. This shows that the interactions between the ions strongly affect their mobility in the IL.

**Figure 18:**
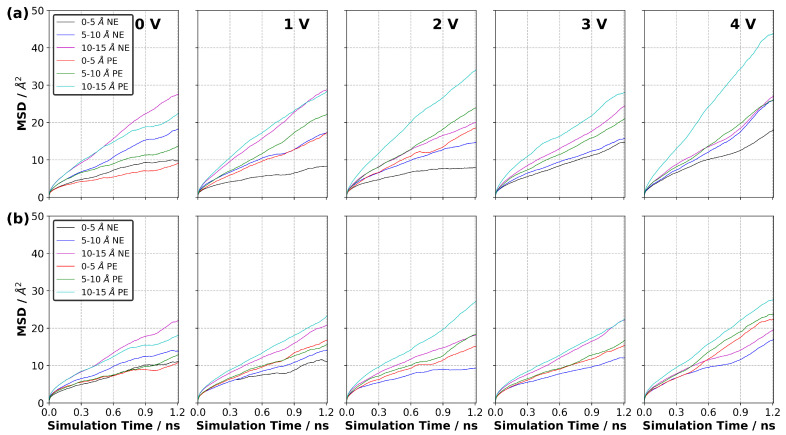
Calculated mean square displacement (MSD) in the first layer (0–5 Å), second layer (5–10 Å) and third layer (10–15 Å) from the electrode surface at each ΔΨ for (**a**) [C${}_{2}$mim]${}^{+}$ and (**b**) [NTf${}_{2}$]${}^{-}$ in the [C${}_{2}$mim][NTf${}_{2}$] system at 294 K. NE and PE stand for negative electrode and positive electrode, respectively (averaged over three independently generated samples).

We also calculated a local MSD in the EDL only (Figures S44–S47). The local MSD was defined such that the MSD in the first layer (0–5 Å) was calculated by only considering the ions that are found in the first layer at each calculation step or time *t*. This means that the ions do not have to be in the first layer at time t=0 (or prior to the calculation of MSD). If the ions are found in the first layer at the calculation step, then they are considered in the MSD calculations. Figure 18 shows the MSD curves for [C (or prior to the calculation of MSD). If the ions are found in the first layer at the calculation step, then they are considered in the MSD calculations. Figure 18 shows the MSD curves for [C${}_{2}$mim]${}^{+}$ and [NTf${}_{2}$]${}^{-}$ for each value of ΔΨ. Our results indicate that the MSD of the ions increases more quickly with time in the layers far from the electrode surfaces. When ΔΨ=0 V, the MSD curves for both ions in the first layer (0–5 Å) and second layer (5–10 Å) are generally lower than those in the third layer (i.e., the distance between 10 to 15 Å from the electrode surface). Even the charge-neutral electrode surface constrains the mobility of ions in the first two layers due to the hard matter–soft matter interactions in the electrode-to-electrolyte interface. When we compared the mobility of ions in the second layer (5–10 Å) to that in the third layer (10–15 Å), we found that the proximity of the electrode continued to cause the ions to move more slowly in the second layer irrespective of ion type. Another observation was that the mobility of cations ([C${}_{2}$mim]${}^{+}$) near the positive electrode was larger than that near the negative electrode at ΔΨ (larger than 0 V). This increased mobility can be attributed to the unfavourable electrostatic interactions between the cations and positively charged electrodes. A similar conclusion can be made for the mobility of anions found near the negative electrodes. Our results also show that the net flux of each ion type during the charging process is zero (Figure S48).

## 4. Conclusions

We investigated the dynamics of two ionic liquids with different viscosities as electrolytes in a supercapacitor model where flat graphene was used as the electrode and the temperature was 294 K. A constant potential difference was applied across the supercapacitor model to observe the behaviour of ions in response to electrode surface polarisation. The partial atomic charges of electrode atoms, which fluctuated during the constant potential simulations, caused ions in the EDL to readjust their configurations. Our simulations revealed that the ring of the [C${}_{2}$mim]${}^{+}$ tended to adopt a near-parallel configuration with respect to the negative electrode surface and this tendency increased with the increase in ΔΨ. We showed that [C${}_{2}$mim]${}^{+}$ ions formed almost 45${}^{\circ }$ stripes on the negative electrode, and this became more pronounced with the increase in ΔΨ. The analysis of the distribution of nitrogen-to-distal carbon distance in [N${}_{4,1,1,1}$]${}^{+}$ ions showed that the interactions with the electrode surface also caused the [N${}_{4,1,1,1}$]${}^{+}$ ions to arrange so that the NC vector preferred to be parallel to the surface. Overall, the MSD curves showed that [C${}_{2}$mim]${}^{+}$ diffused slightly faster than [NTf${}_{2}$]${}^{-}$ in [C${}_{2}$mim][NTf${}_{2}$] at all ΔΨ studied here. On the other hand, the anion ([NTf${}_{2}$]${}^{-}$) diffused slightly faster than the cation ([N${}_{4,1,1,1}$]${}^{+}$) in the [N${}_{4,1,1,1}$][NTf${}_{2}$] system. In addition, the mobility of ions in the [N${}_{4,1,1,1}$][NTf${}_{2}$] IL was almost one order of magnitude smaller than the ions in the [C${}_{2}$mim][NTf${}_{2}$] system. This can be attributed to the difference in the viscosities of these ILs.

Overall, the results showed significant differences in the double layer structure and orientation of the molecules with a change in the cation and the applied potential. The dynamics were also significantly affected by the cation, which will influence the power density of the supercapacitor; however, it did not change greatly with the applied potential. Moreover, the overall charge distribution was not strongly influenced by the cation or potential and hence, there, the capacitance remained similar for the systems considered.

On charging, the ions rearranged and reoriented within a region of ∼1 nm from the electrode until a steady charge on the electrode and steady charge density profile developed. This occurred on timescales of O(10 ns) for the ionic liquids considered since it did not rely on the diffusion of ions from large distances. The timescale increased with the viscosity of the fluid.

Our computational procedure will enable us to capture the molecular-level interactions between ionic liquid and electrode materials in supercapacitors to assist in obtaining a better performance (i.e., increase in capacitance and mobility of ions). In addition, our computational procedure can be exploited to screen numerous ionic liquids as electrolytes, and the outcomes of simulations could be input to a machine learning pipeline to explore pure or mixed ionic liquids for the ultimate optimised performance.

## Supplementary Materials

The following are available online at https://www.mdpi.com/2079-4991/10/11/2181/s1.

## Abbreviations

The following abbreviations are used in this manuscript:

EDLSC	Electric double-layer supercapacitor
EDL	Electric double layer
IL	Ionic liquid
MD	Molecular dynamics
CPM	Constant potential method
FCM	Fixed charge method
SA	Simulated annealing
MSD	Mean square displacement
