# Supplementary material for: Investigation of the Ionic Liquid Graphene Electric Double Layer in Supercapacitors Using Constant Potential Simulations
Source: Nanomaterials (Basel). 2020 Nov 1;10(11):2181. doi: 10.3390/nano10112181 (PMC7693729; doi:10.3390/nano10112181)
Supplement: Supplementary file 1 [file nanomaterials-10-02181-s001.pdf]

**SUPPORTING INFORMATION**

# Investigation of the Ionic Liquid-Graphene Electric Double Layer in Supercapacitors using Constant Potential Simulations

Baris Demir[a], Debra J. Searles[a,b]

[a]Centre for Theoretical and Computational Molecular Science, The Australian Institute for Bioengineering and Nanotechnology, The University of Queensland, Queensland 4072, Australia;

[b]School of Chemistry and Molecular Biosciences, The University of Queensland, Brisbane, Queensland 4072, Australia;

E-mail: b.demir@uq.edu.au
E-mail: d.bernhardt@uq.edu.au

## CONTENT

**1.** Bulk ionic liquid (Figure S1)
**2.** Normalised mass density distributions in the supercapacitor (Figures S2-S3)
**3.** Charging/Discharging Dynamics (Figures S4-S5, Tables S1-S2)
**4.** Charge Distribution on Electrodes (Figures S6-S7)
**5.** Charge evolution (Figures S8-S13)
**6.** Life probability of ions in the electric double layer (Figures S14-S15)
**7.** Angle distribution analyses (Figures S16-S41)
**8.** Mean square displacement curves (Figures S42-S47)
**9.** Flux evolution (Figure S48)
**10.** Voltage drop across a supercapacitor.

**1. Bulk Ionic Liquid**

We randomly placed 256 anions and cations for each IL system in a cubic simulation cell using PACKMOL [Martínez L., Andrade R., Birgin E.G. and Martínez J.M., J. Comput. Chem., **30** (2009), 2157-2164]. We then equilibrated the samples in the NPT-MD ensemble at 294 K and 1 atm over a period of 10-30 ns. After this step, we applied a simulated annealing procedure to the samples so that the mixing of the ions was ensured. The details of this simulated annealing procedure were reported in the main text.

The system temperature and pressure were controlled using the Nosé-Hoover thermostat and barostat, respectively [Nosé, S., J. Chem. Phys., **81** (1984), 511-519, Hoover W.G., Phys. Rev. A, **31** (1985), 1695-1697] . The Lennard-Jones and Coulombic interactions were cut off at an interatomic distance of 12 Å and long-range Coulombic interactions were calculated using a particle-particle-particle-mesh (PPPM).

**Figure S1** shows the density evolution of each IL system at 294 K and 1 atm.

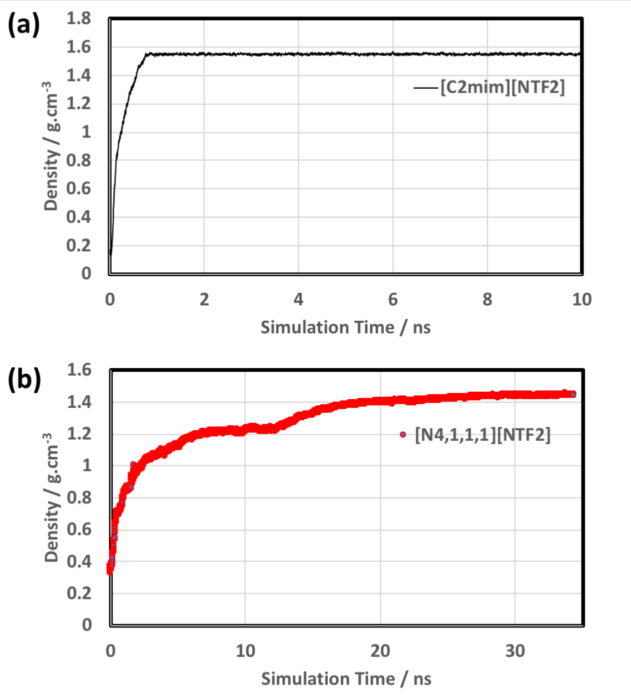


**Figure S1.** Density plots of the $[C_2mim][NTf_2]$ and $[N_{4,1,1,1}][NTf_2]$ ionic liquids at 294 K. Note that the experimental densities are 1.51 g $cm^{-3}$ for $[C_2mim][NTf_2]$ at 302.68 K and 1.39 g $cm^{-3}$ for $[N_{4,1,1,1}][NTf_2]$ at 302.94 K [Jacquemin, J., Husson, P., Padua, A. A. H., Majer, V., Green Chem., **8** (2006), 172-180]

## 2. Normalised mass density distributions in the supercapacitor electrolyte

**Figure S2** shows the normalised mass density distribution for the $[C_2mim][NTf_2]$ system obtained during the last 2 ns part of the equilibration simulations at 294 K (no applied potential difference).

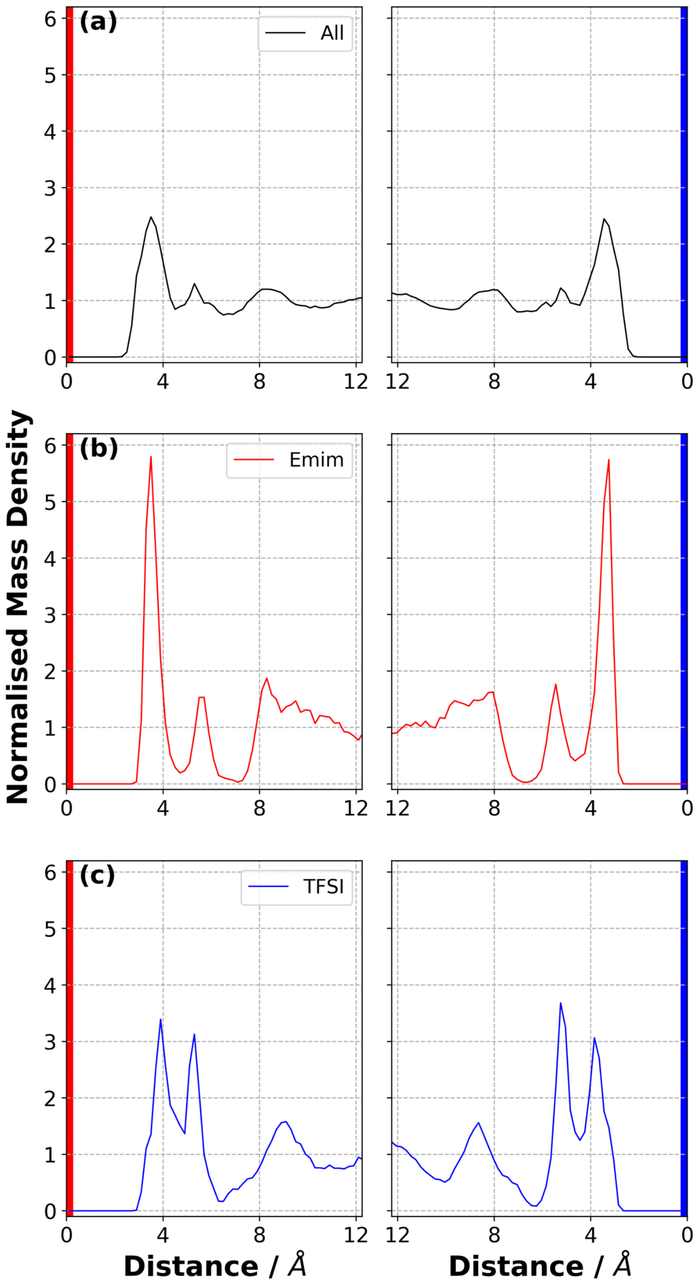


**Figure S2**. Normalised mass density distribution for (a) the entire ionic liquid, (b) $[C_2mim]^+$ and $[NTf_2]^-$ ions. Normalisation is with respect to the density in the bulk region (more distant than 30 Å from either electrode).

**Figure S3** shows the normalised mass density distribution for the $[N_{4,1,1,1}][NTf_2]$ IL obtained during the last 2 ns part of the equilibration simulations at 294 K (no applied potential difference).

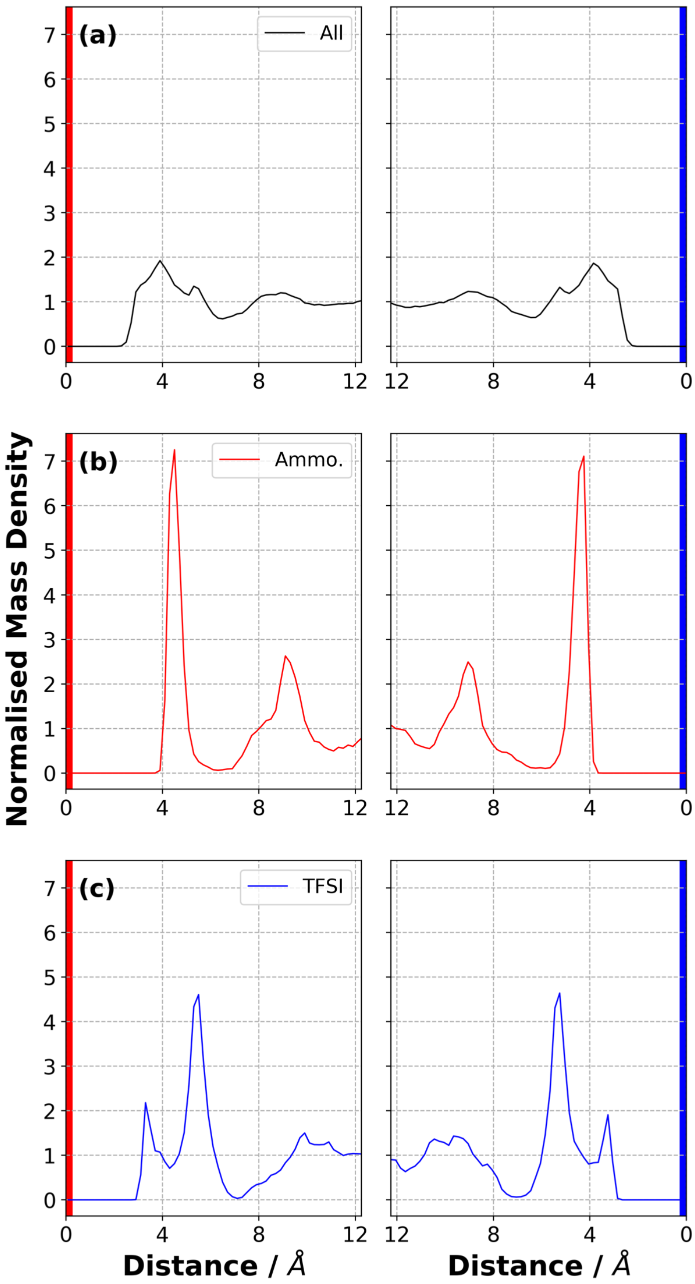


**Figure S3**. Normalised mass density distribution for (a) the entire ionic liquid, (b) $[N_{4,1,1,1}]^+$ and $[NTf_2]^-$ ions. Normalisation is with respect to the density in the bulk region (more distant than 30 Å from either electrode).

## 3. Charging/Discharging Dynamics

We fitted the charging/discharging dynamics of the supercapacitor (see **Figure 3** of the main text) using Eqns. 1 and 2 of the main text in the charge process and Eqns. 3 and 4 of the main text for the discharge process. The value of $\beta$ was fixed at 0.5 in Eqn. 1 based on fitting to $\Delta\Psi$ = 4 V.

The fitted curves and corresponding parameters for the charge process are shown in Figure S6 and Table 1. Those for the discharge process are shown in Figure S7 and Table 2.

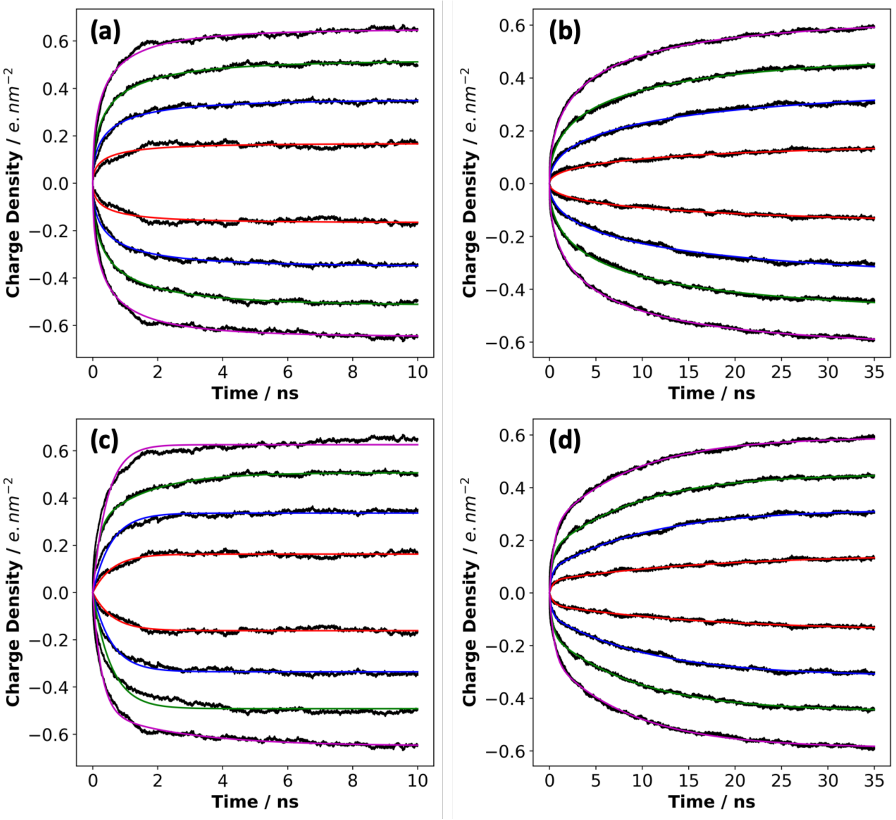


**Figure S4**. Charge density plots with fit curves for the charging process. Fitting of Eqn. 1 to (a) [$C_2$mim][$NTf_2$] data and (b) [$N_{4,1,1,1}$][$NTf_2$] data. Fitting of Eqn. 2 to (c) [$C_2$mim][$NTf_2$] data and (d) [$N_{4,1,1,1}$][$NTf_2$] data for varying $\Delta\Psi$ values. The black curves represent the raw data. Colour code: red, blue, green and magenta for 1 V, 2 V, 3 V and 4 V, respectively.

**Table 1.** Values of $\tau_1$, $c$, $\tau_2$ and $\tau_3$ for each IL at varying $\Delta\Psi$ during the charging process.

| **[$C_2$mim][$NTf_2$]** | $\boldsymbol{\tau_1}$/ns | $\boldsymbol{c}$ | $\boldsymbol{\tau_2}$/ns | $\boldsymbol{\tau_3}$/ns | **[$N_{4,1,1,1}$][$NTf_2$]** | $\boldsymbol{\tau_1}$/ns | $\boldsymbol{c}$ | $\boldsymbol{\tau_2}$/ns | $\boldsymbol{\tau_3}$/ns |
|---|---|---|---|---|---|---|---|---|---|
| **-0.5 V** | 0.44 | 0.13 | 0.01 | 0.68 | **-0.5 V** | 18.96 | 0.69 | 17.35 | 0.41 |
| **-1.0 V** | 0.53 | 0.50 | 0.19 | 1.24 | **-1.0 V** | 13.46 | 0.73 | 10.67 | 0.28 |
| **-1.5 V** | 0.55 | 0.55 | 0.17 | 1.60 | **-1.5 V** | 7.04 | 0.65 | 9.27 | 0.33 |
| **-2.0 V** | 0.40 | 0.77 | 0.27 | 2.66 | **-2.0 V** | 4.79 | 0.58 | 8.77 | 0.49 |
| **0.5 V** | 0.43 | 0.13 | 0.01 | 0.68 | **0.5 V** | 16.66 | 0.68 | 17.57 | 0.39 |
| **1.0 V** | 0.52 | 0.48 | 0.18 | 1.20 | **1.0 V** | 12.83 | 0.72 | 10.65 | 0.27 |
| **1.5 V** | 0.54 | 0.55 | 0.17 | 1.59 | **1.5 V** | 6.89 | 0.65 | 9.31 | 0.34 |
| **2.0 V** | 0.40 | 0.77 | 0.27 | 2.68 | **2.0 V** | 4.72 | 0.58 | 8.70 | 0.47 |

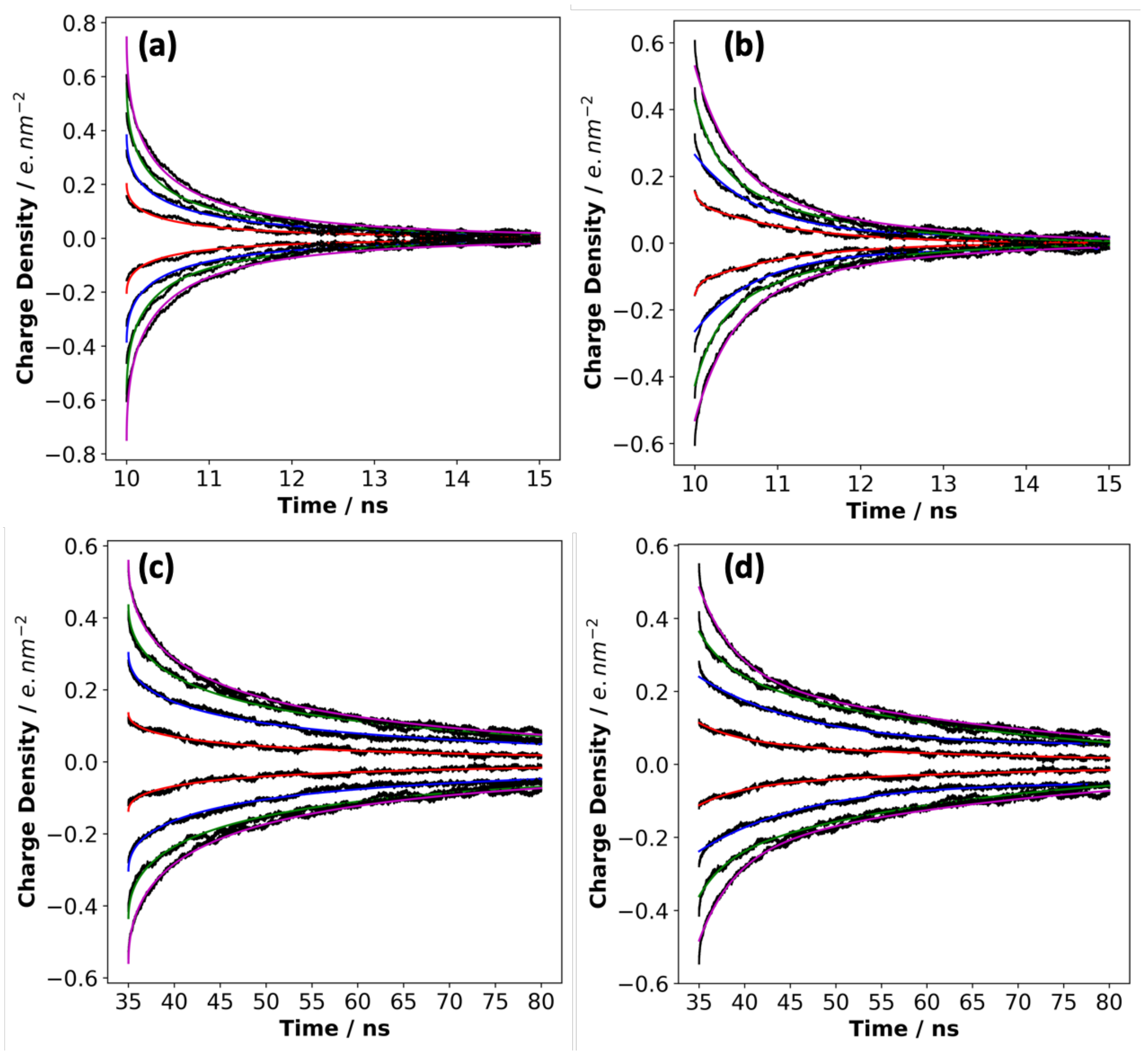


**Figure S5**. Charge density plots with fit curves for the discharging process. Fitting of Eqn. 1 to (a) [$C_2$mim][$NTf_2$] data and (b) [$N_{4,1,1,1}$][$NTf_2$] data. Fitting of Eqn. 2 to (c) [$C_2$mim][$NTf_2$] data and (d) [$N_{4,1,1,1}$][$NTf_2$] data for varying $\Delta\Psi$ values. The black curves represent the raw data. Colour code: red, blue, green and magenta for 1 V, 2 V, 3 V and 4 V, respectively.

**Table 2**. Values of $\tau_1$, $c$, $\tau_2$ and $\tau_3$for each IL at varying $\Delta\Psi$ during the discharging process.

| **[$C_2$mim][$NTf_2$]** | $\boldsymbol{\tau_4}$/ns | $\boldsymbol{c}$ | $\boldsymbol{\tau_5}$/ns | $\boldsymbol{\tau_6}$/ns | **[$N_{4,1,1,1}$][$NTf_2$]** | $\boldsymbol{\tau_4}$/ns | $\boldsymbol{c}$ | $\boldsymbol{\tau_5}$/ns | $\boldsymbol{\tau_6}$/ns |
|---|---|---|---|---|---|---|---|---|---|
| **-0.5 V** | 0.41 | 0.78 | 1.13 | 0.05 | **-0.5 V** | 10.15 | 0.54 | 32.66 | 4.58 |
| **-1.0 V** | 0.44 | 0.10 | 10.16 | 0.77 | **-1.0 V** | 13.18 | 0.21 | 3.1E+8 | 11.76 |
| **-1.5 V** | 0.37 | 0.44 | 0.25 | 1.35 | **-1.5 V** | 13.37 | 0.71 | 30.27 | 3.12 |
| **-2.0 V** | 0.37 | 0.62 | 0.42 | 1.78 | **-2.0 V** | 10.87 | 0.51 | 36.49 | 3.94 |
| **0.5 V** | 0.43 | 0.78 | 1.18 | 0.06 | **0.5 V** | 11.39 | 0.53 | 36.83 | 4.60 |
| **1.0 V** | 0.46 | 0.11 | 10.72 | 0.76 | **1.0 V** | 13.85 | 0.22 | 3.1E+8 | 11.77 |
| **1.5 V** | 0.38 | 0.44 | 0.25 | 1.39 | **1.5 V** | 13.83 | 0.71 | 31.02 | 3.13 |
| **2.0 V** | 0.38 | 0.64 | 0.44 | 1.88 | **2.0 V** | 11.20 | 0.51 | 37.46 | 3.95 |

## 4. Charge Distribution on Electrodes

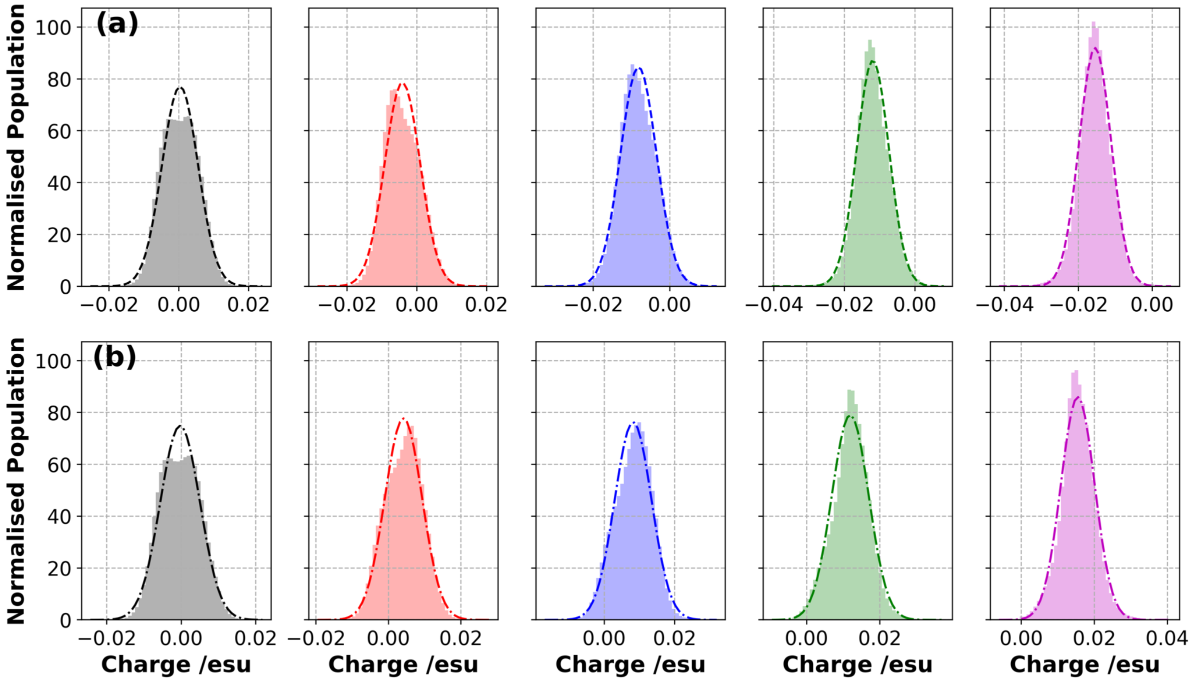


**Figure S6**. Histograms of the charges on the electrode atoms with varying $\Delta\Psi$ values for (a) the negative electrode and (b) positive electrode in the presence of the $[C_2mim][NTf_2]$ IL electrolyte. Colour code: black, red, blue, green and magenta for 0 V, 1 V, 2 V, 3 V and 4 V, respectively.

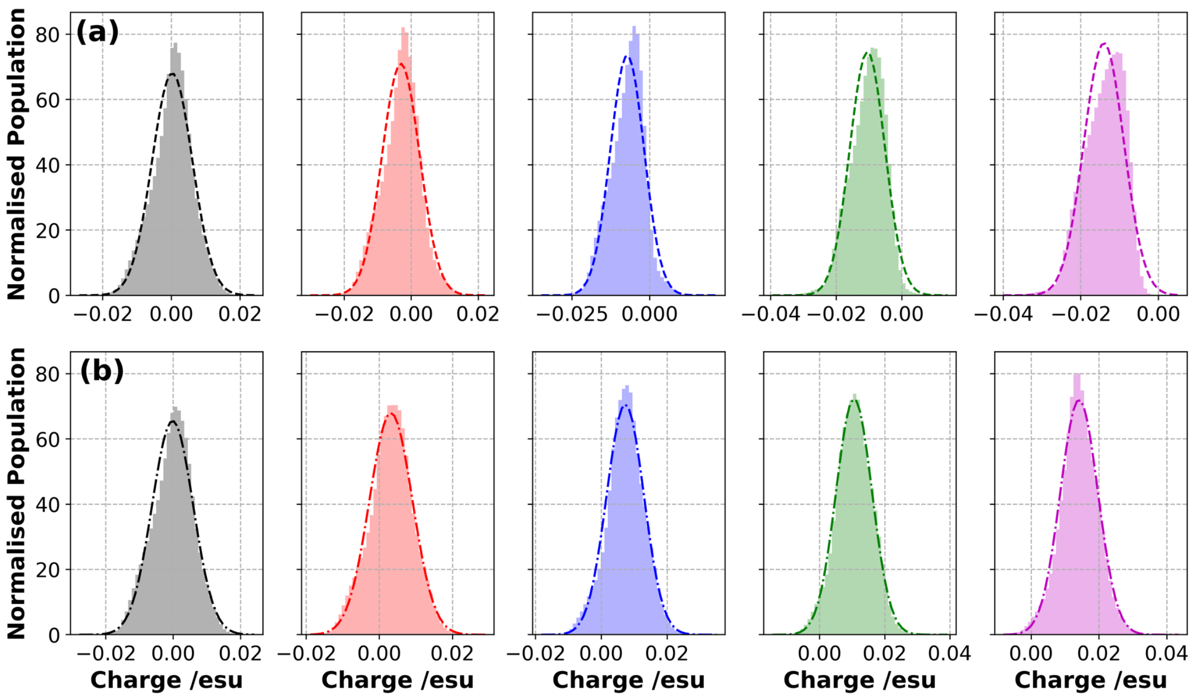


**Figure S7**. Histogram for the charges of electrode atoms for varying $\Delta\Psi$ values for (a) the negative electrode and (b) positive electrode in the presence of the $[N_{4,1,1,1}][NTf_2]$ IL electrolyte. Colour code: black, red, blue, green and magenta for 0 V, 1 V, 2 V, 3 V and 4 V, respectively.

**5. Charge evolution**

**Figure S8** shows the sum of the partial charges of the atoms found within a specific distance from the electrode surface for the $[C_2mim][NTf_2]$ system at $\Delta\Psi = 4$ V. Our results indicate that the total charge accumulated within a distance of 5 Å from the negative electrode surface has a positive value. This reflects the presence of $[C_2mim]^+$ ions. This could also be attributed to the presence of positively charged atoms (e.g. hydrogen atoms of the $[C_2mim]^+$ ions) found within this distance even if some atoms of the $[C_2mim]^+$ ions are found outside this 5 Å-thick layer.

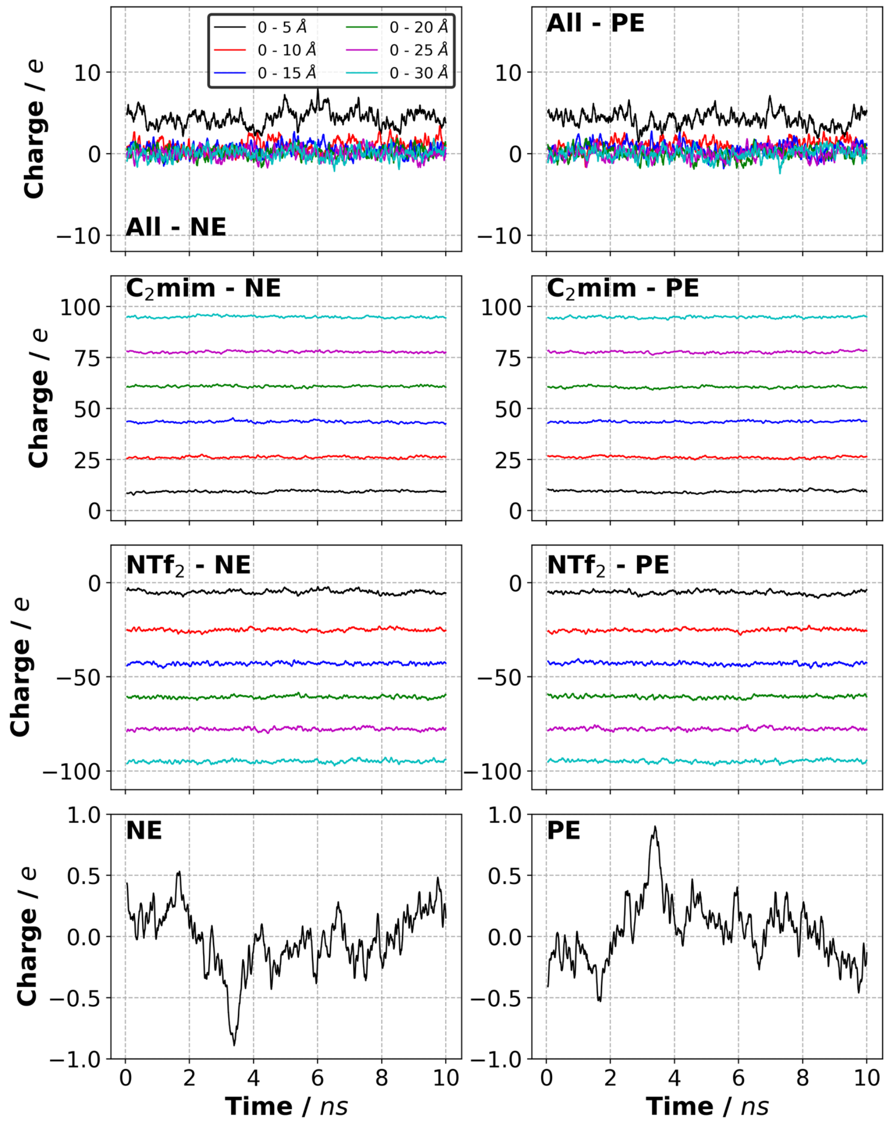


**Figure S8.** Sum of the partial charges of the atoms found within a specific distance from the electrode surface for the $[C_2mim][NTf_2]$ system at $\Delta\Psi = 0$ V and 294 K. Each data point represents a value averaged over 50 ps. NE and PE represent negative electrode and positive electrode, respectively.

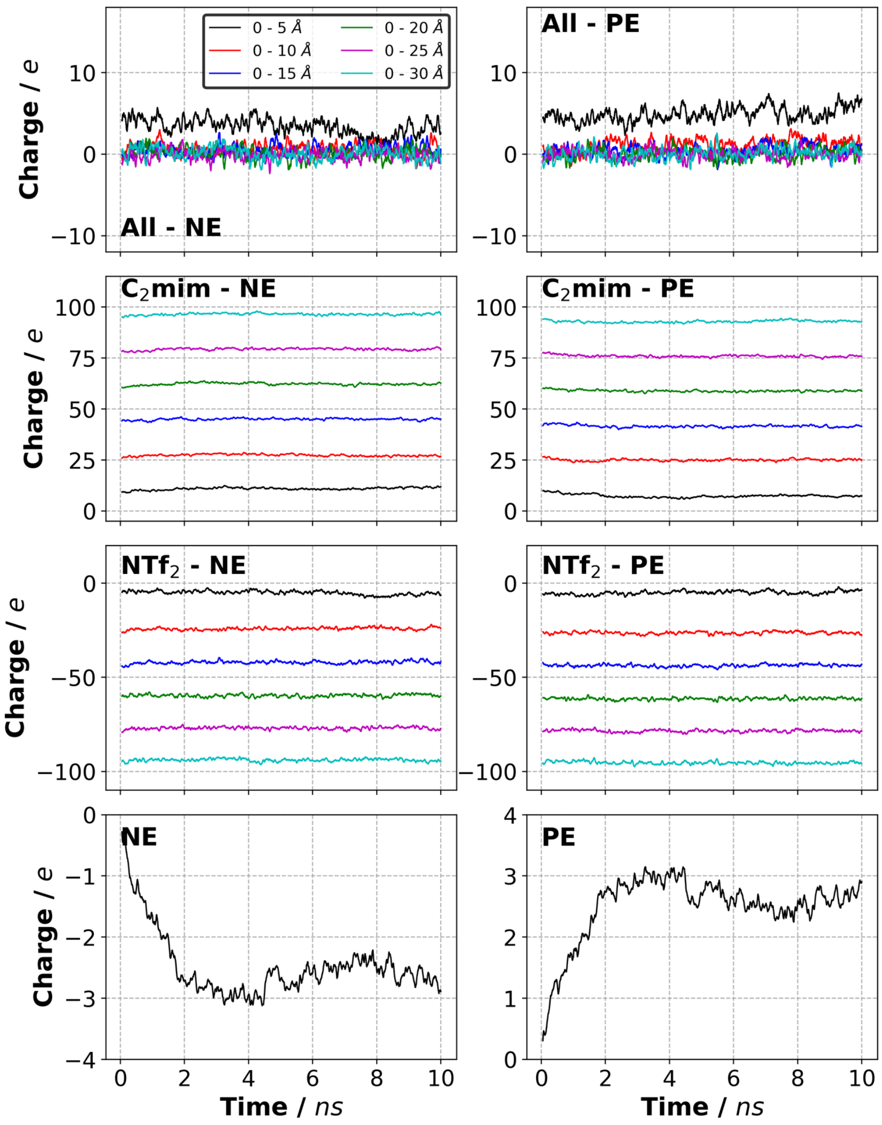


**Figure S9.** Sum of the partial charges of the atoms found within a specific distance from the electrode surface for the $[C_2mim][NTf_2]$ system at $\Delta\Psi$ = 1 V and 294 K. Each data point represents a value averaged over 50 ps. Ne and PE represent negative electrode and positive electrode, respectively.

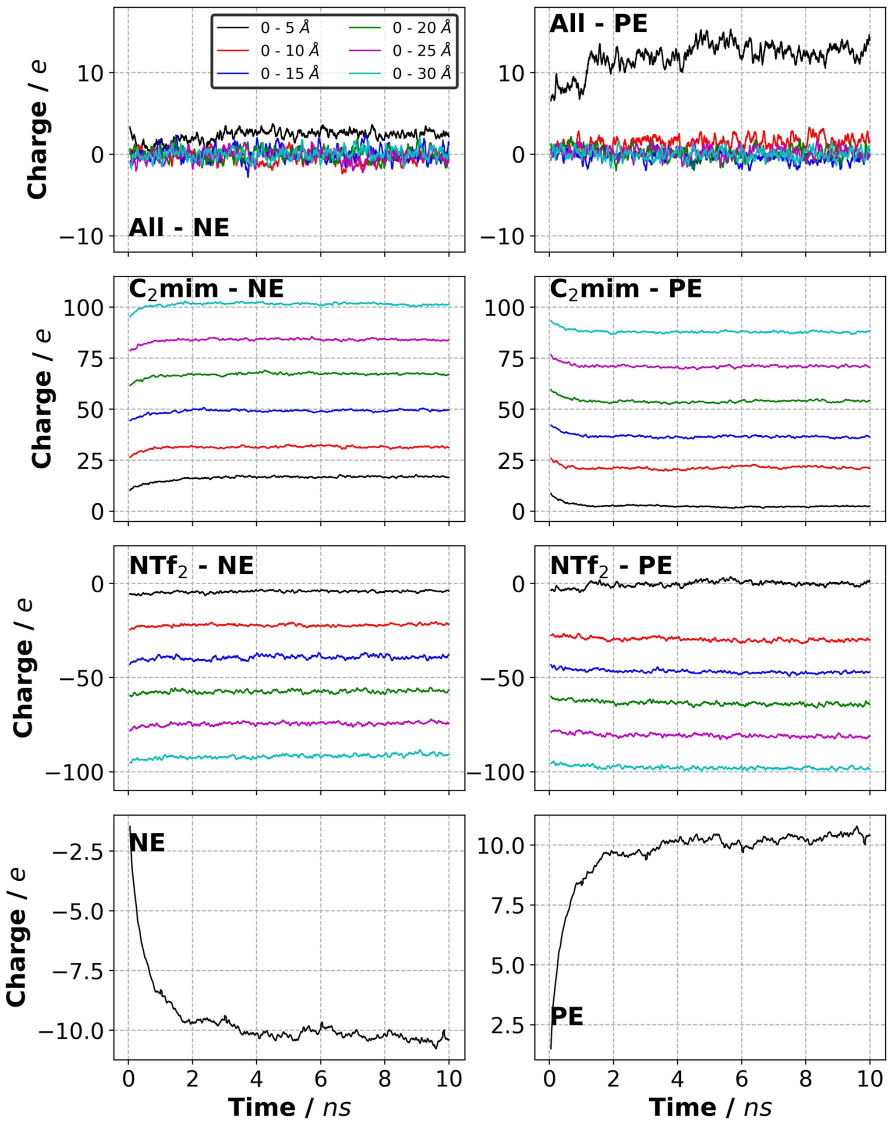


**Figure S10.** Sum of the partial charges of the atoms found within a specific distance from the electrode surface for the [$C_2$mim][$NTf_2$] system at $\Delta\Psi$ = 4 V and 294 K. Each data point represents a value averaged over 50 ps. NE and PE represent negative electrode and positive electrode, respectively.

When we calculated the total charge within the distance of 10 Å and beyond this distance from the negative electrode surface, it seems that the charge neutrality was satisfied.

Interestingly, the total charge calculated within the distance of 5 Å from the positive electrode surface, resulted in highly positive. This can be attributed to the fact that some of the [$C_2$mim] ions were still found in the vicinity of the positive electrode. In other words, the ratio of [$C_2$mim]/[$NTf_2$] on the negative electrode is larger than the ratio of [$NTf_2$]/[$C_2$mim] on the positive electrode for a distance of 5 Å.

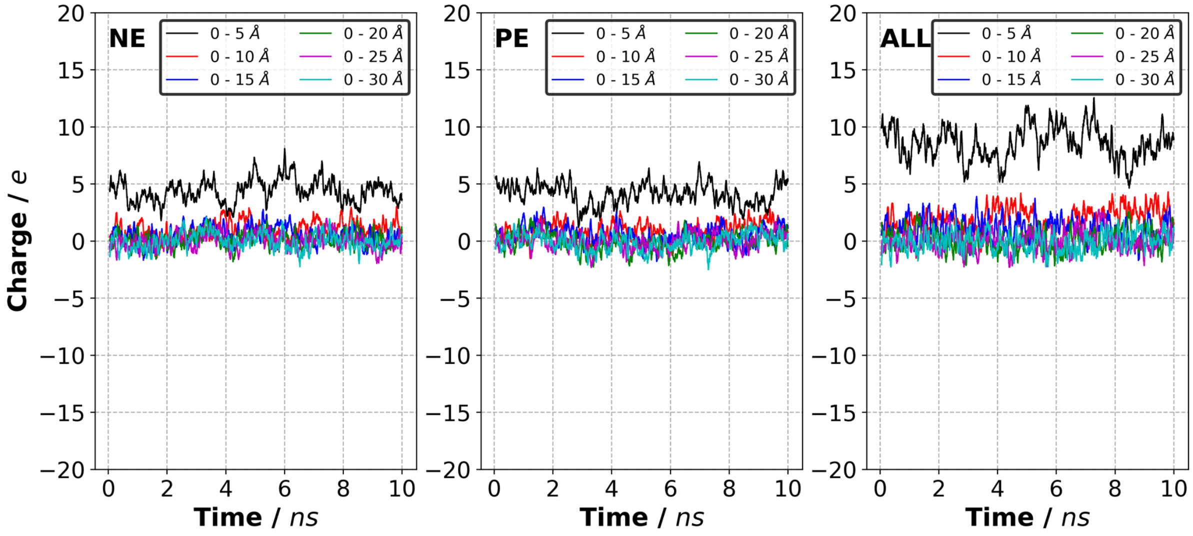


**Figure S11.** Sum of the partial charges of the $C_2$mim and $NTf_2$ atoms found within a specific distance from each electrode surface for the [$C_2$mim][$NTf_2$] system at $\Delta\Psi$ = 0 V and 294 K. Each data point represents a value averaged over 50 ps. NE and PE represent negative electrode and positive electrode, respectively. ALL stands for the entire supercapacitor system.

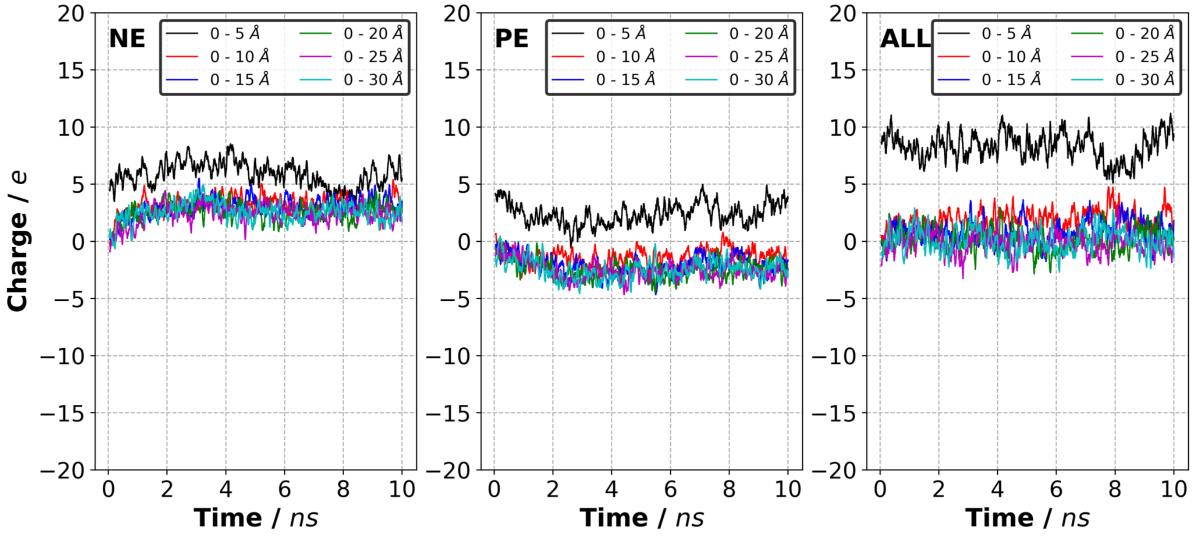


**Figure S12.** Sum of the partial charges of the $C_2$mim and $NTf_2$ atoms found within a specific distance from each electrode surface for the [$C_2$mim][$NTf_2$] system at $\Delta\Psi$ = 1 V and 294 K. Each data point represents a value averaged over 50 ps. NE and PE represent negative electrode and positive electrode, respectively. ALL stands for the entire supercapacitor system.

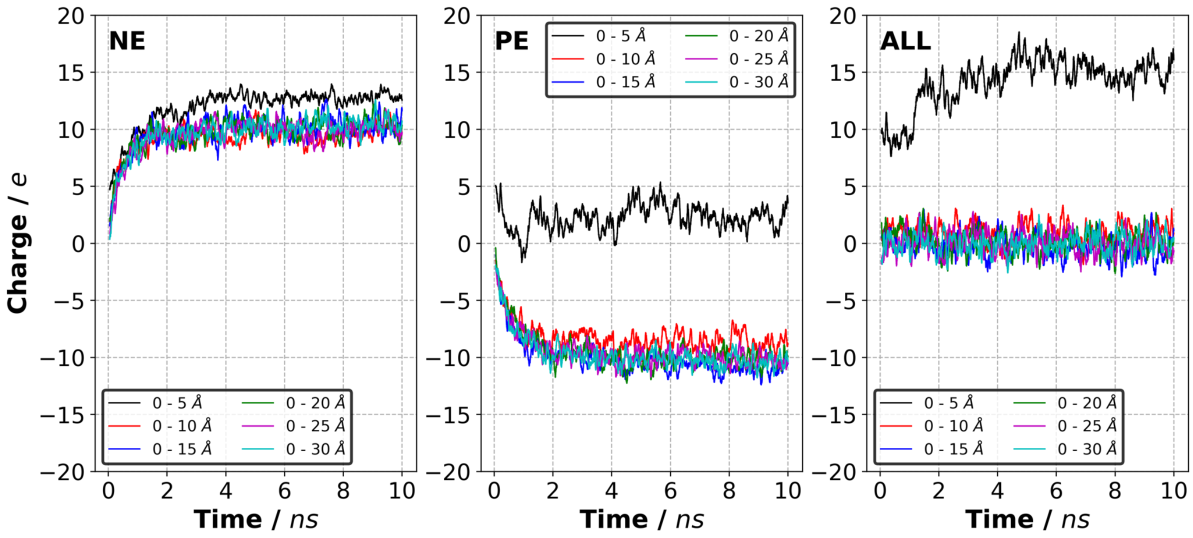


**Figure S13.** Sum of the partial charges of the $C_2$mim and $NTf_2$ atoms found within a specific distance from each electrode surface for the [$C_2$mim][$NTf_2$] system at $\Delta\Psi$ = 4 V and 294 K. Each data point represents a value averaged over 50 ps. NE and PE represent negative electrode and positive electrode, respectively. ALL stands for the entire supercapacitor system.

## 6. Life probability of ions in the electric double layer

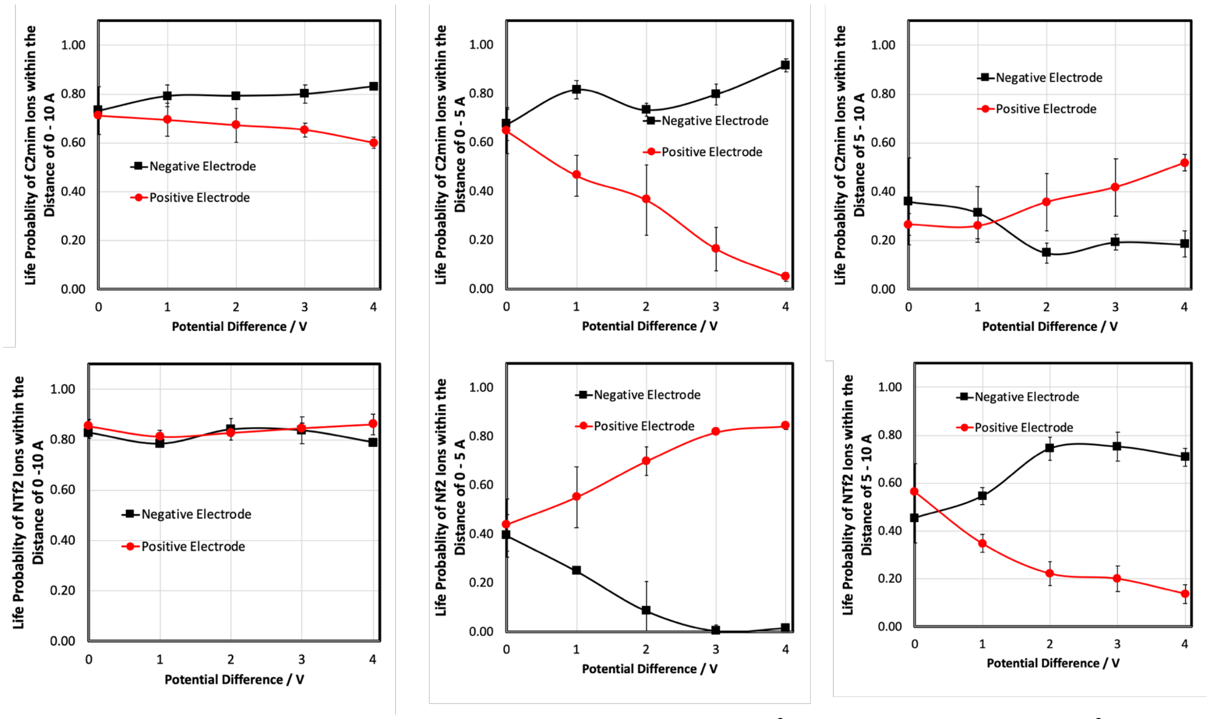


**Figure S14.** Life probability of ions in the entire layer (0-10 Å), in the first layer (0-5 Å) and in the second layer (5-10 Å) from the electrode surface as a function of potential difference at 294 K in the [$C_2$mim][$NTf_2$]. Error bars are the standard deviation of 3 sets of data.

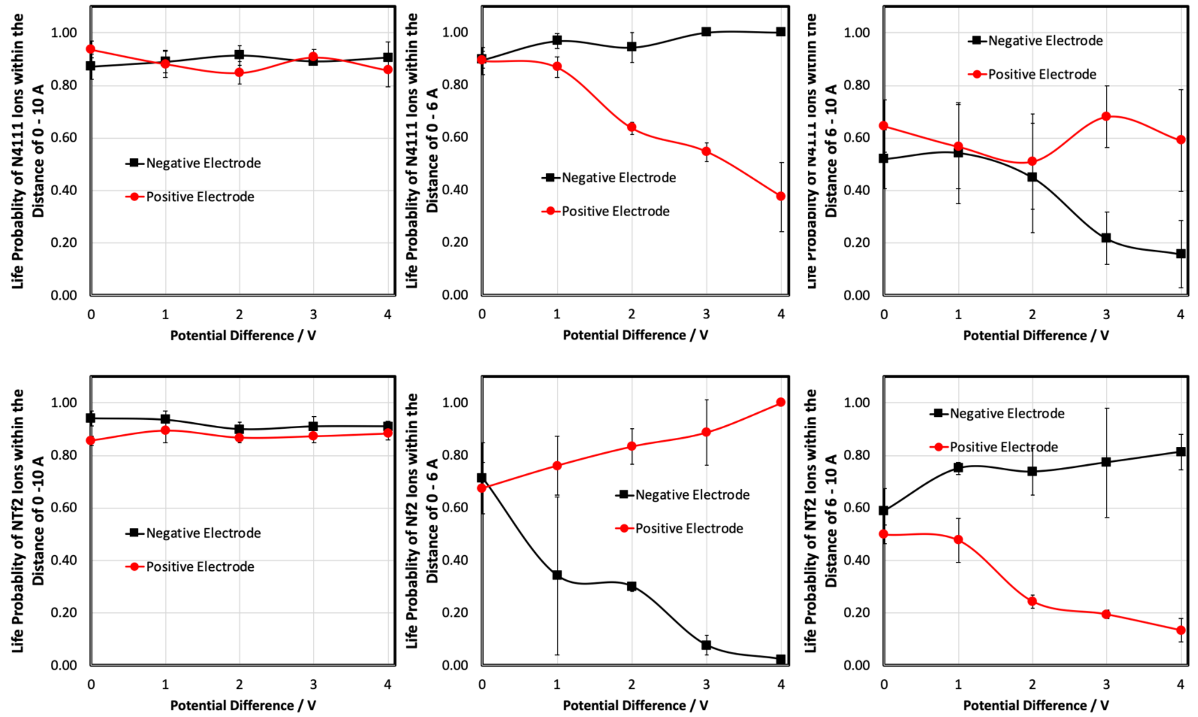


**Figure S15.** Life probability of ions in the entire layer (0-10 Å), in the first layer (0-6 Å) and in the second layer (6-10 Å) from the electrode surface as a function of potential difference at 294 K in [$N_{4,1,1,1}$][$NTf_2$]. Error bars are the standard deviation of 3 sets of data.

## 7. Angle Distribution Analyses

**Figure S16** shows the reference points used to calculate the angle distribution of ions as a function of distance from the electrode surface.

We calculated the angle between the electrode surface normal and the line that is the cross product, $\boldsymbol{v}_i \times \boldsymbol{w}_i$ ($i = n$, $c$ and $a$ for $[NTf_2]^-$, $[C_2mim]^+$ and $[N_{4,1,1,1}]^+$, respectively and the vectors are indicated on the diagrams of each ion). For the $[C_2mim]^+$ ion, a value of 0° or 180° means the aromatic ring of the $[C_2mim]^+$ ion is parallel to the electrode surface, while a perpendicular configuration is obtained when the angle is close to 90°.

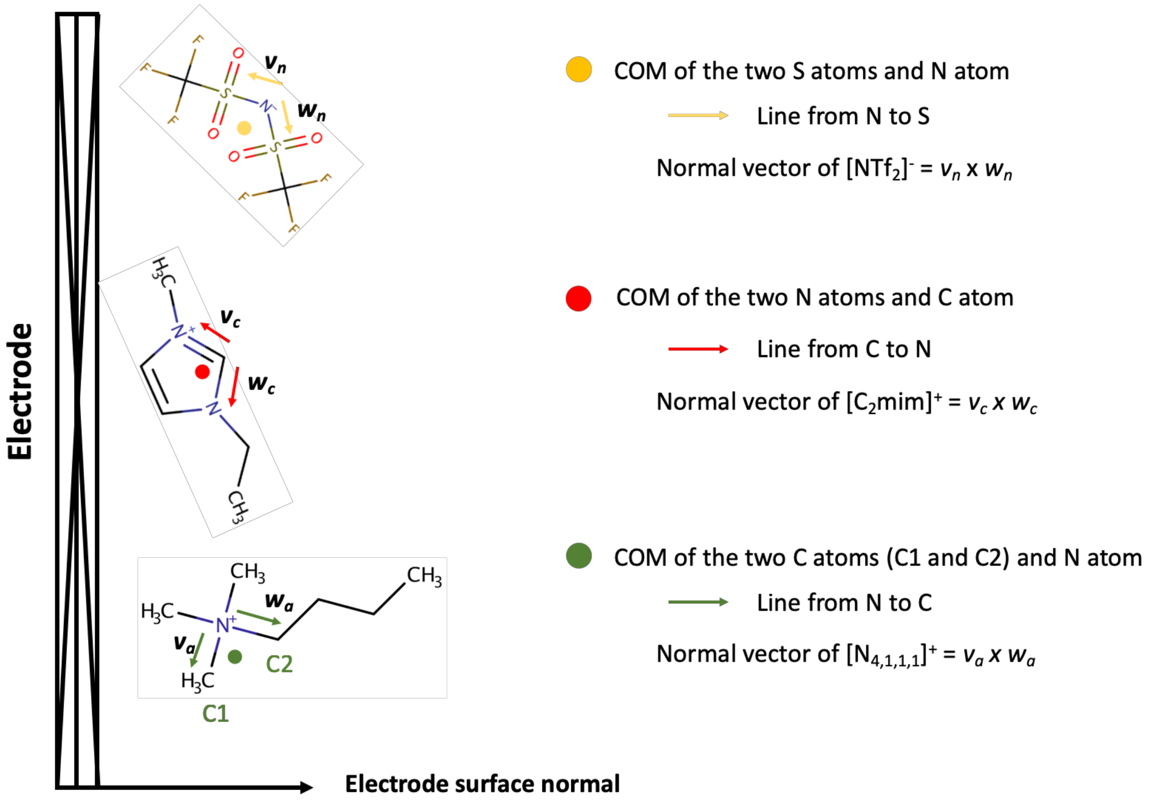


**Figure S16.** Reference points for calculation of the mass density distribution and vectors for the calculation of angle distribution for each ion type.

**Figures S17-S21** show the angle distribution for the $[C_2mim]^+$ ions in different layers from the electrode surfaces in the $[C_2mim][NTf_2]$ IL for each potential difference investigated in this study.

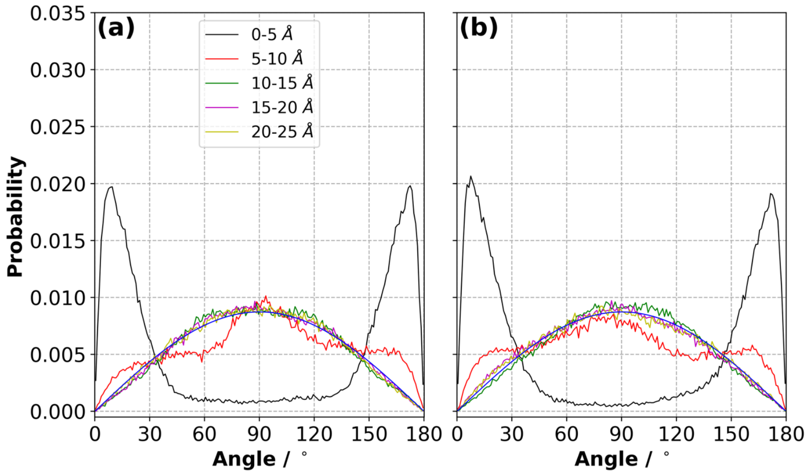


**Figure S17.** Angle distribution for ions in layers (up to 20 Å from each electrode) for the $[C_2mim]^+$ ions in the $[C_2mim][NTf_2^-$ IL on the (a) negative and (b) positive electrode at $\Delta\Psi = 0$ V and 294 K (averaged over three independently generated samples). The blue curve represents the ideal distribution in the bulk.

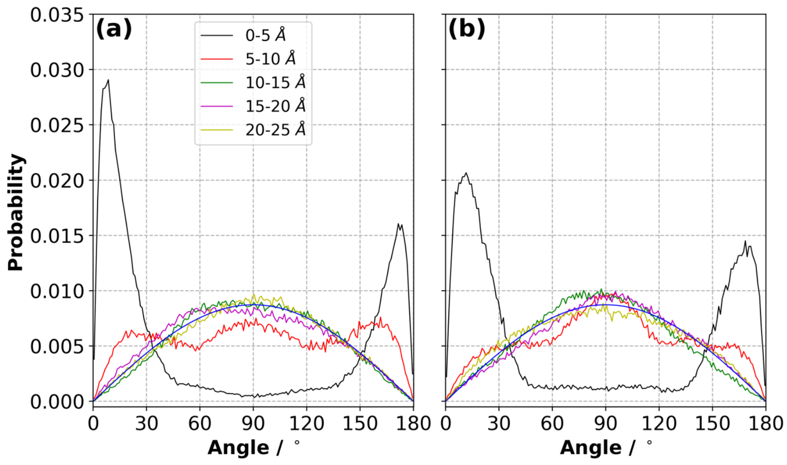


**Figure S18.** Angle distribution for ions in the layers (up to 20 Å from each electrode) for the $[C_2mim]^+$ ions in the $[C_2mim][NTf_2]$ IL on the (a) negative and (b) positive electrode at $\Delta\Psi = 1$ V and 294 K (averaged over three independently generated samples). The blue curve represents the ideal distribution in the bulk.

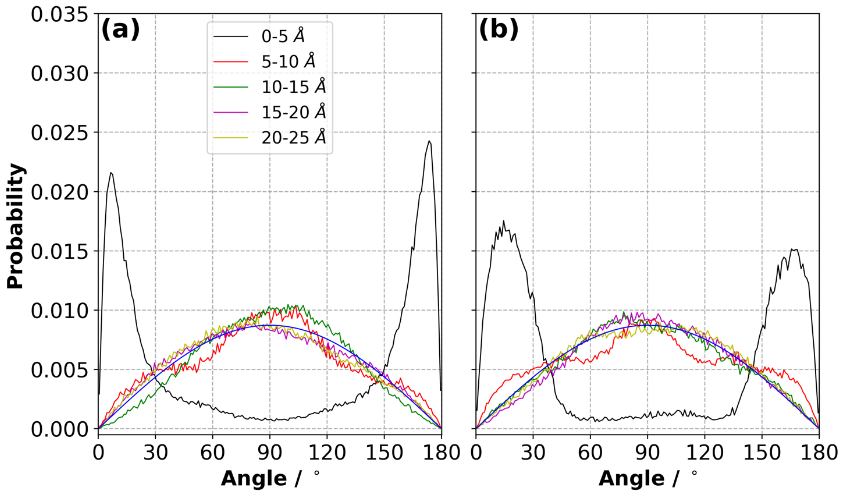


**Figure S19.** Angle distribution for ions in the layers (up to 20 Å from each electrode) for the $[C_2mim]^+$ ions in the $[C_2mim][NTf_2]$ IL on the (a) negative and (b) positive electrode at $\Delta\Psi$ = 2 V and 294 K (averaged over three independently generated samples). The blue curve represents the ideal distribution in the bulk.

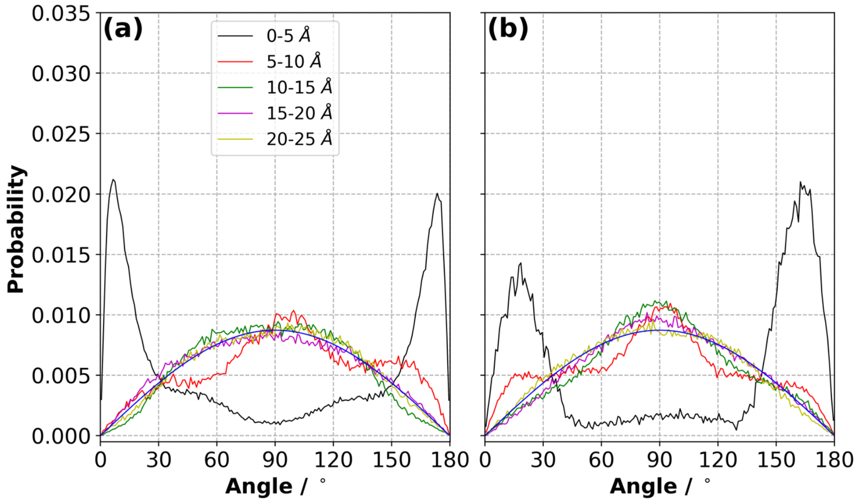


**Figure S20.** Angle distribution for ions in the layers (up to 20 Å from each electrode) for the $[C_2mim]^+$ ions in the $[C_2mim][NTf_2]$ IL on the (a) negative and (b) positive electrode at $\Delta\Psi$ = 3 V and 294 K (averaged over three independently generated samples). The blue curve represents the ideal distribution in the bulk.

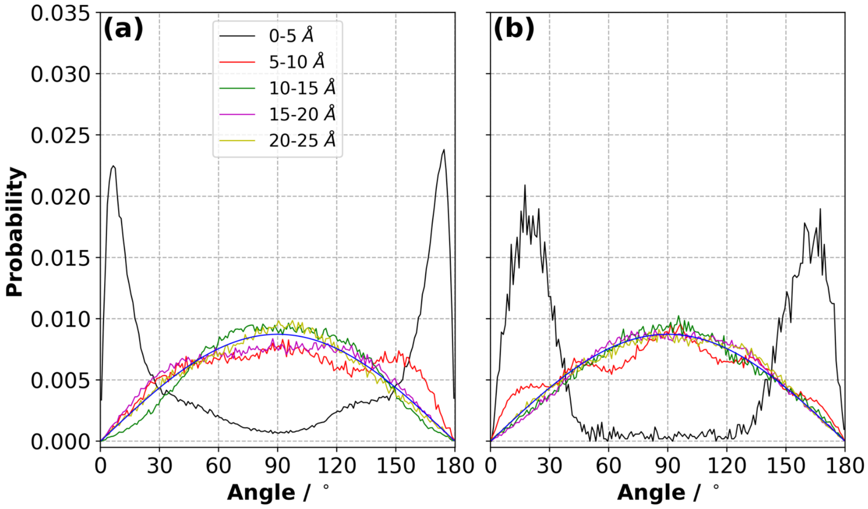


**Figure S21.** Angle distribution for ions in the layers (up to 20 Å from each electrode) for the $[C_2mim]^+$ ions in the $[C_2mim][NTf_2]$ IL on the (a) negative and (b) positive electrode at $\Delta\Psi$ = 4 V and 294 K (averaged over three independently generated samples). The blue curve represents the ideal distribution in the bulk.

**Figures S22-S26** show the angle distribution for the $[NTf_2]^-$ ions in different layers from the electrode surfaces in the $[C_2mim][NTf_2]$ IL for each potential difference investigated in this study.

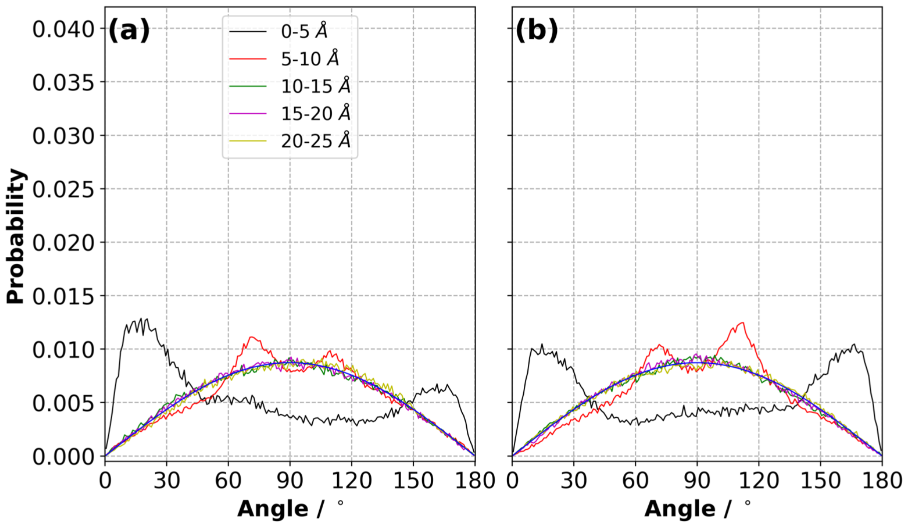


**Figure S22.** Angle distribution for ions in the layers (up to 20 Å from each electrode) for the $[NTf_2]^-$ ions in the $[C_2mim][NTf_2]$ IL on the (a) negative and (b) positive electrode at $\Delta\Psi$ = 0 V and 294 K (averaged over three independently generated samples). The blue curve represents the ideal distribution in the bulk.

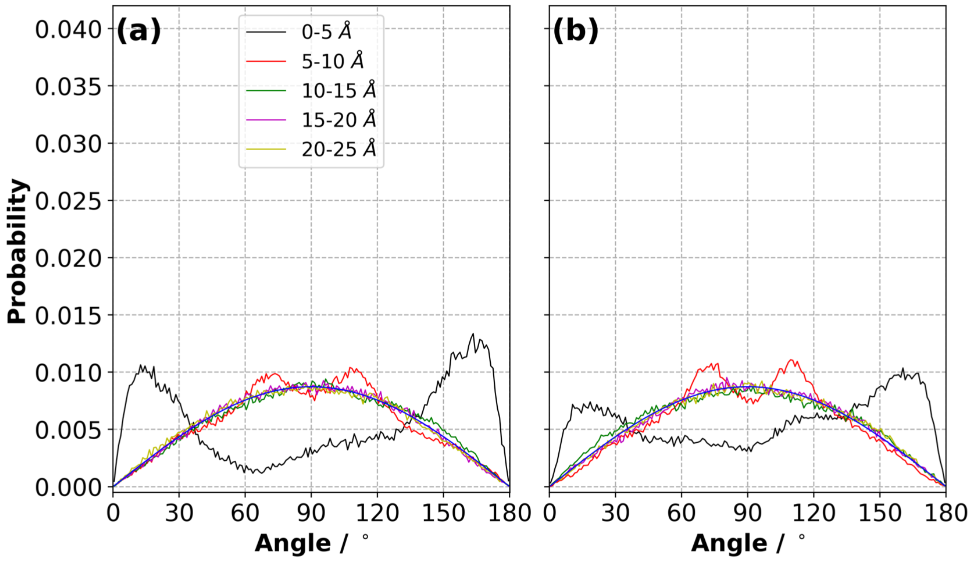


**Figure S23.** Angle distribution for ions in the layers (up to 20 Å from each electrode) for the $[NTf_2]^-$ ions in the $[C_2mim][NTf_2]$ IL the on (a) negative and (b) positive electrode at $\Delta\Psi$ = 1 V and 294 K (averaged over three independently generated samples). The blue curve represents the ideal distribution in the bulk.

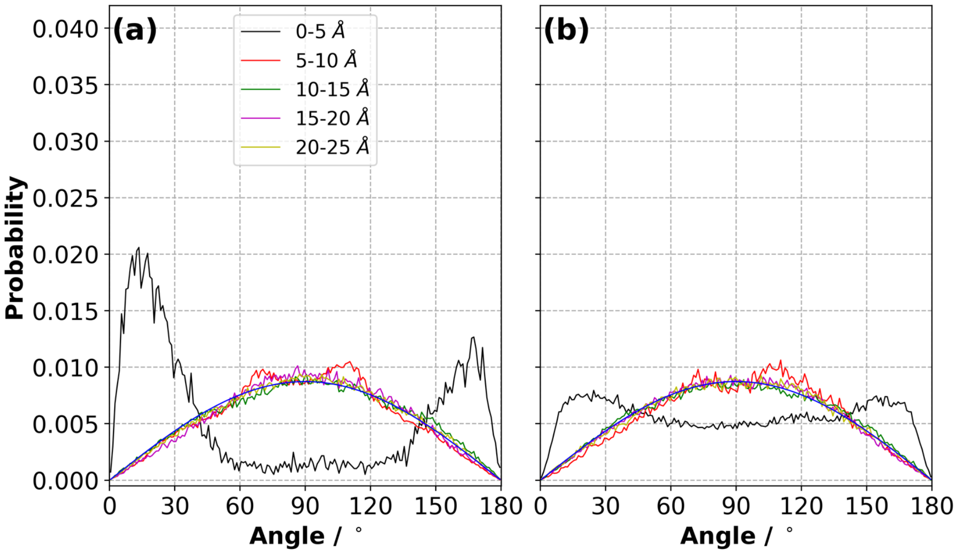


**Figure S24.** Angle distribution for ions in the layers (up to 20 Å from each electrode) for the $[NTf_2]^-$ ions in the $[C_2mim][NTf_2]$ IL on the (a) negative and (b) positive electrode at $\Delta\Psi$ = 2 V and 294 K (averaged over three independently generated samples). The blue curve represents the ideal distribution in the bulk.

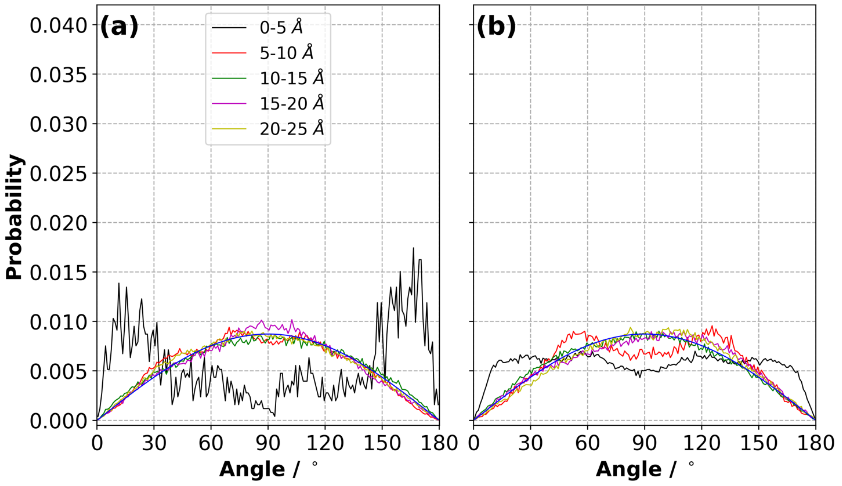


**Figure S25.** Angle distribution for ions in the layers (up to 20 Å from each electrode) for the $[NTf_2]^-$ ions in the $[C_2mim][NTf_2]$ IL on the (a) negative and (b) positive electrode at $\Delta\Psi$ = 3 V and 294 K (averaged over three independently generated samples). The blue curve represents the ideal distribution in the bulk.

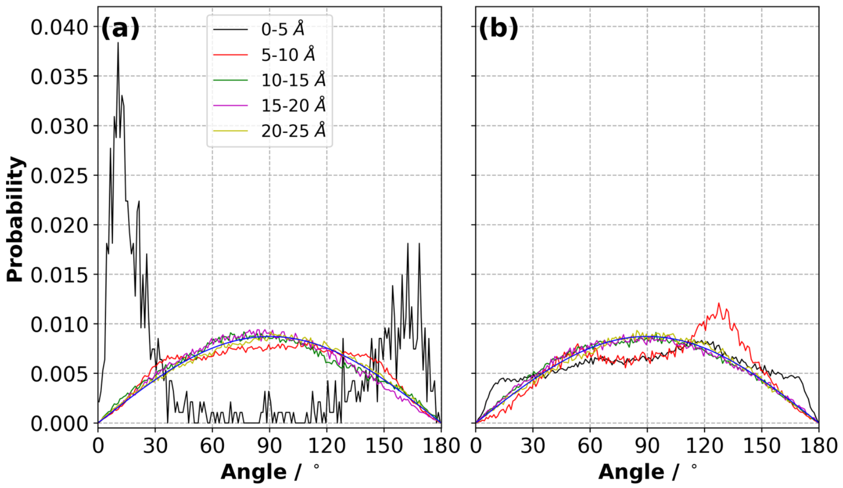


**Figure S26.** Angle distribution for ions in the layers (up to 20 Å from each electrode) for the $[NTf_2]^-$ ions in the $[C_2mim][NTf_2]$ IL on the (a) negative and (b) positive electrode at $\Delta\Psi$ = 4 V and 294 K (averaged over three independently generated samples). The blue curve represents the ideal distribution in the bulk.

**Figures S27-S28** show the angle distribution for the $[C_2mim]^+$ and $[NTf_2]^-$ ions in the first layer in the $[C_2mim][NTf_2]$ IL for each potential difference investigated in this study, respectively.

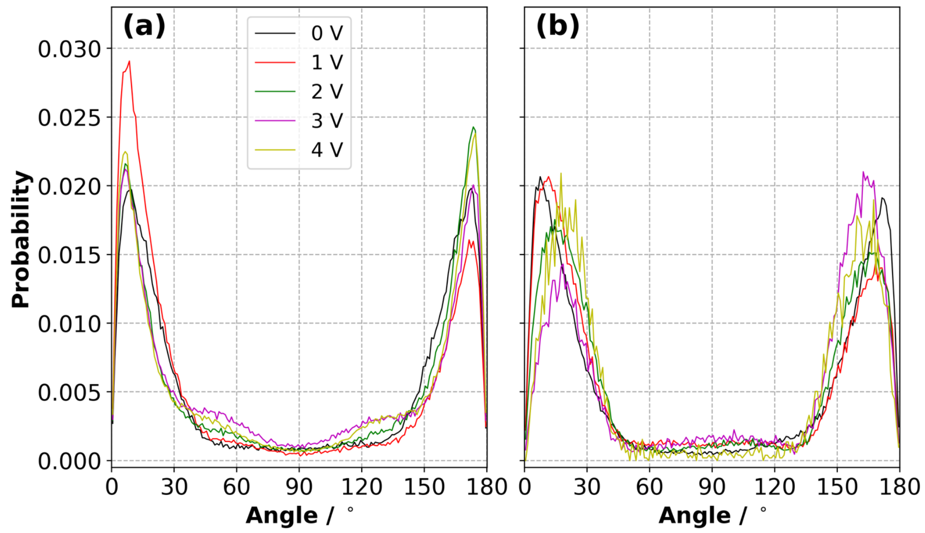


**Figure S27.** Angle distribution in the first layer (up to 5 Å from each electrode) for the $[C_2mim]^+$ ions in the $[C_2mim][NTf_2]$ IL on the (a) negative and (b) positive electrode for all $\Delta\Psi$ at 294 K (averaged over three independently generated samples).

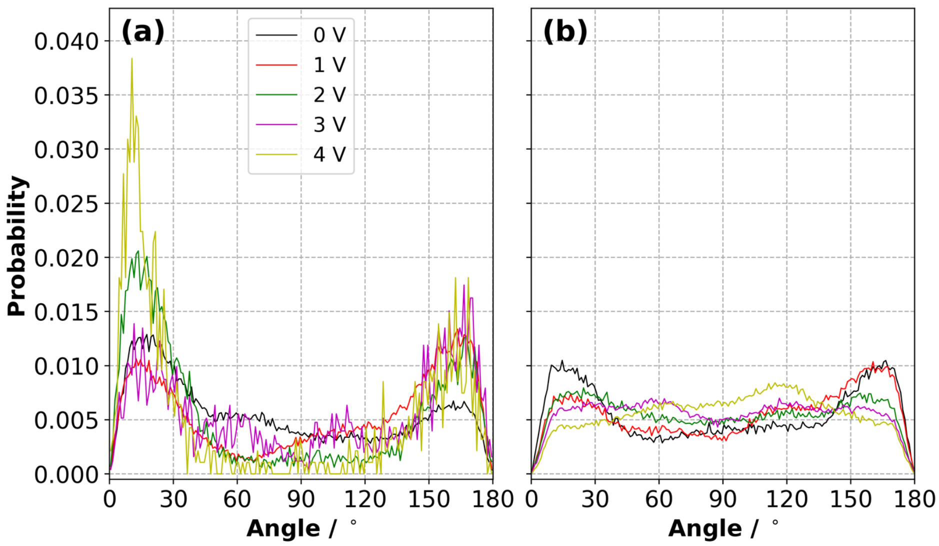


**Figure S28.** Angle distribution in the first layers (up to 5 Å from each electrode) for the $[NTf_2]^-$ ions in the $[C_2mim][NTf_2]$ IL on the (a) negative and (b) positive electrode for all $\Delta\Psi$ at 294 K (averaged over three independently generated samples).

**Figures S29-S33** show the angle distribution for the $[N_{4,1,1,1}]^+$ ions in different layers from the electrode surfaces in the $[C_2mim][NTf_2]$ IL for each potential difference investigated in this study.

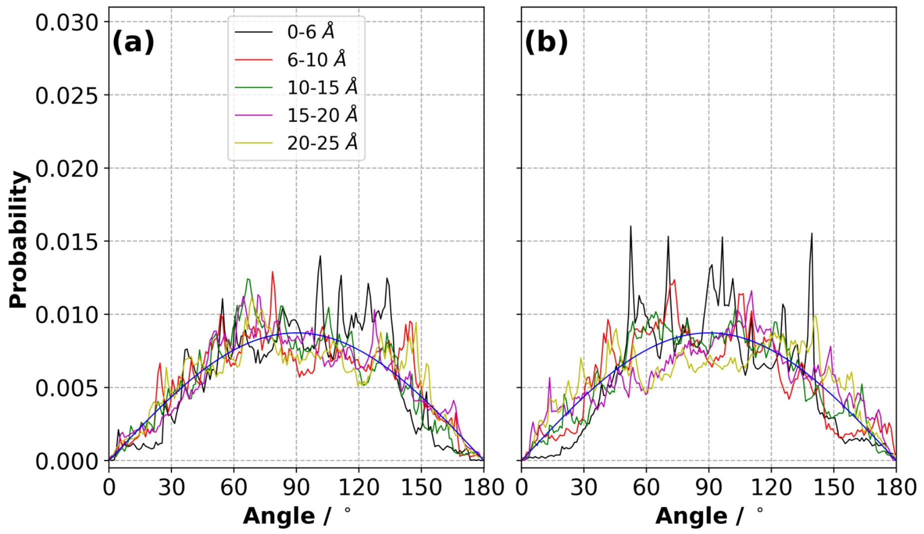


**Figure S29.** Angle distribution for ions in the layers (up to 20 Å from each electrode) for the $[N_{4,1,1,1}]^+$ ions in the $[N_{4,1,1,1}][NTf_2]$ IL on the (a) negative and (b) positive electrode at $\Delta\Psi$ = 0 V and 294 K (averaged over three independently generated samples).

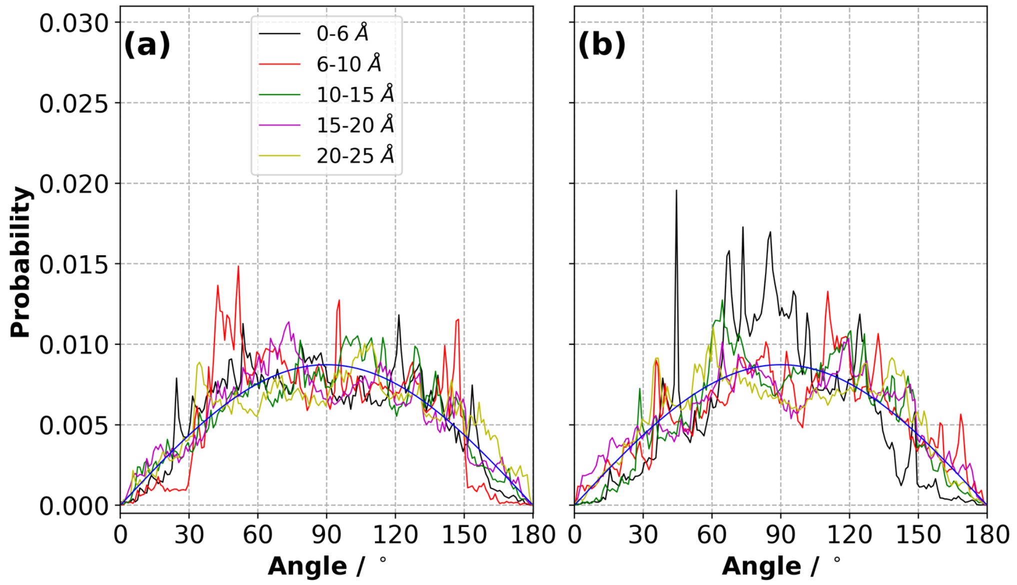


**Figure S30.** Angle distribution for ions in the layers (up to 20 Å from each electrode) for the $[N_{4,1,1,1}]^+$ ions in the $[N_{4,1,1,1}][NTf_2]$ IL on the (a) negative and (b) positive electrode at $\Delta\Psi$ = 1 V and 294 K (averaged over three independently generated samples).

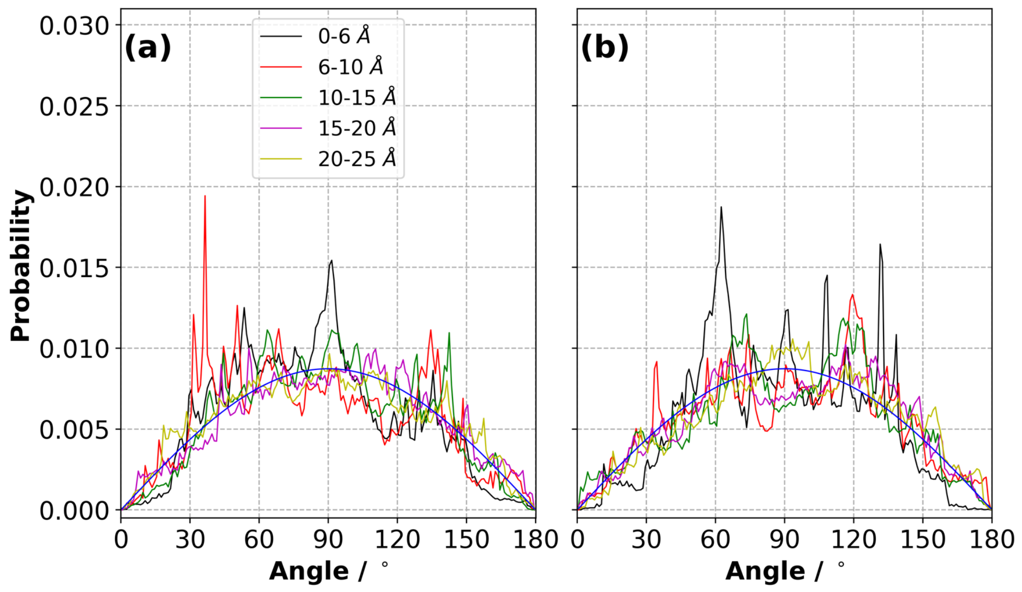


**Figure S31.** Angle distribution for ions in the layers (up to 20 Å from each electrode) for the $[N_{4,1,1,1}]^+$ ions in the $[N_{4,1,1,1}][NTf_2]$ IL on the (a) negative and (b) positive electrode at $\Delta\Psi$ = 2 V and 294 K (averaged over three independently generated samples).

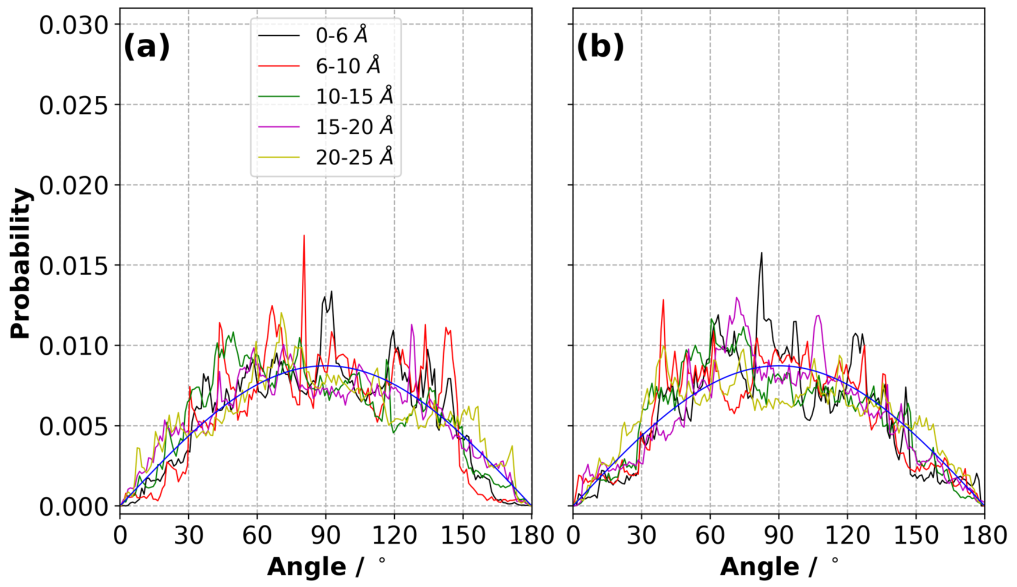


**Figure S32.** Angle distribution for ions in the layers (up to 20 Å from each electrode) for the $[N_{4,1,1,1}]^+$ ions in the $[N_{4,1,1,1}][NTf_2]$ IL on the (a) negative and (b) positive electrode at $\Delta\Psi$ = 3 V and 294 K (averaged over three independently generated samples).

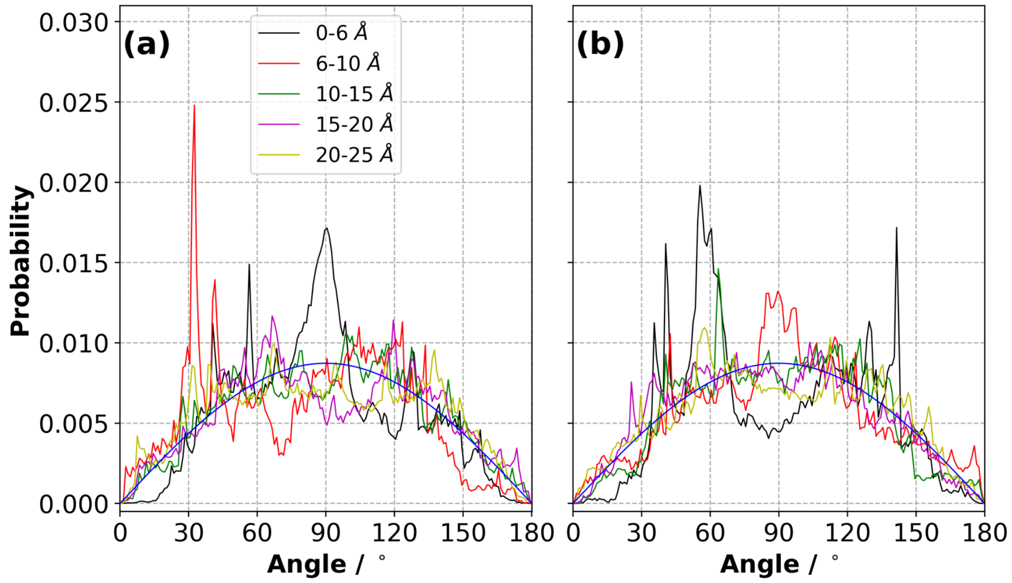


**Figure S33.** Angle distribution for ions in the layers (up to 20 Å from each electrode) for the $[N_{4,1,1,1}]^+$ ions in the $[N_{4,1,1,1}][NTf_2]$ IL on the (a) negative and (b) positive electrode at $\Delta\Psi$ = 4 V and 294 K (averaged over three independently generated samples).

**Figures S34-S38** show the angle distribution for the $[NTf_2]^-$ ions in different layers from the electrode surfaces in the $[N_{4,1,1,1}][NTf_2]$ IL for each potential difference investigated in this study.

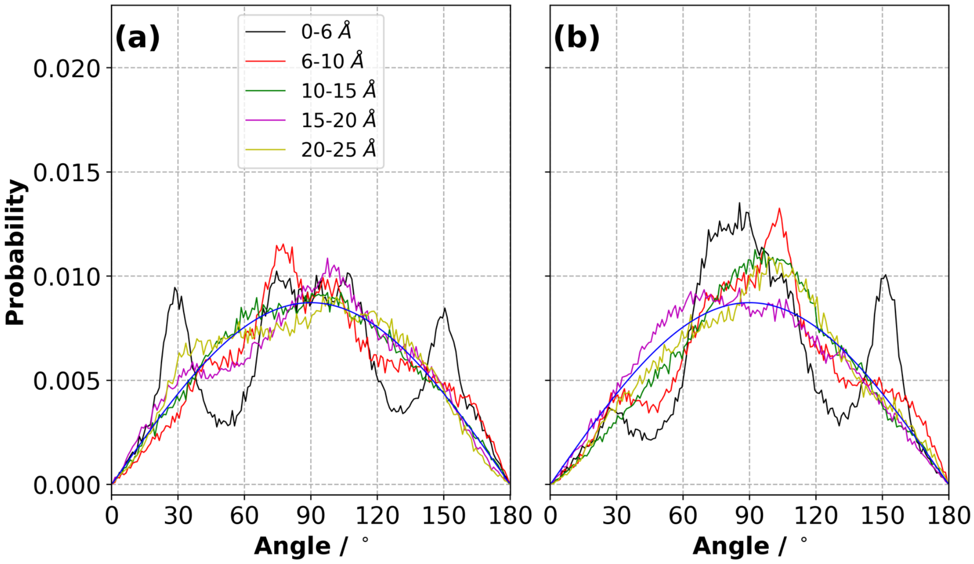


**Figure S34.** Angle distribution for ions in the layers (up to 20 Å from each electrode) for the $[NTf_2]^-$ ions in the $[N_{4,1,1,1}][NTf_2]$ IL on the (a) negative and (b) positive electrode at $\Delta\Psi$ = 0 V and 294 K (averaged over three independently generated samples).

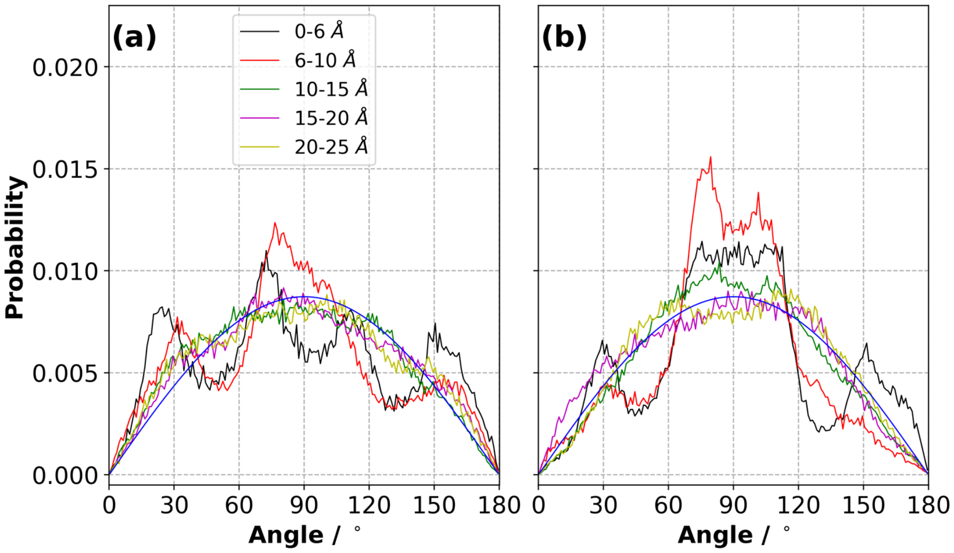


**Figure S35.** Angle distribution for ions in the layers (up to 20 Å from each electrode) for the $[NTf_2]^-$ ions in the $[N_{4,1,1,1}][NTf_2]$ IL on the (a) negative and (b) positive electrode at $\Delta\Psi$ = 1 V and 294 K (averaged over three independently generated samples).

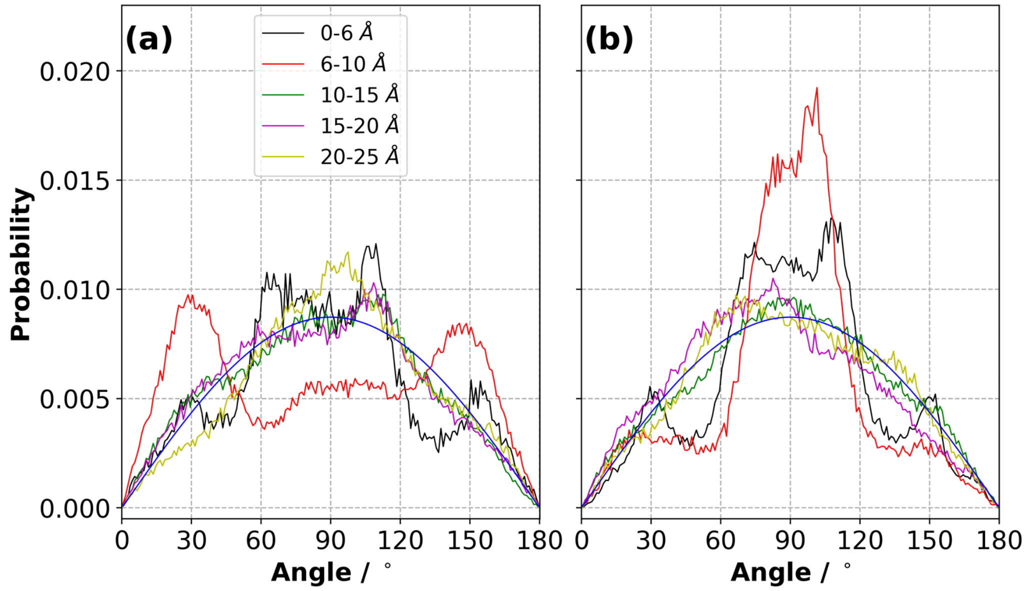


**Figure S36.** Angle distribution for ions in the layers (up to 20 Å from each electrode) for the $[NTf_2]^-$ ions in the $[N_{4,1,1,1}][NTf_2]$ IL on the (a) negative and (b) positive electrode at $\Delta\Psi$ = 2 V and 294 K (averaged over three independently generated samples).

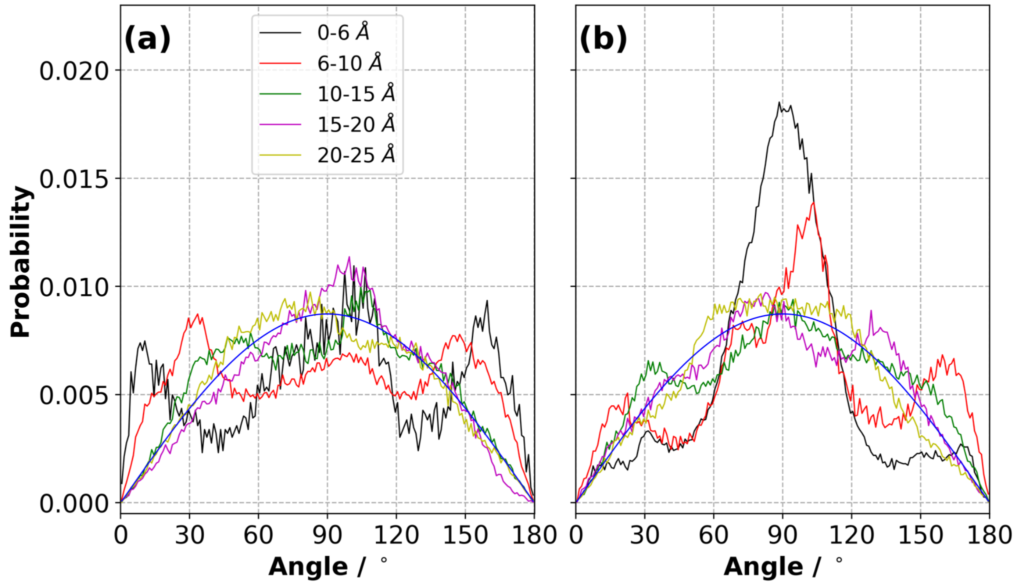


**Figure S37.** Angle distribution for ions in the layers (up to 20 Å from each electrode) for the $[NTf_2]^-$ ions in the $[N_{4,1,1,1}][NTf_2]$ IL on the (a) negative and (b) positive electrode at $\Delta\Psi$ = 3 V and 294 K (averaged over three independently generated samples).

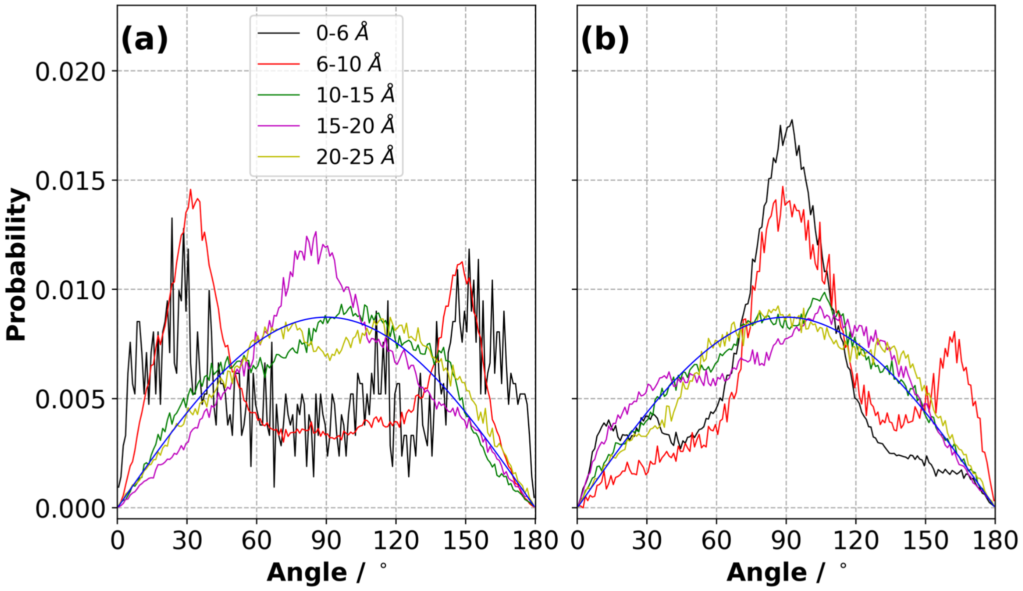


**Figure S38.** Angle distribution for ions in the layers (up to 20 Å from each electrode) for the $[NTf_2]^-$ ions in the $[N_{4,1,1,1}][NTf_2]$ IL on the (a) negative and (b) positive electrode at $\Delta\Psi$ = 4 V and 294 K (averaged over three independently generated samples).

**Figures S39-S40** show the angle distribution for the $[N_{4,1,1,1}]^+$ and $[NTf_2]^-$ ions in the first layer in the $[N_{4,1,1,1}][NTf_2]$ IL for each potential difference investigated in this study, respectively.

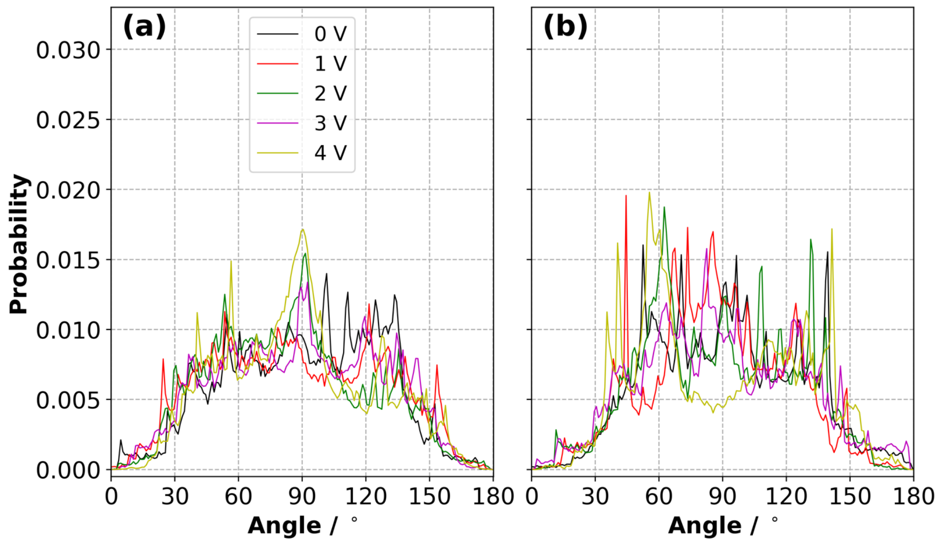


**Figure S39.** Angle distribution in the first layer (up to 6 Å from each electrode) for the $[N_{4,1,1,1}]^+$ ions in the $[N_{4,1,1,1}][NTf_2]$ IL on the (a) negative and (b) positive electrode for all $\Delta\Psi$ at 294 K (averaged over three independently generated samples).

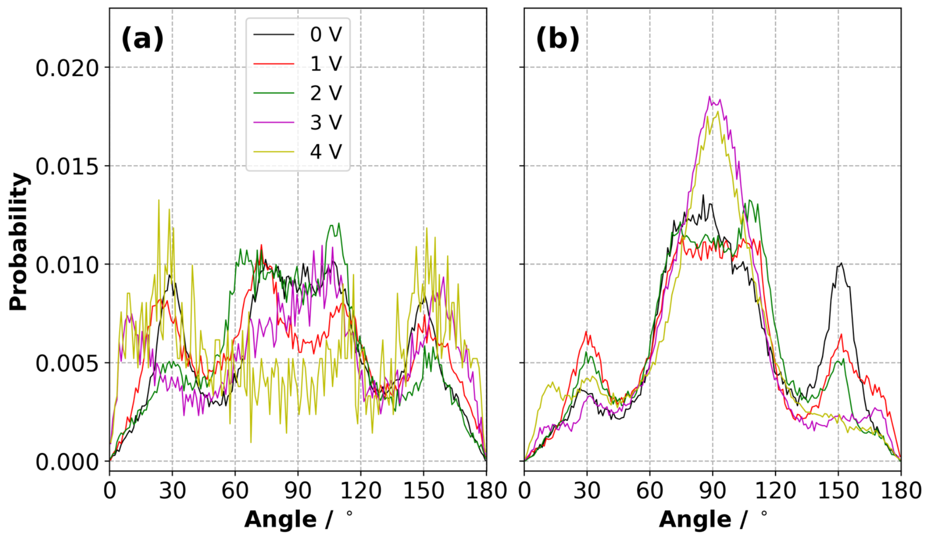


**Figure S40.** Angle distribution in the first layer (up to 6 Å from each electrode) for the $[NTf_2]^-$ ions in the $[N_{4,1,1,1}][NTf_2]$ IL on the (a) negative and (b) positive electrode for all $\Delta\Psi$ at 294 K (averaged over three independently generated samples).

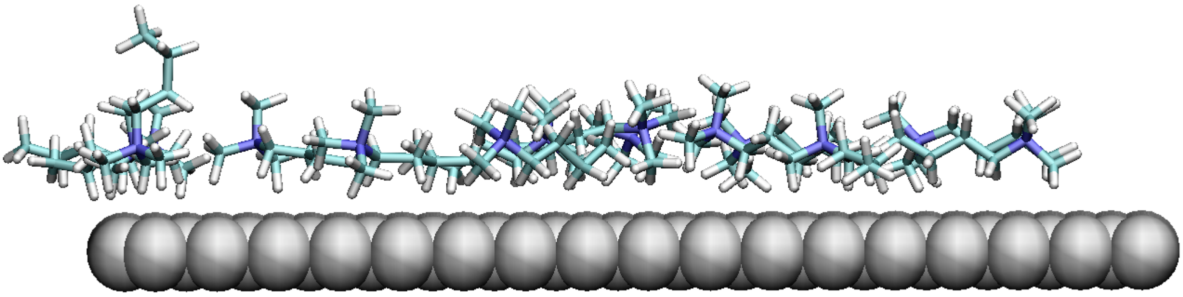

**Figure S41.** $[N_{4,1,1,1}]^+$ ions with one atom within 4 Å of the electrode $\Delta\Psi$ = 0 V. Ions adopt a parallel-like configuration on the electrode surface.

## 8. Mean square displacement curves

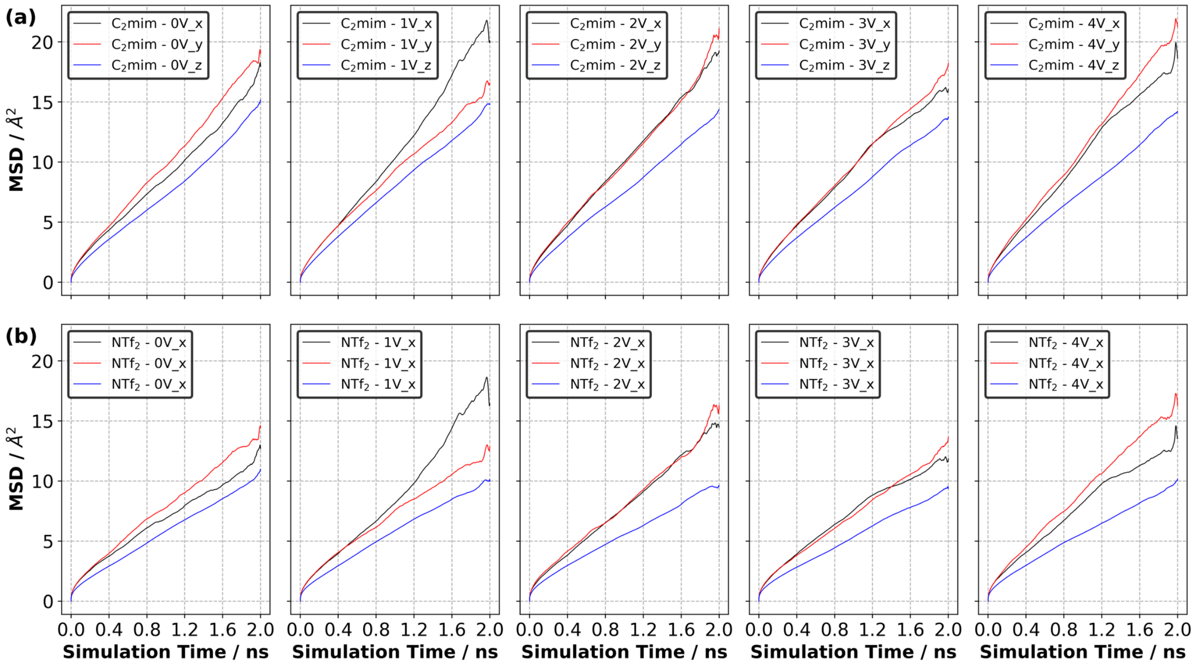


**Figure S42.** Calculated mean square displacement (MSD) at 294 K in each principal direction for (a) $[C_2mim]^+$ and (b) $[NTf_2]^-$ at each $\Delta\Psi$ value (averaged over three independently generated samples).

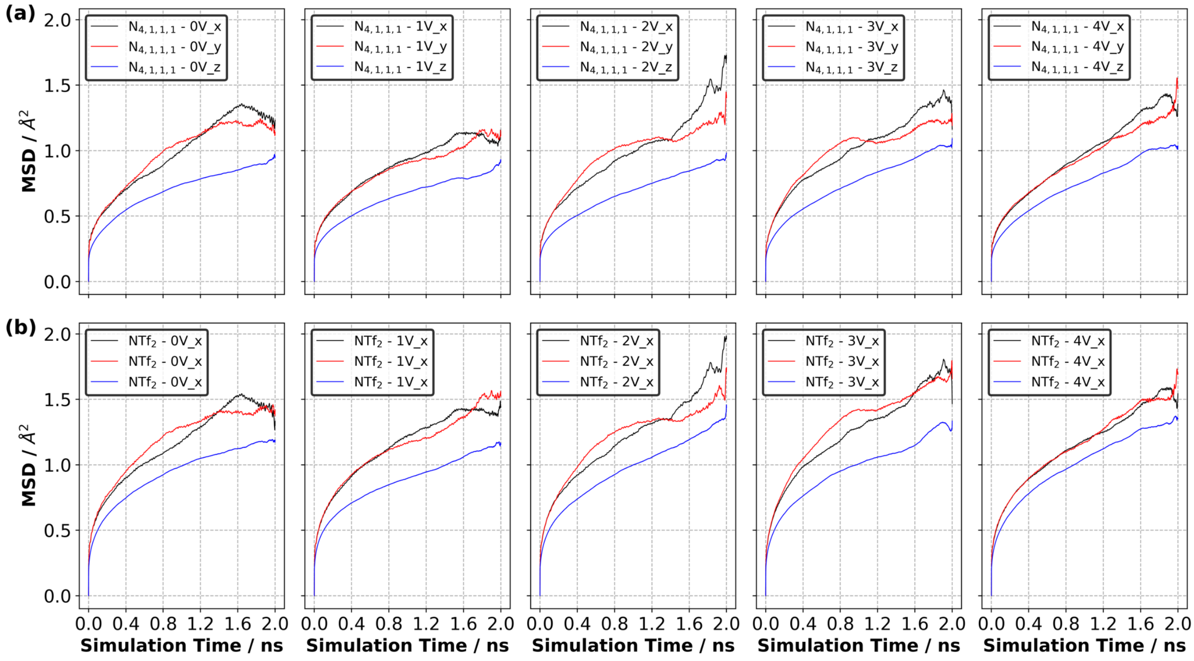

**Figure S43.** Calculated mean square displacement (MSD) at 294 K in each principal direction for (a) $[N_{4,1,1,1}]^+$ and (b) $[NTf_2]^-$ at each $\Delta\Psi$ value (averaged over three independently generated samples).

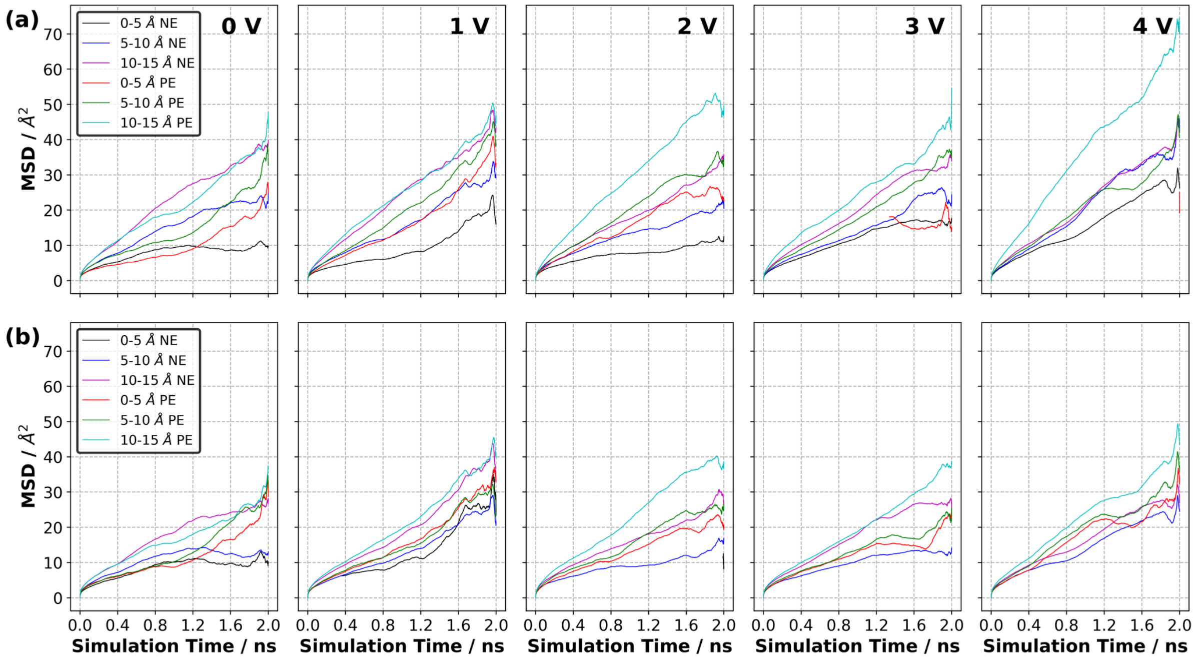

**Figure S44.** Calculated local mean square displacement (MSD), as defined in the main text, for ions in the first layer (0-5 Å), second layer (5-10 Å) and third layer (10-15 Å) from each electrode surface at various $\Delta\Psi$ values at 294 K for (a) $[C_2mim]^+$ and (b) $[NTf_2]^-$ (averaged over three independently generated sample). NE and PE stand for negative electrode and positive electrode, respectively.

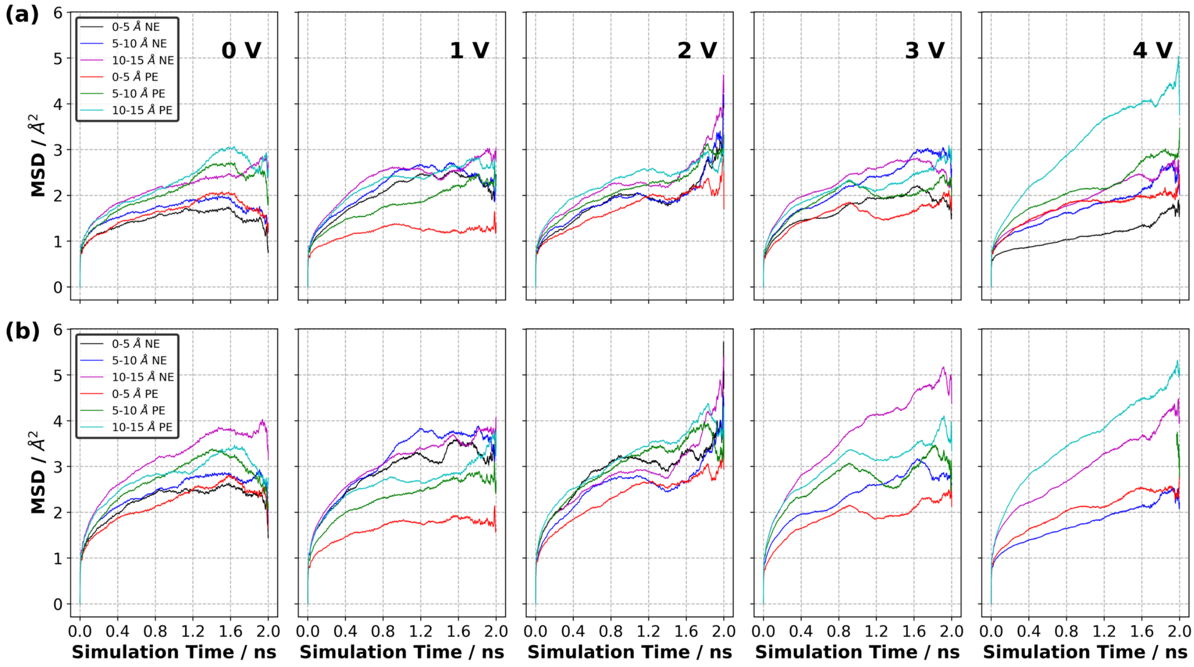


**Figure S45.** Calculated local mean square displacement (MSD), as defined in the main text, for ions in the first layer (0-5 Å), second layer (5-10 Å) and third layer (10-15 Å) from each electrode surface at various $\Delta\Psi$ values at 294 K for (a) $[N_{4,1,1,1}]^{+}$ and (b) $[NTf_2]^{-}$ (averaged over three independently generated sample). NE and PE stand for negative electrode and positive electrode, respectively.

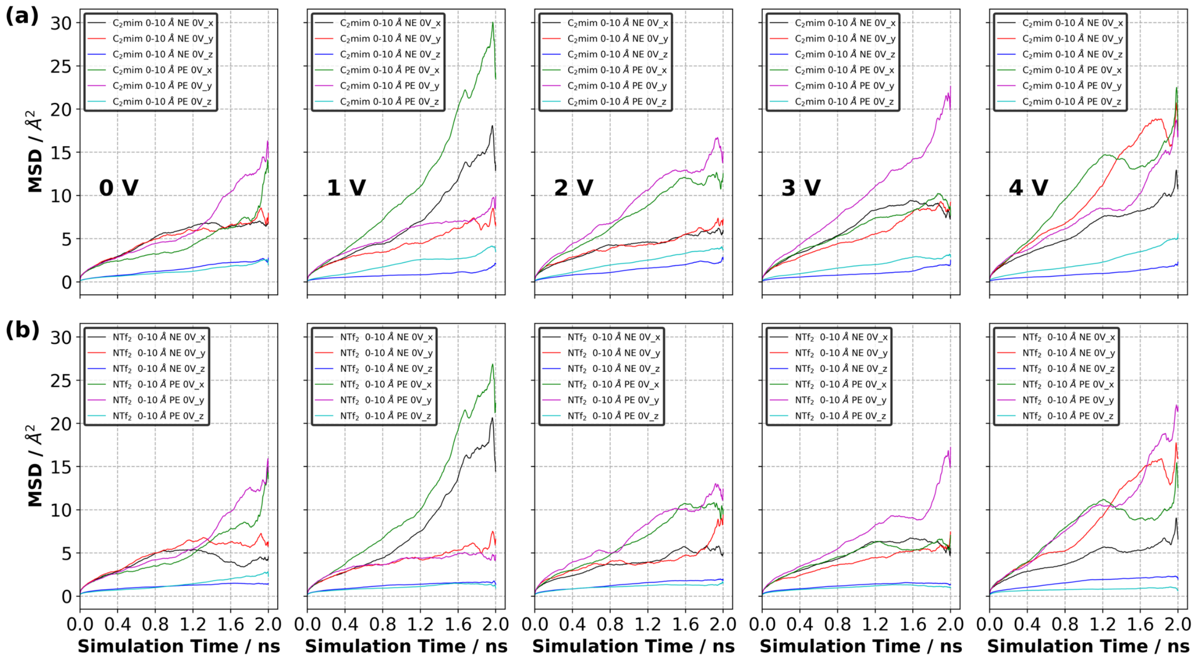


**Figure S46.** Calculated local mean square displacement (MSD), as defined in the main text, for ions in the layer (0-10 Å) in each principal direction from each electrode surface at various $\Delta\Psi$ values at 294 K for (a) $[C_2mim]^{+}$ and (b) $[NTf_2]^{-}$ (averaged over three independently generated sample). NE and PE stand for negative electrode and positive electrode, respectively.

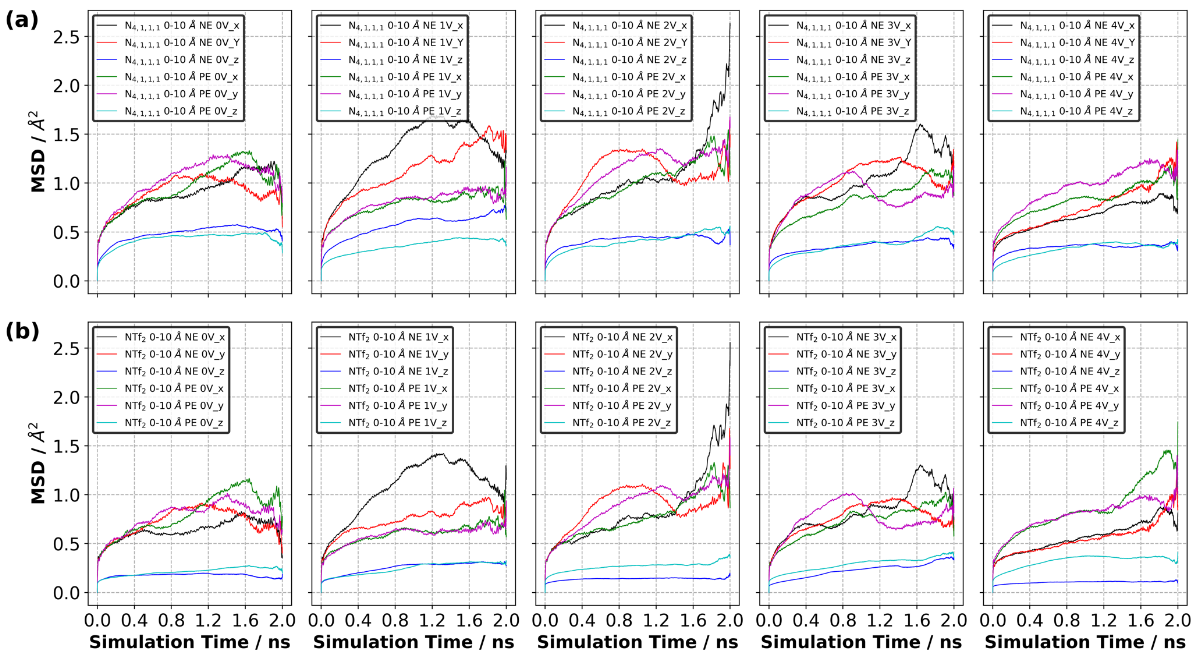


**Figure S47.** Calculated local mean square displacement (MSD), as defined in the main text, for ions in the layer (0-10 Å) in each principal direction from each electrode surface at various $\Delta\Psi$ values at 294 K for (a) $[N_{4,1,1,1}]^+$ and (b) $[NTf_2]^-$ (averaged over three independently generated sample). NE and PE stand for negative electrode and positive electrode, respectively.

### 9. Flux evolution

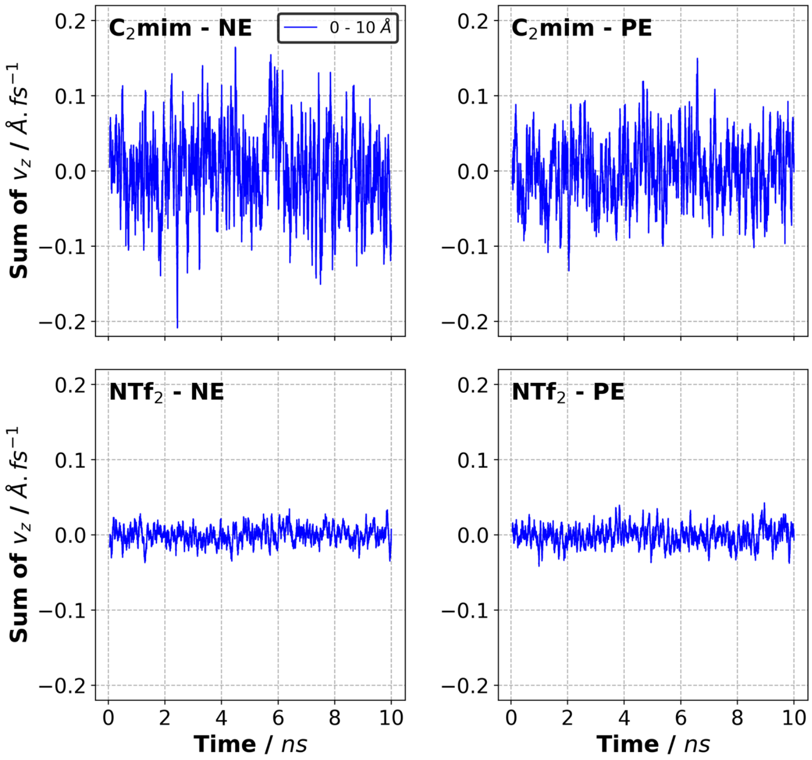


**Figure S48** Sum of the velocities of the atoms in the normal direction to the electrode surface, ($\sum_{i=1}^{N} v_{z,i}$, where *i* and N are the index of atom and total number of atoms for each ion type in the simulation cell, respectively), for ions found within 10 Å of the electrode surface for the [$C_2$mim][$NTf_2$] system at $\Delta\Psi$ = 4 V. The results show the entire simulation time for the charging process. Each data point represents a value averaged over 50 ps. NE and PE represent negative electrode and positive electrode, respectively.

**10. Voltage drop across a supercapacitor.**

The variation in voltage across a supercapacitor can be obtained by solving the Poisson equation:

$$\frac{\partial^2 \Phi(z)}{\partial z^2} = -\frac{1}{\varepsilon_0}\rho(z) \tag{1}$$

which gives:

$$\begin{aligned} \Phi(z) &= -\frac{1}{\varepsilon_0}\int_0^z \int_0^v \rho(u)du dv + C_1 z + C_2 \\ &= -\frac{1}{\varepsilon_0}\int_0^z \int_u^z \rho(u)dv du + C_1 z + C_2 \\ &= -\frac{1}{\varepsilon_0}\int_0^z (z-u)\rho(u)du + C_1 z + C_2 \end{aligned} \tag{2}$$

For a system with electrodes at $z$ = 0 and $z$ = L, the boundary conditions for a constant potential simulation give,

$$\begin{aligned} \Phi(0) &= -\frac{V}{2} = C_2 \\ \Phi(L) &= \frac{V}{2} = \frac{1}{\varepsilon_0}\int_0^L u\rho(u)du + C_1 L - \frac{V}{2} \end{aligned} \tag{3}$$

So,

$$\Phi(z) = -\frac{1}{\varepsilon_0}\int_0^z (z-u)\rho(u)du - \frac{z}{\varepsilon_0 L}\int_0^L u\rho(u)du + \frac{Vz}{L} - \frac{V}{2} \tag{4}$$
